# Integrating network pharmacology and *in silico* analysis deciphers Withaferin-A’s anti-breast cancer potential via hedgehog pathway and target network interplay

**DOI:** 10.1093/bib/bbae032

**Published:** 2024-03-05

**Authors:** Mythili Srinivasan, Apeksha Gangurde, Ashwini Y Chandane, Amol Tagalpallewar, Anil Pawar, Akshay M Baheti

**Affiliations:** Research Scholar, School of Health Sciences and Technology, Dr. Vishwanath Karad MIT World Peace University, Pune, Maharashtra 411038, India; School of Health Sciences and Technology, Dr. Vishwanath Karad MIT World Peace University, Pune, Maharashtra 411038, India; Abhinav College of Pharmacy, Narhe, Ambegaon, Pune, Maharashtra 411041, India; School of Health Sciences and Technology, Dr. Vishwanath Karad MIT World Peace University, Pune, Maharashtra 411038, India; School of Health Sciences and Technology, Dr. Vishwanath Karad MIT World Peace University, Pune, Maharashtra 411038, India; School of Health Sciences and Technology, Dr. Vishwanath Karad MIT World Peace University, Pune, Maharashtra 411038, India

**Keywords:** computational analysis, network pharmacology, *in silico* studies, breast cancer, hedgehog pathway, phytoconstituents

## Abstract

This study examines the remarkable effectiveness of Withaferin-A (WA), a withanolide obtained from *Withania somnifera* (Ashwagandha), in encountering the mortiferous breast malignancy, a global peril. The predominant objective is to investigate WA’s intrinsic target proteins and hedgehog (Hh) pathway proteins in breast cancer targeting through the application of in silico computational techniques and network pharmacology predictions. The databases and webtools like Swiss target prediction, GeneCards, DisGeNet and Online Mendelian Inheritance in Man were exploited to identify the common target proteins. The culmination of the WA network and protein–protein interaction network were devised using Stitch and String web tools, through which the drug–target network of 30 common proteins was constructed employing Cytoscape-version 3.9. Enrichment analysis was performed by incorporating Gprofiler, Metascape and Cytoscape plugins. David compounded the Gene Ontology and Kyoto Encyclopedia of Genes and Genomes, and enrichment was computed through bioinformatics tools. The 20 pivotal proteins were docked harnessing Glide, Schrodinger Suite 2023-2. The investigation was governed by docking scores and affinity. The shared target proteins underscored the precise Hh and WA network roles with the affirmation enrichment *P*-value of <0.025. The implications for hedgehog and cancer pathways were profound with enrichment (*P* < 0.01). Further, the ADMET and drug-likeness assessments assisted the claim. Robust interactions were noticed with docking studies, authenticated through molecular dynamics, molecular mechanics generalized born surface area scores and bonds. The computational investigation emphasized WA’s credible anti-breast activity, specifically with Hh proteins, implying stem-cell-level checkpoint restraints. Rigorous testament is imperative through *in vitro* and *in vivo* studies.

## INTRODUCTION

Cancer a labyrinthine affliction, continues its unsettling surge despite medical strides. Breast carcinoma, a tenacious foe, observed a 0.5% annual upswing from 2011 to 2019 [1]. The North American Association of Central Cancer Registries reported the estimated new cases based on 2005–19 incidence data and the National Center for Health Statistics. Centers for disease control and prevention reported the estimated deaths based on 2006–20 US mortality data. In the United States, breast cancer had an estimated 3,00,590 new cases, with 2,800 occurring in males and 2,97,790 in females. Additionally, both sexes experienced an estimated 43,700 deaths, with 530 in males and 43,170 in females [2]. As per the National Cancer Registry Programme of the National Center for Disease Informatics and Research, under the Indian Council of Medical Research, the estimated cases of breast cancer in women based on 2020 data were 7,12,758. Breast cancer is the leading cancer type in India, with a higher number of cases in urban areas [3]. There is 1.9% yearly decrease in mortality since 2011, attributed to early detection and advanced therapies [4]. Persistent disparities reveal a 40% mortality rate among black women [1]. Women under 50 years old face heightened risk, confronting aggressive triple-negative breast cancer (TNBC) tumors [1]. Amidst this intricate landscape, plant-derived compounds show promise; phytoconstituents unveil low-risk, high-reward avenues for anti-cancer measures, curtailing toxicity [5]. Withaferin-A (WA), a withanolide from *Withania somnifera*, demonstrates proficiency in hindering tumor proliferation, initiating programmed cell death (apoptosis) and disrupting angiogenesis in various types of cancers [6]. Extensive research has shown WA’s potential in countering TNBC in both *in vitro* and *in vivo* models, and its combination with chemotherapy enhances effectiveness [7]. In a study on MDA-MB-231 breast cancer cell lines, WA’s blockade of TASK-3 channels, potentially reducing cancer cell viability, was noticed [8]. WA inhibits key molecules such as NF-kβ, STAT, Hsp90, ER-α, p53 and TGF-β, impeding cancer cell proliferation and causing G2/M cell cycle arrest, ultimately inducing apoptosis. Notably, WA triggers apoptosis through the generation of reactive oxygen species, activation of Par-4, induction of endoplasmic reticulum stress and p53 activation, underscoring its pro-apoptotic properties [9].

The intricate interplay between WA’s direct target proteins and hedgehog (Hh) pathway components takes center stage in this study, exploring their dynamic interactions and roles in stem cell regulation, proliferation and metastasis in the context of breast cancer mitigation. The Hh pathway has been implicated in breast cancer initiation and metastasis [10]. The predominant genes and their mechanisms of hedgehog pathway contain (a) sonic hedgehog (Shh), frequently overexpressed in breast malignant cells; (b) patched (Ptch), the principal Hh pathway receptor, constantly diminished in breast carcinoma, ameliorating Hh pathway signaling; (c) smoothened (SMO), another Hh receptor elevating breast cancer cell viability, proliferation and invasion; (d) Gli transcription factors (Gli1, Gli2, Gli3), responsible for the regulation of tumor growth and angiogenesis [11, 12]; (e) Cyclin D1 promotes cell cycle advancement in cancer; (f) vascular endothelial growth factor (VEGF), activated by Gli1, promotes angiogenesis; and (g) DAPK1, a serine/threonine kinase interlinked with apoptosis and other functions leading to epithelial–mesenchymal transition (EMT) and metastasis [13]. Hh signaling in breast cancer stem cells, particularly Gli1 upregulation, drives drug resistance and tumorigenesis [14]. Concurrently, its interaction with Notch signaling amplifies tumor growth, angiogenesis and metastasis [15], while its complex interplay with other pathways shapes drug resistance research [16, 17].

To identify potential targets of WA, 105 genes were compiled using Swiss target prediction, GeneCards and DisGeNet. Simultaneously, 64 Hh network proteins were sourced from GeneCards and Online Mendelian Inheritance in Man (OMIM). By employing Venny-2, 30 common target genes were identified between the Hh and WA datasets, as detailed in Supplementary Table S5. The WA network was established using stitch tool version 5 (http://stitch.embl.de/). Key proteins and genes in the WA network include p53 (TP53), Bcl-2, prostaglandin endoperoxidase synthase-2 (PTGS/COX2), X-box binding protein-1 (XBP1), mitogen-activated protein kinase-8 (MAPK8), protein kinase-c (PRKC), integral membrane protein-2B (ITM2B), uridine phosphorylase-2 (UPP2) and human Pregnane-X receptor, all with roles in tumor regulation and progression [15].

A protein–protein interaction network (PPI) was constructed for 30 common target proteins using String version 11.5 (https://string-db.org) and imported into Cytoscape version 3.9; merging the WA ligand network, the final drug–target network was obtained. The adsorption, distribution, metabolism and elimination (ADME) and toxicity predictions for WA were conducted with SWISS-ADME and ADMET-LAB-2 tools. Docking studies focused on WA-related and hedgehog proteins, including Hh acyltransferase (HHAT-PDB:7QIU), suppressor of the fused (SUFU-PDB:4KMH), glioma-associated proteins (Gli1-PDB:5OMO, Gli2-PDB:7RXO, Gli3-PDB:4BLD), Indian hedgehog (IHH-PDB:3N1O), desert hedgehog (DHH-PDB:3N1G), vascular endothelial growth factor (VEGF, VEGFA-PDB:4WPB, VEGFB-PDB:2VWE), cyclin-dependent kinase-6 (CDK6-PDB:4TTH) and human matrix metalloproteinase (MMP1-PDB:3SHI, MMP2-PDB:1RTG, MMP9-PDB:5CUH) [18], with PDB IDs from RCSB (www.rcsb.pdb). WA network proteins, like prostaglandin synthase-2 (PTGS2/COX2-PDB:1CVU), mitogen-activated protein kinase-8 (MAPK8-PDB:2G01), PRKC apoptosis WT1 regulator protein (PRKC-PDB:2JK9), B-cell lymphoma-2 (BCL-2-PDB:1G5M), transcription factor FosB/JunD (FosB-PDB:5VPB), human Pregnane-X-receptor (ITM2B-PDB:4SOS), mitogen-activated kinase-10 with JNK3 and JNK3A (MAPK10-PDB:4Y5H) and death-associated protein kinase (DAPK1, DAPK-PDB:5AUT) were docked with WA (PCID:265237), standard drugs tamoxifen citrate (TAM) (PCID:2733525) and capecitabine (CAP) (PCID:60953). Docking results guided molecular dynamics (MD) simulations. The *in vitro* and *in vivo* studies remain imperative for confirming WA’s efficacy on stem-cell levels. The work flow diagram of this study is represented as Figure 8.

## SOFTWARE AND METHODS

### Network pharmacology and analysis

The network pharmacology for the ligand WA and its common targets was achieved using different web tools and software and are given in Table 1. The drug (WA) and hedgehog pathway related target prediction, common target mining, preliminary network creation such as PPI and ligand network [18], combined drug–target network construction, network analysis and enrichment analysis [19, 20] were performed.

**Table 1:** Softwares and webtools used for network pharmacology studies

Sr. No	Software/webtool/database name	url	References
1.	Swiss target prediction	https://www.swisstargetprediction.ch/	[21]
2.	GeneCards database	https://www.genecards.org/	[22]
3.	Online Mendelian inheritance of man (OMIM) database	https://www.omim.org/	[23]
4.	DisGeNet database	https://www.digenet.org/	[24]
5.	PubChem database	https://pubchem.ncbi.nlm.nih.gov/	[25]
6.	Venny 2.0	https://bioinfogb.cnb.csic.es/tools/venny/index2.0.2.html/	[26]
7.	Stitch tool	http://stitch.embl.de/	[27]
8.	String tool	https://string-db.org/	[28]
9.	Cytoscape-3.9	https://Cytoscape.org/	[29]
10.	Metascape	http://Metascape.org/	[30]
11.	Gprofiler	https://biit.cs.ut.ee/gprofiler	[31]
12.	David	http://david.ncifcrf.gov/tools.jsp	[32]
13.	Bioinformatics web tool	https://bioinformatics.com.cn/	[33]
14.	ShinyGo 0.77	http://bioinformatics.sdstate.edu/go/	[34]
15.	MCODE	ftp://ftp.mshri.on.ca/pub/BIND/Tools/MCODE	[35]

#### Disease and pathway target collection

The active targets of WA were obtained from Swiss-target prediction (www.swisstargetprediction.ch/) [21]. The breast cancer genes were retrieved from DisGeNet (https://www.digenet.org) [24] and GeneCards (https://www.genecards.org) [22]. A total of 191 genes from the Swiss-target prediction web tool, 184 breast cancer from DisGenet and 200 breast cancer genes from GeneCards were retrieved and made into dataset 1 with 105 genes altogether (Supplementary Table S1). A compilation of 200 genes associated with the Hh pathway was sourced from the OMIM (https://www.omim.org) database [23], supplemented by an additional 100 Hh-related genes from GeneCards web tool. From these datasets, a subset of 64 genes was characterized by their dual involvement in both pathways, as enlisted in Supplementary Table S2.

#### Common target mining

**Figure 1:**
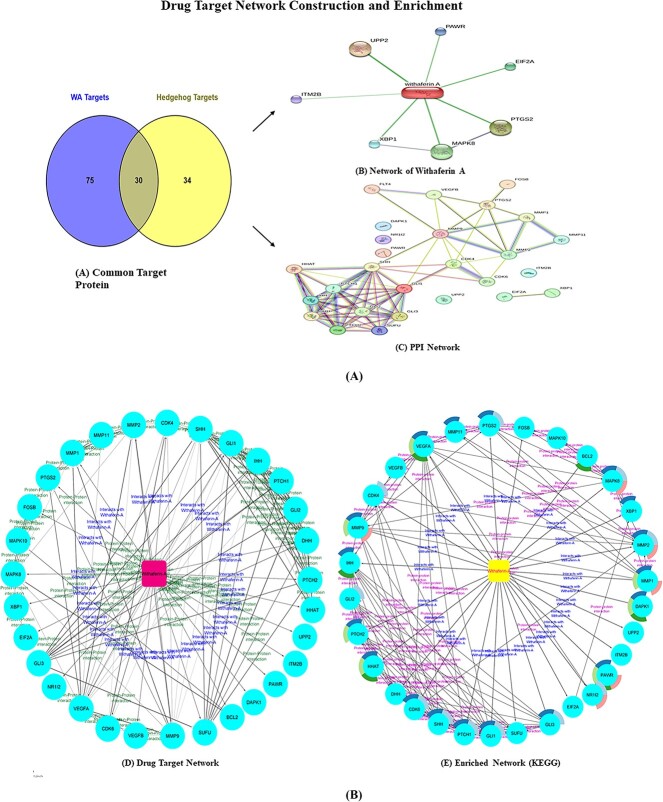
(**A**) Preliminary networks for the drug–target network construction. (a) Common target proteins obtained from Venny.2, (b) Network of WA. (c) PPI network of common target proteins. (**B**) Drug–target network of WA with common target proteins. (a) Drug–target network. (b) Enriched network highlighted with enrichment split doughnut pie charts.

The obtained datasets were incorporated in Venny-2.1.0 web tool (https://bioinfogb.cnb.csic.es/tools/venny/index.html). A total of 30 common targets were retrieved from the two datasets (Figure 1A). The common target details are illustrated in Supplementary Table S3.

#### Preliminary network construction

The WA network was constructed using the Stitch tool (https://stitch.embl.de) [27]. The common intersecting target network (PPI) [36] was created using the string web tool (https://string-db.org) [28]. The TSV files of both networks (Figure 1A was imported into Cytoscape ver. 3.9 (https://cytoscape.org) for the final drug–target network construction [29].

#### Drug–target network construction and analysis

Preliminary networks, saved as TSV files, were imported into Cytoscape 3.9 (https://cytoscape.org) and merged to create a unified network. Unwanted genes were eliminated, focusing on the common targets. Enrichment analysis was executed via Metascape (https://metascape.org) [30], Gprofiler [31] and Functional Enrichment Collection App-2, integrated into Cytoscape ver. 3.9 for convenience from the app manager. The layout and network options were selected. From the 552 enriched pathways, eight significant KEGG pathways [37] were filtered and visually represented as a split doughnut pie chart within the network using a different color palette (Figure 1B) [38, 39]. The purpose of drug–target enrichment is to unveil significant therapeutic targets, offering intricate guidance for drug discovery and development, with a special emphasis on drug–target checkpoints. In addition, the enrichment analysis provided insights for predicting the role of WA on the Hh pathway along with the role of WA’s own target genes and its interplay with Hh proteins and genes.

#### Gene Ontology and KEGG pathways: enrichment analysis

**Figure 2:**
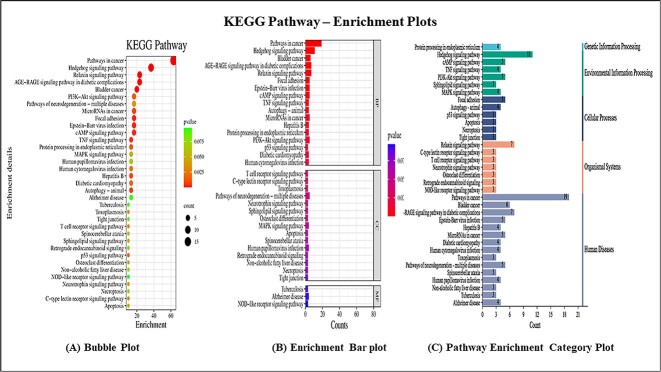
KEGG enrichment pathway plots. (**A**) Bubble plot. (**B**) Enrichment bar plot. (**C**) Pathway enrichment category plot.

**Figure 3:**
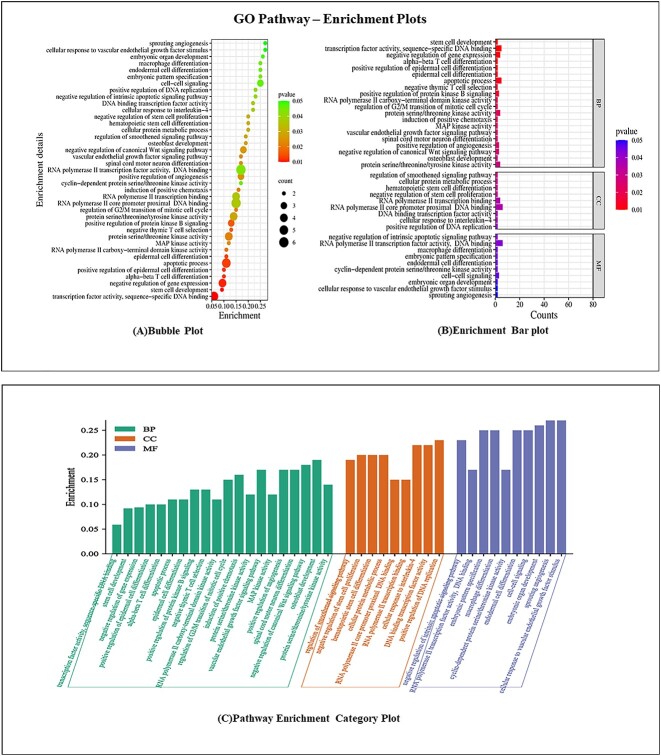
(**A**) Go pathway enrichment plots. (a) Bubble plot. (b) Enrichment bar plot. (**B**) Go pathway enrichment plots. (**C**) Pathway enrichment category plot.

The potential common targets underwent GO and KEGG enrichment analysis through the ‘functional annotation’ feature on the David website (https://David.ncifcrf.gov/tools.jsp) [32]. The collected data were organized and visualized using diverse enrichment plots, including bubble plots, enriched horizontal bars and pathway enrichment category plots, facilitated by the Bioinformatics web tool (https://bioinformatics.com.cn) [33]. The datasets were filtered with a significance threshold of *P* < 0.075, encompassing the top 40 pathways. Categorization into biological process (BP), cellular component (CC) and molecular function (MF) was performed. David enrichment analysis serves the purpose of elucidating and characterizing the functional relevance of a group of genes or proteins, in this context, the potential targets under investigation. David accomplished this by evaluating their involvement in distinct biological pathways and processes by exploring BPs, MFs and CCs. Further, the analysis provided insights on gene count and their significance in specific pathways alongside their functional categories. This analysis provided a comprehensive understanding of the functional roles and implications of the selected genes and proteins within the specific context of the study, thus enhancing the overall interpretation of the data and its biological significance. The enrichment plots of both KEGG and GO pathways are represented in Figures 2 and 3A and B, respectively.

#### Drug–target network clustering and topology analysis

**Figure 4:**
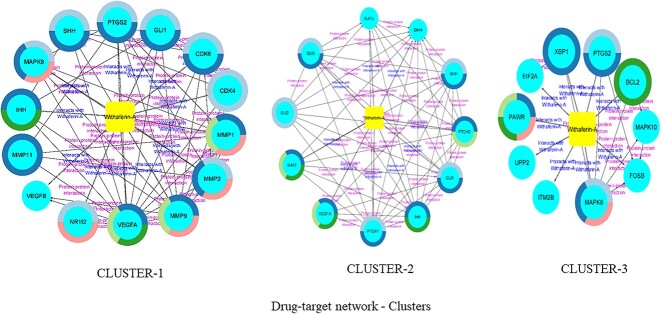
Drug–target network—clusters.

The clustering and topology analysis of the drug–target network was performed using the MCODE plugin [35] within the Cytoscape software. The algorithm employs a three-stage process; firstly, nodes are weighted based on the interconnectedness of their neighbors, assigning higher scores to more densely connected nodes. Subsequently, molecular complex prediction begins with the highest-weighted node (seed), iteratively expanding the complex by incorporating nodes exceeding a predefined threshold. Finally, post-processing filters, including haircut and fluff, are applied to enhance the quality of the identified clusters. For the clustering, the network data were imported into Cytoscape, followed by the initiation of the MCODE algorithm, where parameters such as the node score cutoff (0.2), K-Core cutoff (2), maximum depth (100) and degree cutoff (2) were adjusted. The haircut filter was enabled. The results, highlighting clusters based on their MCODE scores, were then analyzed. The clusters were visually explored, considering their interconnections and their biological significance. A total of three clusters were obtained on analysis; their topology details were obtained on individual cluster analysis using MCODE and Network Analyzer version 4.8.4 within Cytoscape as plugins. The clusters are represented in Figure 4, and the topology details are listed in Table 3.

#### Path view enrichment diagram prediction for the Hh pathway

**Figure 5:**
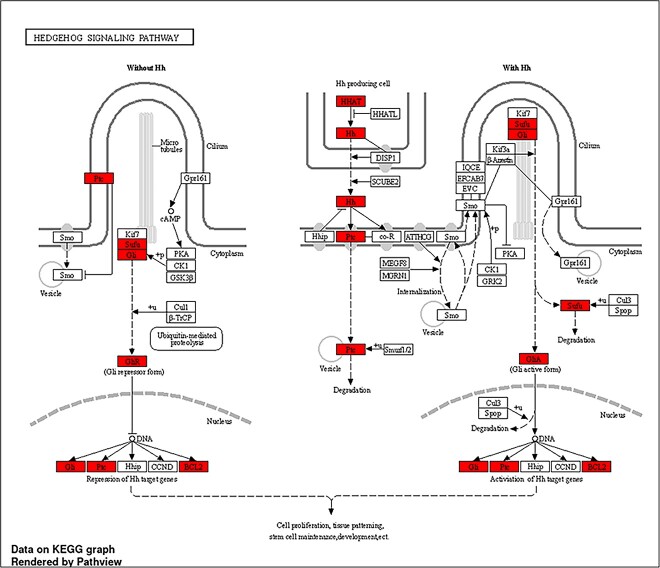
Path view enrichment diagram for the Hedgehog pathway (Pathway ID: hsa04340) from KEGG pathways.

The Hh pathway enrichment diagram was obtained by the inclusion of the common target genes or proteins in the ‘path-view’ utility within Shinygo-ver.0.77 (http://bioinformatics.sdstate.edu/go/), a web-based analytical tool recognized for its proficiency in gene enrichment analysis. Shiny Go serves as a graphical instrument, adeptly annotating the input genes by referencing string-db ver. 11 [34]. The web tool constructs pathway diagrams, wherein pathway enrichment is effectuated by activating the ‘path view’ option. The diagram distinctively highlights the relevant pathway genes through a discernible red hue. In the specific context of the KEGG pathway, the selection was directed toward the Hh pathway (Pathway ID: hsa04340) illustrated in Figure 5.

### Molecular docking of key targets

Software and web tools: Maestro 13.6 (Schrodinger Suite 2023-version 2) [40] for protein and ligand preparation, Glide (Schrodinger Suite 2023-2) for molecular docking, Desmond-2020-ver.1 [41] for simulation studies were used.

#### Key target proteins: preparation

The 20 designated key target protein PDB files (7QIU, 4KMH, 5OMO, 4BLD, 3N1O, 3N1G, 4WPB, 2VWE, 4TTH, 3SHI, 1RTG, 5CUH, 1CVU, 2G01, 2JK9, 1G5M, 5VPB, 4SOS, 4Y5H and 5AUT) were obtained from the Protein data bank (PDB) database (https://www.rcsb.org/). These acquired proteins were imported into the protein preparation wizard (PPW) of Schrodinger Suite 2023-2. Intricate modifications such as the addition of hydrogens, bond order assignment, optimization of hydrogen positions and appropriate side chain corrections were incorporated. Energy minimization in the proteins was kept optimal by keeping the backbone atoms firm and the side chain atoms flexible. The process addressed missing structural elements, such as loops and disulfide bonds. Notably, pH settings were fine-tuned to emulate physiological conditions, at 7.0. Missing side chains were introduced for the proteins 5OMO, 2VWE, 1CVU and 4TTH utilizing the prime module of Schrodinger suite. In the process of protein refinement, water molecules located beyond a distance of 5 Å were systematically removed, and metals were generated with zero-order bonds. Following the elimination of water molecules, any remaining waters were further filtered, ensuring a minimum distance of less than 3 Å between hydrogen atoms and non-water entities. For the subsequent energy minimization step, the OPLS3e force field and optimization algorithm were engaged. To enhance convergence, a heavy atom root mean square deviation (RMSD) threshold of 0.30 Å was set, ensuring the optimization process was sufficiently precise [26, 41, 42].

#### Ligand preparation

The ligands WA, TAM and CAP were obtained from the PubChem database (https://www.pubchem.ncbi.nlm.nih.gov/) [25] with the respective PubChem IDs 265237, 2733525 and 60953 in SDF format. These ligand structures were subsequently stabilized and meticulously prepared using the LigPrep tool of the Schrodinger suite 2023-2. Here, the diverse ionization states, stereoisomers and tautomers of the ligands were achieved. The ligands were prepared for their three-dimensional (3D) coordinates by the addition of hydrogen, energy minimization and enabling a proper force field [41, 43, 44].

#### Receptor grid generation and docking

The receptor grid generation tool of Schrodinger Suite 2023-2 was used to create a receptor grid. The grid resolution, center coordinates and grid box dimensions were obtained from this tool. The grid generation characterizes the active site vicinity through coordinates (*x*, *y*, *z*). Grid box dimensions were based on active site ligands (reference ligands) for specific proteins: SO₄¯ for 5OMO, Serine for 4BLD, 2AV for 4TTH, CA305 for 3SHI, CA5 for 1RTG, LTQ for 5CUH, arachidonic acid for 1CVU, 73Q for 2G01, MG1144 for 2JK9 and Gly205Ser for 1G5M. For remaining proteins, the potential binding surfaces were predicted using the Sitemap option of Schrodinger Suite 2023-ver.2. This tool, with its sitemap score, provided binding site details with properly highlighted areas. The discovery of potential alternative binding sites or the validation of existing binding sites was achieved through sitemap results. Further, the confirmation of binding site residues for proteins with reference ligands was done using the PDBsum web tool (https://ebi.ac.uk/), supplemented by a comprehensive literature review. The grid details are enumerated in Supplementary Table S4.

#### Molecular docking studies of key target proteins

The Glide tool of Schrodinger Suite 2023-2, utilizing the HTVS (high-throughput virtual screening) algorithm and OPLS3e force field in standard precision (SP) mode, executed rigid receptor docking. Here, the docking method (standard precision and extra precision), parameters and scoring functions were all defined as docking options. The number of generated postures and the preferred sample methods were among the parameters set for every docking run [45]. An initial docking computation produced various ligand poses within the binding site, which were then scored using the selected scoring function. During docking, key protein conformations remained fixed while ligands exhibited flexibility [46] Comparative docking outcomes of ‘WA’ and standard drugs ‘TAM’ and ‘CAP’ were evaluated using Maestro ver. 13.6 of Schrodinger Suite 2023-2. Comprehensive details regarding the docking study and respective glide scores are thoroughly outlined in Table 4. Additionally, the visual depictions of the two-dimensional (2D) and 3D structures of the protein–ligand complex are illustrated in Supplementary Table S5.

### Molecular simulation studies

MD simulations were conducted using Desmond 2020.1 from Schrödinger [41]. The focus was on the docked molecular complexes: 2VWE paired with WA, 2VWE paired with TAM, 3N1O paired with WA, 3N1O paired with TAM, 4KMH paired with WA, 4KMH paired with TAM, 4WPB paired with WA, 4WPB paired with TAM and 2JK9 paired with WA, 2JK9 with CAP. The OPLS-2005 force field [47] and explicit solvent model with the TIP3P water molecules in a 10 × 10 × 10 Å periodic boundary solvation were employed. Protein–ligand complex preprocessing, optimization and minimization were performed using the protein preparation wizard. The system builder tool was used for system construction [48]. Equilibration was executed under an Normalization Visualization tool (NVT) ensemble for 10 ns to stabilize the protein–ligand complexes, followed by a brief equilibration and minimization in an Nuclear non-Proliferation Treaty (NPT) ensemble for 12 ns. The Nose–Hoover chain coupling scheme maintained a temperature of 300 k and 1 atm pressure throughout the simulation [40, 49]. Long-range electrostatic interactions were computed using the particle mesh Ewald method with a 9 Å coulomb interaction radius. A 2-fs time step and Martyna–Tuckerman–Klein chain coupling scheme were employed for pressure control [48, 50].

A 100 ns production run ensured, with simulation stability assessed through root mean square deviation (RMSD), radius gyration (Rg), root mean square fluctuation (RMSF) and hydrogen bond calculations [46]. Binding free energy (ΔGbind) for specified proteins (2JK9, 2VWE, 3N1O, 4KMH and 4WPB) and ligands (WA, CAP and TAM) was computed using the provided formula.


$$ \boldsymbol{\Delta} \mathbf{Gbind}=\mathbf{Gcomplex}-\left(\mathbf{Gprotein}+\mathbf{Gligand}\right) $$

Here, Gcomplex denotes the complex energy of receptor and ligand, Gprotein signifies the receptor energy and Gligand represents the energy of the unbound ligand.

**Figure 6:**
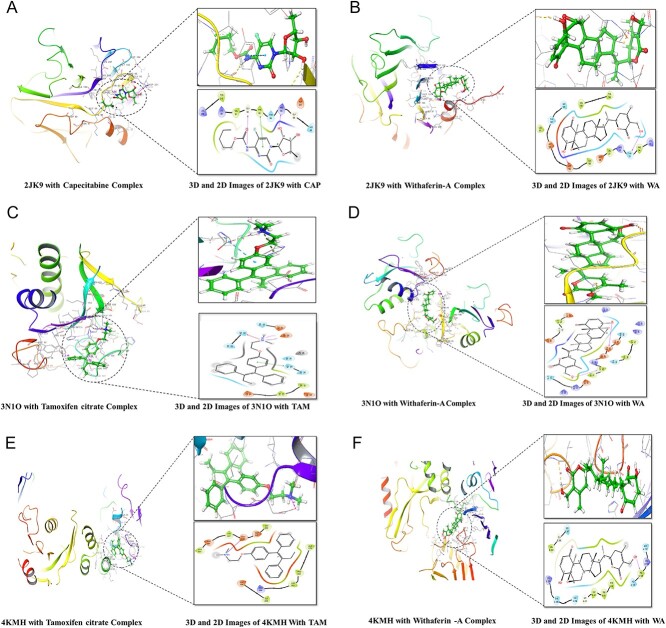
(**A**) Molecular docking image in 2D and 3D formats for the PRKC apoptosis WTI regulator protein (2JK9)-capecitabine complex. (**B**) Molecular docking image in 2D and 3D formats for the PRKC apoptosis WT1 regulator protein (2JK9)-WA complex. (**C**) Molecular docking image in 2D and 3D formats for the Indian hedgehog protein (3N1O)-TAM complex. (**D**) Molecular docking image in 2D and 3D formats for the Indian hedgehog protein (3N1O)-WA complex. (**E**) Molecular docking image in 2D and 3D formats for the Suppressor of fused (SUFU:4KMH)-TAM complex. (**F**) Molecular docking image in 2D and 3D format for the suppressor of fused (SUFU:4KMH) with WA complex.

The simulation visualizations of 2JK9, 2VWE, 3N1O, 4KMH and 4WPB are represented in Figure 6A–E.

### Prediction of ADME and drug likeliness properties of WA

The ADME prospects, as well as drug-likeness properties of WA, were assessed using Swiss ADME [51]. This online tool is known for its accurate, simple, precise and user-friendly interface. The derived results are enumerated in Supplementary Table S6.

### Toxicity screening and acute toxicity prediction

The toxicity prediction of WA was performed using ADMETlab version 2.0 [52]. The toxicity evaluation of various organs, toxicity pathways and toxicophore rules were schematized, and the inferences are enlisted in Supplementary Table S7.

## RESULTS AND DISCUSSION

### Network pharmacology and analysis

#### Disease and pathway target collection

The active targets of WA were identified using the Swiss-target prediction webtool (https://www.swisstargetprediction.ch/) [21], Relevant breast cancer genes were curated from DisGeNet (https://www.disgenet.org) and GeneCards (https: www.genecards.org), with DisGeNet offering comprehensive gene–disease associations and GeneCards providing detailed insights into human genes [20, 53]. The Swiss-target prediction tool provided 191 direct targets, while DisGeNet contributed 184 breast cancer genes, and an additional 200 were acquired from GeneCards. This cumulative dataset of disease significance includes 105 breast cancer genes and proteins that were selected as common targets of WA. The Hh pathway-related genes and proteins were procured from the OMIM database with a 200 gene count [23] and 100 genes from the GeneCards web tool. The selected datasets were used for common target mining.

#### Common target mining

The common target prediction utilized Venny (version 2.0), a user-friendly tool for comparing and visualizing dataset intersections [20]. Venny-2 generated Venn and Euler diagrams, highlighting commonalities and differences between datasets. The dataset of 105 WA-target specific proteins was compared to 64 Hh pathway-related proteins, yielding 30 common target genes. The proteins are listed in Supplementary Table S3.

#### Preliminary network construction

Utilizing the Stitch tool (https://stitch.embl.de) [27], a network pertaining to WA was constructed by inputting the canonical smile format of WA within the chemical option. The resultant network showcased notable proteins, including eucaryotic translation initiation factor (EIF2A), integral membrane protein 2B (ITM2B), MAPK8, pro-apoptotic protein (PAWR), cyclooxygenase/prostaglandin endoperoxidase synthase (PTGS2), uridine phosphorylase-2 (UPP2) and X-box binding protein-1 (XBP1). The network analysis featured seven nodes and two edges, resulting in an enrichment *P*-value of 0.995.

The elevated *P*-value observed in the analysis of the ‘WA network’, utilizing a subset of seven proteins, signifies the provision of a valuable and less noisy dataset tailored for the specific research or analysis. In stark contrast, the expanded network comprising 17 proteins yielded a lower *P*-value is 0.00057. Our unconventional approach which involves favoring a higher *P*-value network, underscores the deliberate selection of a smaller, intricately tailored network. This strategic curation aims to mitigate the influence of biases and accentuate direct interactions within the WA network. The discerning choice of these seven proteins assumes paramount importance in the construction of a precise drug-target network.

Furthermore, the common target genes (30) enlisted in Supplementary Table S3 were made into a protein–protein network (PPI) using the String web tool (https://string-db.org). The network analysis report unveils 27 nodes, 68 edges, an anticipated edge count of 13, a clustering network coefficient of 0.666 and a statistically significant *P*-value of 0.000001. The preliminary networks are depicted in Figure 1A.

#### Drug–target network construction and analysis

The TSV files of both the WA network and the common target PPI network, were imported into Cytoscape ver 3.9. [29]. Employing the network merge tool with the union option, both networks were amalgamated into a drug-target network. The constructed network was given a Y-files circular layout. The proteins were depicted as ellipses in a light blue hue, while the ligand WA was distinctly highlighted with a pink color and an encompassing round rectangle shape. Protein interactions involving WA were adeptly designated with a blue label ‘interacts with WA’, while PPIs were signified with a green label ‘protein–protein interaction’. Edges for WA interactions were delineated with continuous lines and source arrows, while PPI interactions were assigned solid lines with open circles. The network analysis revealed a total of 31 nodes and 116 edges, characterized by an average number of neighbors of 7.484. The network exhibited a diameter of 4 and a radius of 2, a clustering coefficient of 0.666, a network density 0.249, network heterogeneity of 0.714, a solitary connected component and an analysis time of 0.047 s. The significant direct interactions observed with WA involved the pivotal proteins, FOSB, PTCH1, PTCH2, GLI1, GLI3, BCL2, CDK4, MMP2, MMP1, MMP9, SUFU, PTGS2, MAPK8, XBP1, EIF2A, NR1I2, VEGFA, CDK6, DAPK1, PAWR, ITM2B, UPP2 and HHAT. Further, indirect interactions were discerned with DHH, SHH and IHH. Since DHH, SHH and IHH exclusively function through SUFU and GLI proteins in the hedgehog pathway, DHH demonstrates an interaction with GLI3 in the context of WA. In contrast, IHH and SHH encage with SUFU and GLI3 for their interaction with ligand WA.

The enrichment analysis of the drug-target network was executed using Metascape, Gprofiler and Functional Enrichment Collection App-2, seamlessly integrated into Cytoscape ver 3.9 via its app manager plugin tool. It generated a substantial corpus of a total of 552 pathway enrichment files, with a discerning focus on KEGG pathways. The top eight selected KEGG pathways (*P*-value < 0.2) were portrayed within an enrichment pie chart harmoniously incorporated into the network, showcasing a spectrum of diverse color palettes (Table 2). Notably, the ligand WA was highlighted with a yellow tint. The edges of WA interactions were rendered as contagious lines complemented by source arrows, and the PPI interactions were distinctively symbolized by solid lines accompanied by open circles. The enriched proteins observed are PTGS2, MMP11, VEGFA, MMP9, IHH, GLI2, PTCH2, HHAT, CDK6, SHH, PTCH1, GLI1, NR1I2, PAWR, DAPK, MMP1, MMP2, XBP1, MAPK8 and BCL2. The hedgehog pathway, pathways in cancer and the Relaxin signaling pathway stood out as top contenders among eight shortlisted KEGG pathways, with low *P*-values of 1.1102E−16, 2.2E−16 and 3.36E−06, respectively. Here, the enrichment *P*-value significance of WA was compared with the study on standard drug CAP, *P*-value ranging from 0.02 to 0.06 in single administration and combination therapy in TNBC [54]. Enriched proteins in hedgehog and cancer pathways were prioritized for subsequent docking. The drug–target network and enriched pathway network are depicted in Figure 1B.

**Table 2:** KEGG pathway (top eight) selected for network enrichment

Pathway ID	Pathway name	Precision	*P*-value	Intersecting genes	Color given in doughnut pie chart of network
KEGG-04340	Hedgehog signaling pathway	0.458	1.1102E−16	11	Light blue
KEGG-05200	Pathways in cancer	0.75	2.2E−16	19	Dark blue
KEGG-04926	Relaxin signaling pathway	0.292	3.66E−06	7	Light green
KEGG-04657	IL-17 signaling pathway	0.25	1.18E−05	6	Dark green
KEGG-05205	Proteoglycans in cancer	0.25	1.35E−03	6	Pink
KEGG-05212	Pancreatic cancer	0.167	4.05E−03	4	–
KEGG-04215	Apoptosis pathway	0.125	6.63E−03	4	–
KEGG-04024	c-AMP signaling pathway	0.208	2.56E−02	5	–

#### GO and KEGG pathways: enrichment analysis

David Tool (v6.8) enabled functional annotation: tables, charts and clusters were extracted [32]. The top 35 KEGG pathways were selected from 381 charts in 11 clusters, based on *P* < 0.075. Visualization via bubble plot enriched horizontal bars and pathway category plots with the SR plot option (bioinformatics.com.cn) [33].

Upon analyzing the bubble plot, the pathways in cancer and the hedgehog signaling pathway emerged as notably significant (*P* < 0.025), with gene counts exceeding 10. Similarly significant were the Relaxin signaling pathway, diabetic pathway, bladder cancer, micro RNAs in cancer, focal adhesion, cAMP signaling pathway and TNF signaling pathway (*P* < 0.025), each with a gene count of 10 or fewer. The MAPK and HPV signaling pathways held intermediate significance (*P* < 0.05), while the P53 signaling pathway and apoptosis pathway secured positions under the *P*-value benchmarks (*P* < 0.025, 0.05), each with a gene count of five or fewer. The enrichment bar plot highlighted highly significant pathways (*P* < 0.025) within the BP category: pathways in cancer, hedgehog, bladder cancer, P53 signaling, TNF signaling, autophagy and microRNAs in cancer. All plots are depicted in Figure 2.

Similarly, 39 GO pathways exhibited a *P*-value < 0.05 from the extensive pool of 381 annotations. The dataset is systematically transformed into visually insightful depictions, including a bubble plot, an enrichment bar plot infused with distinct colors and pathway enrichment category plots. Upon analysis of the bubble plot, specific GO pathways such as apoptosis, positive regulation of epidermal cell differentiation, negative regulation of gene count, stem cell development and transcription factor activity, coalesced within the echelons of heightened significance (*P*-value < 0.02), where the gene count was conservatively capped at 6 or fewer. Conversely, the remaining pathways assumed a stance of moderate significance (*P*-value < 0.05). Subsequently, the analysis of the enrichment bar plot unveiled valuable insights into the domain of BPs, illuminating pivotal pathways such as stem cell development, negative regulation of gene expression, the apoptotic process, positive regulation of protein kinase B signaling, positive regulation of angiogenesis and VEGF signaling pathway, all characterized by a *P*-value <0.02. The visualizations of the GO pathway is depicted in Figure 3A and B.

#### Drug–target network clustering and topology analysis

The MCODE plugin was utilized within Cytoscape for clustering analysis with parameters specific to the MCODE algorithm and Network Analyzer 4.8.4. Three distinct clusters were identified, characterized by their scores, node composition and edge connectivity. Cluster 1, consisting of 15 nodes and 54 edges, demonstrated a modularity of −1.317, an indegree of 54 and an outdegree of 41, with a clustering coefficient of 0.814. Similarly, cluster 2, composed of 12 nodes and 55 edges, exhibited a modularity of −1.618, an indegree of 55 and an outdegree of 34, within a network density of 0.833 and a clustering coefficient of 0.877. Cluster 3, encompassing 11 nodes and 18 edges, displayed a modularity of −0.692, an indegree of 18 and an outdegree of 26, with a clustering coefficient of 0.482. All clusters were identified as part of a single connected component. Notably, the proteins present in cluster 1 included SHH, PTGS2, GLI1, CDK6, CDK4, MMP1, MMP2, MMP9, VEGFA, NR1I2, VEGFB, MMP11, IHH, MAPK8 and WA; cluster 2 contained SUFU, DHH, SHH, PTCH2, GLI1, IHH, PTCH1, VEGFA, HHAT, GLI2, GLI3 and WA; and cluster 3 comprised XBP1, PTGS2, BCL2, MAPK10, FOSB, MAPK8, ITM2B, UPP2, PAWR, EIF2A and WA (Figure 4).

The topological coefficients for the cluster 1 proteins reveal distinct values for each. Notably, NR1I2 exhibited the highest topological coefficient at 0.928571429, indicating a high level of connectivity in the network. Additionally, WA displayed a substantial topological coefficient of 0.5119047. The proteins MMP1, MMP2 and MMP9 demonstrated intermediate topological coefficients of 0.479591, 0.60317 and 0.535714, respectively. In cluster 2, VEGFA displayed the highest topological coefficient at 0.94545, suggesting a central and highly connected position within the network. GLI2 and HHAT also exhibited substantial coefficients of 0.92045 and 0.93506, respectively, indicating significant network centrality. Additionally, WA demonstrated a notable topological coefficient of 0.838383, further contributing to its connectivity in the analyzed network. In cluster 3, MAPK10 displayed the highest topological coefficient at 0.7222222, indicating a significant level of connectivity. EIF2A and BCL2 also exhibited substantial coefficients of 0.666666 and 0.6, respectively. Conversely, ITM2B, UPP2 and PAWR demonstrated lower or zero coefficients, suggesting comparatively less connectivity within the network. The topology details are listed in Table 3.

**Table 3:** Topological parameters of clusters

Cluster 1						
Protein name	Average shortest path length	Clustering coefficient	Neighborhood connectivity	Radiality	Topological coefficient	Score
MMP11	1.714285714	1	9.25	0.948979592	0.660714286	4
CDK6	1.5	0.857142857	9.571428571	0.964285714	0.683673469	4.464285714
PTGS2	1.5	0.857142857	9.142857143	0.964285714	0.653061224	6
GLI1	1.5	0.857142857	9	0.964285714	0.642857143	8
MAPK8	1.571428571	1	10.16666667	0.959183673	0.726190476	6
MMP2	1.357142857	0.694444444	8.444444444	0.974489796	0.603174603	6
SHH	1.571428571	0.866666667	8.666666667	0.959183673	0.619047619	8
MMP9	1.142857143	0.545454545	7.5	0.989795918	0.535714286	6
WA	1.142857143	0.484848485	7.166666667	0.989795918	0.511904762	7
Cluster 2						
GLI2	1.272727273	1	10.125	0.975206612	0.920454545	8
DHH	1.090909091	0.888888889	9.5	0.991735537	0.863636364	8
HHAT	1.363636364	0.952380952	10.28571429	0.966942149	0.935064935	5.785714286
GLI1	1	0.8	9	1	0.818181818	8
IHH	1	0.8	9	1	0.818181818	8
VEGFA	1.545454545	0.9	10.4	0.950413223	0.945454545	6
SHH	1.090909091	0.8	9.1	0.991735537	0.827272727	8
GLI3	1.181818182	0.944444444	9.888888889	0.983471074	0.898989899	8
PTCH1	1	0.8	9	1	0.818181818	8
PTCH2	1.090909091	0.888888889	9.5	0.991735537	0.863636364	8
SUFU	1.181818182	0.944444444	9.888888889	0.983471074	0.898989899	8
WA	1.181818182	0.805555556	9.222222222	0.983471074	0.838383838	7
Cluster 3						
ITM2B	2	0	9	0.888888889	0	0
FOSB	1.6	0.666666667	5.25	0.933333333	0.525	3
PTGS2	1.7	1	6.333333333	0.922222222	0.633333333	6
EIF2A	1.9	1	6	0.9	0.666666667	2
MAPK8	1.4	0.466666667	4.166666667	0.955555556	0.416666667	6
UPP2	2	0	9	0.888888889	0	0
BCL2	1.7	0.666666667	6	0.922222222	0.6	1.666666667
MAPK10	2.1	0.666666667	4.333333333	0.877777778	0.722222222	1.666666667
PAWR	2	0	9	0.888888889	0	0
XBP1	1.7	0.666666667	5.666666667	0.922222222	0.566666667	1.666666667
WA	1.1	0.166666667	2.666666667	0.988888889	0.333333333	7

#### Path view enrichment diagram prediction for Hh pathway

The path view tool of Shiny Go ver. 0.77, as employed, serves as a graphical interface facilitating the visual representation of intricate molecular pathways, thus enabling a comprehensive understanding of gene interactions and their dynamic role in BPs [34]. The path view diagram depicting the Hh pathway (Pathway ID: hsa04340) was meticulously selected from the enriched KEGG pathways attained via ShinyGo ver.0.77. Upon careful examination, it became apparent that upon activation of hedgehog signaling, a set of genes, including HHAT, SUFU, PTCH, Gli A, Gli1, Hh, Gli and BCL2, exhibited notable enrichment and significance. Notably, SUFU and PTCH displayed evidence of degradation, concurrently leading to the activation of GliA and subsequently triggering the activation of downstream Hh target genes. Conversely, in the absence of Hh gene signaling, a noticeable repression of Hh target genes was observed. Hh signaling is essential for development and tissue maintenance but is linked to cancer when dysregulated. The visual representation is provided in Figure 5.

### Molecular docking on key targets

Hedgehog acyltransferase (HHAT): WA (−14.5232 kcal/mol) and TAM (−15.3053 kcal/mol) compete favorably. WA strengthens Phe143’s stability at the binding site by forming a hydrogen bond with it. WA (−14.9937 kcal/mol) has greater affinity for the SUFU than TAM (−12.9431 kcal/mol). WA and Glu448 interact through a salt bridge.

Glioma-associated protein 1 (Gli1): WA (−13.4295 kcal/mol) makes aromatic bonds with Ser521, which accelerates its affinity, whereas TAM (−16.7434 kcal/mol) forms hydrogen bonds with Leu526, which augments its affinity. The affinities of WA (−0.3396 kcal/mol) and TAM (−7.4834 kcal/mol) for glioma-associated protein 3 (Gli3) differ enormously, and WA forms a hydrogen bond with Leu480. Indian hedgehog (IHH): Through a variety of interactions, including hydrogen bonds with Asp152 and π-cations with His185, WA (−17.6299 kcal/mol) overcomes TAM (−15.0456 kcal/mol). Desert hedgehog (DHH): TAM (−9.8587 kcal/mol) connects with Glu800, whereas WA (−7.5611 kcal/mol) binds to Pro716. Vascular Endothelial Growth Factor-B (VEGF-B): WA (−13.9781 kcal/mol) and TAM (−11.1113 kcal/mol) are comparable in this regard. Thr31 and Leu66 create hydrogen bonds with WA. Cyclin-dependent Kinase-6 (CDK6): TAM (−18.6402 kcal/mol) encases Asp104 and forms a π-stacking with His100, whereas WA creates a hydrogen bond with Asp104. WA (−6.5331 kcal/mol), like CAP (−13.2186 kcal/mol), establishes hydrogen bonds with Met111 in MAPK-8. WA (−14.0674 kcal/mol) collaborates with Asp182 and Thr35 in the PRKC Apoptosis WT1 Regulator Protein (PAWR), whereas TAM (−14.1179 kcal/mol) establishes hydrogen bonds with Gly92 and Tyr93. Compared to CAP (−20.9354 kcal/mol), WA (−13.3983 kcal/mol) forms hydrogen bonds with Asn11 and Asp196 in BCL2. WA (−2.41 kcal/mol) encases hydrogen bonds with Arg292 and Cys285 with transcription factor FOSB/JUND, while CAP (−5.32 kcal/mol) interacts with Cys285 for the same factor. Pregnane X Receptor: WA (−10.4444 kcal/mol) collaborates with Leu240 through a hydrogen bond, while CAP (−15.4818 kcal/mol) encages Leu209. Mitogen-activated kinase-10 (MAPK10): similar to CAP (−14.9197 kcal/mol), WA (−8.0208 kcal/mol) forms a hydrogen bond with Met149. Death-associated kinase-1 (DAPK1): WA (−11.7234 kcal/mol) and CAP (−15.3683 kcal/mol) both create hydrogen bonds with Glu143.

In the realm of Hh acyltransferase (HHAT), WA demonstrated a binding score on par with TAM, supported by the formation of a stabilizing hydrogen bond with Phe143 within the binding site. In contrast, a notable salt bridge engagement with Glu448 exhibited heightened binding efficacy for WA compared to TAM with SUFU. Furthermore, TAM produced hydrogen bonds with Leu526 during its interactions with GLI1, whereas WA encompassed the establishment of aromatic bonds with Ser521, bolstering its affinity. Noteworthy interactions surfaced in the case of IHH, where WA’s binding score surpassed that of TAM, due to the convergence of hydrogen bonds with Asp152 and π-cation interactions with His 185. The engagement of WA with VEGF-B is equally remarkable, closely mirroring TAM’s binding score and forming hydrogen bonds with Thr31 and Leu66. The docking images of PRKC apoptosis WT1 regulator protein (2JK9) with CAP and WA, Indian hedgehog (3N1O) and Suppressor of the fused (4KMH) are represented in Figure 6A–F, respectively. The docking details are listed in Table 4, and their respective 2D and 3D images are depicted in Supplementary Table S7.

**Table 4:** Molecular docking details of key target proteins

PubMed ID of the ligand and pose number	Binding score in kcal/mol	Hydrophilic bonds with distance in Å	Hydrophobic bonds with distance in Å
1. 7QIU—Hedgehog Acyltransferase (HHAT) (Chain A)			
TAM-1(2733525)	−15.3053	Asp141—1.91(H-Bond)	Asp141–2.85 (Salt Bridge) Arg24–6.13 (Pi-Cation)
WA-1(265237)	−14.5232	Phe143—1.87 (H-Bond)	–
2. 4KMH—Suppressor of fused (SUFU) (Chains A, B)			
TAM-1(2733525)	−12.9431	Val(A)104—2.20 (H-Bond)	–
WA-1(265237)	−14.9937	–	Glu448–2.82 (Salt Bridge)
3. 5OMO—Glioma-associated Protein-1 (GLI1) (Chains A, B)			
TAM-1(2733525)	−16.7434	Leu526—2.21 (h-bond)	–
WA-1(265237)	−13.4295	–	Aromatic Bond −2.63 (Ser 521) Aromatic Bond −2.68 (Ser 521) Aromatic Bond—2.48 (Gly 525) Aromatic Bond—2.67 (Asn 481) Aromatic Bond—2.79 (Tyr 476)
4. 4BLD—Glioma-associated Protein-3 (GLI3) (Chains E–H)			
TAM-1(2733525)	−7.4834	–	Aromatic Bond—2.08(Ala 611) Aromatic Bond—2.57(Ser 491) Aromatic Bond—2.22(Ser 491)
WA-1(265237)	−0.3396	Leu 480—2.68(H-Bond)	–
5. 3N1O—Indian Hedgehog (IHH) (Chains A–C)			
TAM-1(2733525)	−15.0456	Glu(B)94—1.69(H-Bond) Phe(B)91—(H-Bond)	–
WA-1(265237)	−17.6299	Asp152—1080(H-Bond)	Asp152–2.80 (Salt Bridge) Hid185–3.95 (Pi-Cation) Hid139–4.97 (Pi–Pi Cation)
6. 3N1G—Desert Hedgehog/Mammalian Hedgehog (DHH) (Chains C, A)			
TAM-1(2733525)	−9.8587	Phe801—2.03 (H-Bond)	Glu800–4.02 (Salt Bridge)
WA-1(265237)	−7.5611	Pro716—1.91(A) (H-Bond)	–
7. 4WPB—Vascular Endothelial Growth Factor (VEGF) and VEGF-A (Chains A, B)			
TAM-1(2733525)	−11.1113	Gly(B)59—1.80 (H-Bond)	Aromatic Bond—2.79 (Glu 30) Aromatic Bond—2.42 (Glu 30) Aromatic Bond—2.29 (Val 33)
WA-1(265237)	−13.9781	Thr31 (A)—2.55 (H-Bond) Leu66 (A)—2.15 (H-Bond)	–
8. 2VWE—Vascular Endothelial Growth Factor-B (Chains A, B)			
TAM-1(2733525)	−7.107		Aromatic Bond—2.56 (Leu 39) Glu96–4.20 (Salt Bridge)
WA-1(265237)	−12.5044	Leu(A)—1.81 (H-Bond)	–
9. 4TTH— Cyclin-Dependent Kinase6 (CDK6) (Chain B)			
TAM-1(2733525)	−18.6402	Asp104—1.97 (H-Bond)	Hid100—4.75 (Pi-Pi Stacking) Asp104—2.90 (Salt Bridge) Aromatic Bond—11.75 (Val 101) Aromatic Bond—2.52 (Gln 103)
WA-1(265237)	−12.9179	Asp104(B)—2.49 (H-Bond)	–
10. 3SHI—Human Matrix Metalloproteinase-1/MMP-1 (Chains A–D)			
TAM-1(2733525)	−14.8875		Glu29—2.90 (Salt Bridge) Aromatic Bond—2.51 (Tyr 210) Aromatic Bond—2.46 (Gly 179) Aromatic Bond—2.59 (Ser 239)
WA-1(265237)	−15.5914	Tyr237—2.39 (H-Bond) Glu219—1.62 (H-Bond)	–
11. 1RTG—Human Matrix Metalloproteinase-2/MMP-2 (Chain A)			
TAM-1(2733525)	−8.795	Glu484(A)—2.16 (H-Bond)	Aromatic Bond—2.33 (Gln 528) Glu484–3.05salt Bridge
WA-1(265237)	−9.2944	Asp 618 (A)—2.10 (H-Bond)	–
12. 5CUH—Human Matrix Metalloproteinase-9/MMP-9 (Chains A, B)			
TAM-1(2733525)	−13.5013	Glu227(A)—1.86 (H-Bond) Tyr248(A)—1.79 (H-Bond)	Zn301–2.46 (Metal Coordination) Zn301–1.05 (Metal Coordination)
WA-1(265237)	−8.4527	Met247(A)—2.04 (H-Bond)	Aromatic Bond—1.97(Gly 186) Zn301–4.36(Pi-Cation)
GENES FROM NETWORK			
13. 1CVU—Prostaglandin Synthase 2 (PTGS2 OR COX2) (Chains A, B)			
CAP-1(60953)	−16.5473	Met522—1.84 (H-Bond)	Tyr385—5.14 (Pi-Pi stacking)
WA-1(265237)	−16.5397	Gly45(A)—1.90 (H-Bond) Gln2327(B)—1.88 (H-Bond)	–
14. 2G01—Mitogen-activated Kinase-8 (MAPK8 OR JNK1) (Chains A, C (AUTH B))			
CAP-1(60953)	−13.2186	Met111(A)—2.07(H-Bond) Asn114(C)—2.04 (H-Bond)	–
WA-1(265237)	−6.5331	Met111(A)—1.70 (H-Bond)	–
15. 2JK9—PRKC Apoptosis WT1 regulator Protein/ PAWR (Chain B)			
CAP-1(60953)	−14.1179	Gly92—1.75 (H-Bond) Arg95—2.16 (H-Bond)	Tyr93—5.32(Pi-Pi tacking)
WA-1(265237)	−14.0674	Asp182—1.59 (H-Bond) Thr35—1.96 (H-Bond)	–
16. 1G5M—B-Cell Leukemia-2 (BCL-2) (Chain A)			
CAP-1(60953)	−20.9354	Asn182—1.95 (H-Bond) Asn182—1.27 (H-Bond) Tyr9—2.57 (H-Bond) Tyr9—1.96 (H-Bond) Arg12—2.13 (H-Bond)	–
WA-1(265237)	−13.3983	Asn11—2.38 (H-Bond) Asp196—2.24 (H-Bond)	–
17. 5VPB—Transcription factor FOSB/JUND (Chains B, D)			
CAP-1(60953)	−5.32	Cys285(D)—1.98 (H-Bond)	–
WA-1(265237)	−2.41	Arg292(B)—2.72 (H-Bond) Cys285(D)—2.19 (H-Bond) Arg288(D)—1.69 (H-Bond) Arg288(D)—2.05 (H-Bond) Arg292(D)—2.25 (H-Bond)	Arg292(D)-4.74(Pi-cation)
18. 4S0S—Human Pregnane X Receptor (Chains A, B)			
CAP-1(60953)	−15.4818	Leu209—1.98 (H-Bond) Hid327—2.70 (H-Bond) Gln285—2.02 (H-Bond)	–
WA-1(265237)	−10.4444	Leu240 (A)—1.62 (H-Bond)	–
19. 4Y5H—Mitogen-activated Kinase 10 (MAPK10), JNK3, JNK3A (Chain A)			
CAP-1(60953)	−14.9197	Met149—2.00 (H-Bond) Met149—1.61 (H-Bond)	–
WA-1(265237)	−8.0208	Met 149—2.17 (H-Bond)	–
20. 5AUT—Death-associated Protein Kinase (DAPK1, DAPK) (Chain A)			
CAP-1(60953)	−15.3683	Glu100—2.10 (H-Bond)	–
WA-1(265237)	−11.7234	Glu143—1.98 (H-Bond)	

### Molecular simulation studies

On thorough analysis of the docking reports, the proteins PRKC apoptosis WT1 regulator (2JK9), vascular endothelial growth factor-B (2VWE), IHH(3N1O), SUFU (4KMH) and vascular endothelial growth factor and VEGFA (4WPB) were proceeded with simulation studies using Desmond software. The simulation interpretations of 2JK9, 2VWE, 3N1O, 4KMH and 4WPB are enlisted below.

#### PRKC apoptosis WT1 regulator protein (2JK9) with CAP and WA

##### Root mean square deviation

**Figure 7:**
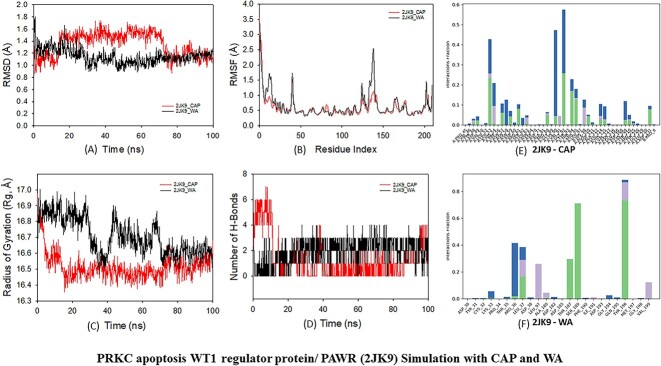

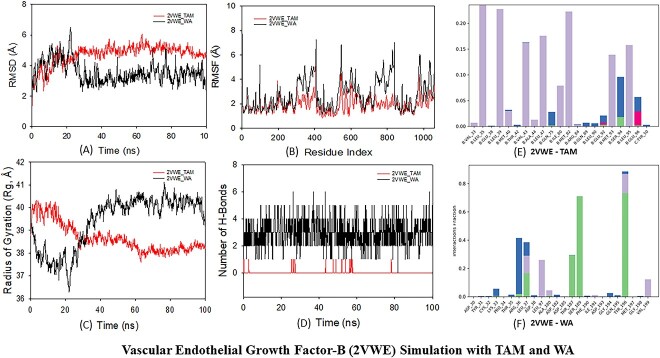

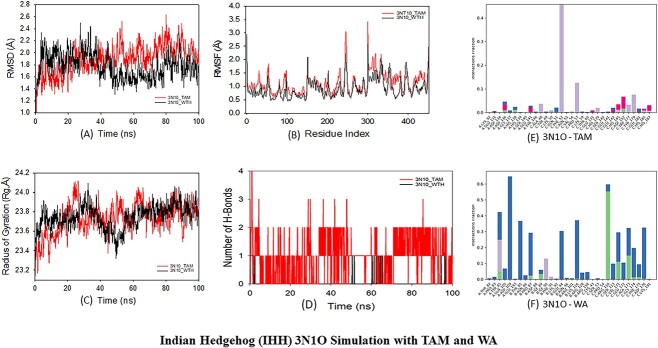

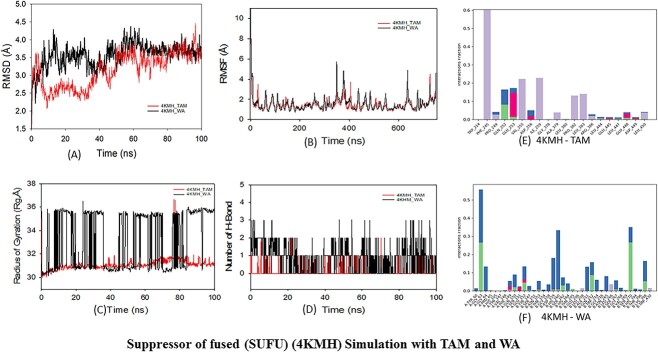

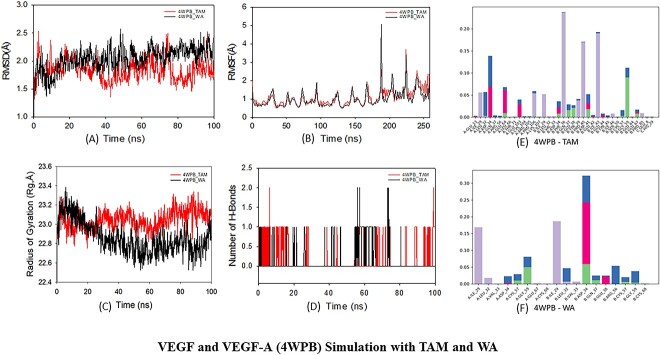
(**A**) MD simulation analysis of 100 ns trajectories of (a) Cα backbone RMSD of 2JK9 bound to ligands CAP. WA. (b) RMSF of Cα backbone of 2JK9 bound to ligands CAP and WA. (c) Cα backbone Rg of 2JK9 bound to ligand CAP. WA. (d) Formation of hydrogen bonds in of 2JK9 bound to ligand TAM and WA. (e) Interactions of CAP. (f) Interactions of WA. (**B**) MD simulation analysis of 100 ns trajectories of (a) Cα backbone RMSD of 2VWE bound to ligand TAM and WA. (b) RMSF of Cα backbone of 2VWE bound to ligands TAM and WA. (c) Cα backbone Rg of 2VWE bound to ligands TAM and WA. (d) Formation of hydrogen bonds in 2VWE bound to ligands TAM and WA. (e) Interactions of TAM. (f) Interactions of WA. (**C**) MD simulation analysis of 100 ns trajectories of (a) Cα backbone RMSD of 3N1O bound to ligands TAM and WA. (b) RMSF of Cα backbone of 3N1O bound to ligands TAM and WA. (c) Cα backbone Rg of 4KMH bound to ligand TAM and WA. (d) Formation of hydrogen bonds in of 3N1O bound to ligand TAM and WA. (e) Interactions of TAM. (f) Interactions of WA. (**D**) MD simulation analysis of 100 ns trajectories of (a) Cα backbone RMSD of 4KMH bound to ligands TAM and WA. (b) RMSF of Cα backbone of 4KMH bound to ligands TAM and WA. (c) Cα backbone Rg of 4KMH bound to ligands TAM and WA. (d) Formation of hydrogen bonds in 4KMH bound to ligand TAM and WA. (e) Interactions of TAM. (f) Interactions of WA. (**E**) MD simulation analysis of 100 ns trajectories of (a) Cα backbone of 4WPB bound to ligands TAM and WA. (b) RMSF of Cα backbone of 4WPB bound to TAM and WA. (c) Cα-backbone Rg of 4WPB bound to ligands TAM and WA. (d) Formation of hydrogen bonds in 4WPB bound to ligands TAM and WA. (e) Interactions of TAM. (f) Interactions of WA.

Molecular dynamic (MD) simulation investigations were executed to ascertain the stability and convergence of the 2JK9 + CAP and 2JK9 + WA complexes. The simulations, spanning a duration of 100 ns, unveiled persistent and stabilized conformations, evident from the RMSD analysis. Specifically, concerning the Cα-backbone of 2JK9 + CAP, deviations were discernible until approximately 70 ns, after which a heightened level of stability was attained until the conclusion of the 100 ns period (Figure 7A, part a). Conversely, the Cα-backbone of 2JK9 + WA exhibited a consistent RMSD of 1.21 Å, indicative of a heightened equilibrium. These outcomes imply that the 2JK9 protein exhibited augmented stability upon interaction with WA, manifesting a superior affinity relative to CAP. The observed RMSD of the protein–ligand complex fell within the 1–3 Å range, which aligns impeccably with established acceptability standards.

**Figure 8:**
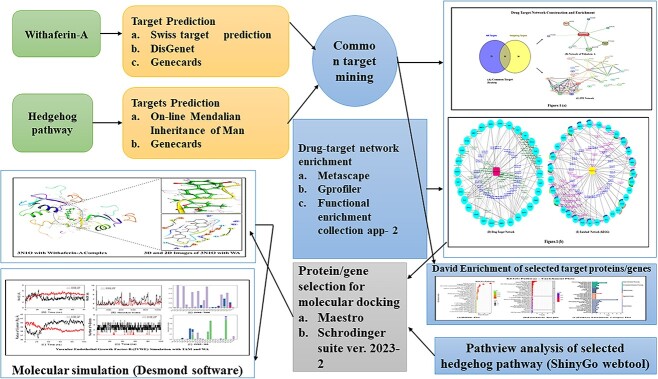
Study-workflow representation.

##### Root mean square fluctuation

The analysis of the RMSF plot illuminated marked oscillations in the 2JK9 protein complexed with WA, particularly within the residues 5–20, 48 and 115–148, underscoring the intrinsic flexibility inherent to these segments (Figure 7A, part b). In stark contrast, minimal fluctuations, predominantly below 1 Å threshold, were noted for 2JK9 + CAP, signifying its comparatively stable demeanor. The oscillations observed in the RMSF plot also denote elevated flexibility within the 2JK9 + WA complex, underscoring the dynamic interplay of the system. It is noted that no amino acids from the catalytic sites (ASP13(A), THR35(A), ARG36(A), LEU37(A), ASP38(A), LEU41(A), LEU42(A), HID54(A), ASP55(A), ASP57(A), LEU97(A), ALA180(A), ASP182(A), ASP185(A), THR187(A), SER189(A), ILE191(A), GLY194(A), TYR196(A), VAL199(A), THR35(A), ARG36(A), LEU37(A), ASP38(A), LEU41(A), LEU97(A), ALA180(A), ASP182(A), ASP185(A), THR187(A), SER189(A), ILE191(A), GLY194(A), TYR196(A), VAL199(A), ASP55(A), ARG57(A), ASP13(A), THY93(A), GLY92(A), ARG95(A), VAL91(A), LYS90(A), GLY96(A), THR94(A)) have been identified in the higher RMSF region (Supplementary Table S8).

##### Radius gyration

The Rg is a metric denoting protein compactness, as evidenced by a reduction in both 2JK9 + CAP and 2JK9 + WA complexes, with Rg values transitioning from 16.9 to 16.66 Å and from 16.9 to 16.54 Å, respectively (Figure 7A, part c).

##### Hydrogen bonds

In the context of 2JK9 + CAP, a limited yet consistent number of hydrogen bonds were recorded on average. Conversely, in the context of 2JK9 + WA, an average of three hydrogen bonds persisted throughout the entire 100 ns simulation (Figure 7A, part d), signifying a more pronounced and enduring interaction with the protein (2JK9). The binding score of WA with protein 2JK9 is −14.0674. There appears to be a correlation between the number of hydrogen bonds and the binding score, as evidenced by the three hydrogen bond interactions that were seen during the 100 ns simulation, suggesting a moderate level of specificity.

##### Molecular mechanics generalized born surface area calculations

The outcomes (Table 5) revealed that ΔGbind in the stability of the simulated complexes was due to ΔGbindCoulomb, ΔGbindvdW and ΔGbindLipo, while ΔGbindCovalent and ΔGbindSolvGB contributed to the instability of the corresponding complexes. Both 2JK9 + CAP and 2JK9 + WA complexes demonstrated notably higher binding free energies, affirming CAP and WA’s strong binding affinity to the protein, efficient interaction and capacity to forge enduring protein–ligand complexes.

**Table 5:** Binding free energies for the 2JK9 + CAP and 2JK9 + WA complexes calculated from MM-GBSA

Energies (kcal/mol)	2JK9 + CAP	2JK9 + WA
ΔG_bind_	−75.72	−77.35
ΔG_bind_Lipo	−9.86	−10.75
ΔG_bind_vdW	−42.60	−45.60
ΔG_bind_Coulomb	−40.86	−39.68
ΔG_bind_H_bond_	−5.05	−5.14
ΔG_bind_SolvGB	23.05	25.56
ΔG_bind_Covalent	2.45	1.72

#### 2VWE with TAM and WA

##### Root mean square deviation

The Cα-backbone RMSD for 2VWE + TAM was found to be 4.1 Å (Figure 7B, part a), whereas the Cα-backbone RMSD of 2VWE + WA exhibited good stability at 2.9 Å. The maintenance of the stable RMSD trajectory over the simulation duration indicated satisfactory convergence and equilibrium of conformations. The protein 2VWE attained notable stability within the complex due to its stronger binding affinity with WA compared to CAP.

##### Root mean square fluctuation

RMSF profiles offered insights into the flexibility of the residues. Heightened fluctuations, seen between 190–200, 500–570 and 700–900 residues at distances up to 4 Å in 2VWE bound to WA (Figure 7B, part b), can be attributed to greater residue flexibility. Similar regions exhibited significant fluctuations in the 2VWE + TAM complex, indicating shared dynamic behavior at these sites. Conversely, a majority of residues displayed diminished fluctuations across the 100 ns simulation, indicating sustained rigidity in amino acid conformations. Hence, RMSF analysis implied the flexible nature of WA during the simulation. With protein 2VWE, preferential binding to WA, as evidenced by lower RMSD, augmented flexibility in certain residues with TAM and compacted protein conformation with both ligands, underscores the intricate interplay between protein and ligand. A low RMSF value with no catalytic site amino acid (Supplementary Table S8) present in the higher RMSF region suggests the presence of a stable and rigid active site. A low RMSF value at the catalytic site indicates high stability in this region. This stability is crucial for catalytic activity, as a stable active site can maintain the proper orientation and environment for substrate binding and catalysis. Overall, low RMSF values in the catalytic site suggest a stable and possibly rigid active site.

##### Radius of gyration

The Rg, a metric of protein compactness, exhibited intriguing alterations. For the 2VWE + WA, it increased marginally from 39.5 to 39.6 Å (Figure 7B, part c).

##### Hydrogen bonds

Examination of hydrogen bond interactions further illuminated complex stability. Specifically, the interaction between 2VWE and TAM yielded an average of one hydrogen bond throughout the simulation, whereas interactions with WA were more robust, averaging four hydrogen bonds over 100 ns (Figure 7B, part d). When compared to TAM (−7.107), protein 2VWE has a substantially higher binding score for WA (−12.5044). The four hydrogen bonds that were seen in the WA simulation point to a high dependence on hydrogen bonding for this context’s improved binding affinity.

##### Molecular mechanics generalized born surface area calculations

The ΔGbind in the stability of the simulated complexes were due to ΔGbind Coulomb, ΔGbindvdW and ΔGbindLipo, while, ΔGbindCovalent and ΔGbindSolvGB contributed to instability. Both 2VWE + TAM, 2VWE + WA exhibited notably higher binding free energies, affirming their robust and stable protein-ligand interactions. The results are listed in Table 6.

**Table 6:** Binding free energies for the 2VWE + TAM and 2VWE + WA complexes calculated from MM-GBSA

Energies (kcal/mol)	2VWE + TAM	2VWE + WA
ΔG_bind_	−43.17	−65.09
ΔG_bind_Lipo	−22.47	−16.43
ΔG_bind_vdW	−32.59	−48.83
ΔG_bind_Coulomb	−16.35	−24.88
ΔG_bind_H_bond_	−0.09	−2.10
ΔG_bind_SolvGB	6.59	24.55
ΔG_bind_Covalent	2.22	2.61

#### Indian hedgehog (3N1O) with TAM and WA

##### Root mean square deviation

The Cα-backbone RMSD of 3N1O + TAM displayed initial fluctuations within the first 50 ns, followed by enhanced stability with an average value below 1.8 Å (Figure 7C, part a). Similarly, the Cα-backbone RMSD of 3N1O + WA demonstrated consistent stability at 1.6 Å.

##### Root mean square fluctuation

The RMSF examinations revealed notable spikes in fluctuation at specific residues 150–200, 200, 200–220 and 300–390 for both complexes (Figure 7C, part b). However, the majority of residues exhibited minimal fluctuations, indicating predominantly rigid amino acid conformations during the simulation. The MD simulations elucidated stable and convergent behaviors in the 3N1O + TAM and 3N1O + WA complexes over the 100 ns simulation period. These complexes demonstrated rigid protein structures with localized flexibility in ligand-binding regions, as evidenced by RMSF analysis. It is noted that none of the catalytic amino acid residues (GLU68(A), ILE71(A), ARG77(A), PHE78(A), LYS79(A), GLU80(A), LEU81(A), THR82(A), PRO83(A), TYR85(A), ASN86(A), PRO87(A), ILE89(A), ILE90(A), PHE91(A), ARG101(A), LEU102(A), THR104(A), GLN105(A), ARG106(A), LYS108(A), ASP109(A), ARG110(A), ASN112(A), THR130(A), GLU131(A), HID139(A), SER140(A), HIS149(A), GLU147(A), ARG149(A), ASP152(A), TRP177(A), TYR179(A), GLU181(A), HIS185(A), HID185(A), LYS191(A), SER192(A), GLU193(A), TYR85(B), PRO87(B), ASP88(B), ILE89(B), ILE90(B), PHE91(B), GLU94(B), LYS43(C), LEU44(C), VAL45(C), SER53(C), PRO54(C), VAL56(C), LEU61(C), GLY62(C), GLN105(C), ARG106(C), ASP109(C), ARG110(C), SER113(C), SER117(C), ASN120(C), GLN121(C), ARG168(C), LEU169(C), VAL171(C), GLU172(C), ALA173(C), GLY174(C), PHE175(C), ASP176(C), VAL178(C), LYS191(C), SER192(C), GLU193(C)) were found in the RMSF region (Supplementary Table S8). A low RMSF value in the catalytic site indicates high stability in this region. This stability is crucial for the catalytic activity, as a stable active site can maintain the proper orientation and environment for the substrate binding and catalysis.

##### Radius of gyration

The Rg analysis revealed compactness with a stable Rg values for both complexes. Specifically, the 3N1O + WA complex maintained an Rg between 23.4 and 23.6 Å, while the 3N1O + TAM complex displayed fluctuating Rg values ranging between 23.6 and 23.8 Å (Figure 7C, part c). These consistent Rg values suggest a compact protein orientation in the ligand-bound state.

##### Hydrogen bonds

The complex 3N1O + TAM exhibited two hydrogen bonds, while the 3N1O + WA complex displayed single sporadic hydrogen bonding observed constant throughout the 100 ns simulation period. This indicates the robust interaction and stability of the complex (Figure 7C, part d). For protein 3N1O, despite having single hydrogen bond, WA shows a substantially higher binding score (−17.6299) compared to TAM (−15.0456). This suggests that the hydrogen bonding, in conjunction with other types of bonds, played a substantial role in influencing the overall binding affinity of WA in protein 3N1O.

##### Molecular mechanics generalized born surface area calculations

The results disclosed that the stability of the simulated 3N1O + TAM complex stems primarily from ΔGbindvdW, ΔGbindLipo and ΔGbindSolvGB, while ΔGbindCoulomb and ΔGbindCovalent contribute to its instability. Conversely, in the 3N1O + WA complex, ΔGbindvdW, ΔGbindLipo and ΔGbindCoulomb underpin stability, while ΔGbindSolvGB and ΔGbindCovalent contribute to instability. Both complexes exhibit notably higher binding free energies, with WA showcasing remarkably strong bindings (Table 7).

**Table 7:** Binding free energies for the 3N1O + TAM and 3N1O + WA complexes calculated from MM-GBSA

Energies (kcal/mol)	3N1O + TAM	3N1O + WA
ΔG_bind_	−18.56	−45.81
ΔG_bind_Lipo	−10.64	−14.55
ΔG_bind_vdW	−15.63	−53.98
ΔG_bind_Coulomb	13.59	−12.28
ΔG_bind_H_bond_	−0.005	−0.65
ΔG_bind_SolvGB	−6.92	27.68
ΔG_bind_Covalent	1.09	7.97

#### SUFU (4KMH) with TAM and WA

##### Root mean square deviation

The RMSD of Cα-backbone of 4KMH + TAM demonstrated initial fluctuations up to 50 ns, followed by heightened stability up to 100 ns, with an average below 3.5 Å, while the RMSD of the Cα-backbone of 4KMH + WA remained constant at 3.5 Å (Figure 7D, part a). The protein 4KMH retained stability within the complex, owing to its stronger affinity with WA and TAM.

##### Root mean square fluctuations

Analysis of RMSFs exhibited pronounced spikes of fluctuations at residues 380, 390 and 650 in the 4KMH protein bound to TAM and WA (Figure 7D, part b). These fluctuations likely arise from the enhanced flexibility of these residues. Conversely, the majority of residues demonstrated reduced fluctuations throughout the 100 ns simulation, indicating a prevailing rigidity in amino acid conformations during this time frame. Notably, the regions with higher RMSF values did not include any of the catalytic site amino acids (VAL104(A), ASP403(A), MET404(A), ALA405(A), ILE406(A), THR407(A), PHE408(A), VAL409(A), SER410(A), THR411(A), GLY412(A), VAL413(A), GLU414(A), GLY415(A), ALA416(A), PHE417(A), ALA418(A), THR419(A), GLU420(A), GLU421(A), HIS422(A), PRO423(A), TYR424(A), ALA425(A), ALA426(A), HIS427(A), GLY428(A), PRO429(A), TRP430(A), LEU431(A), GLN432(A), ILE433(A), LEU434(A), LEU435(A), THR436(A), GLU437(A), GLU438(A), PHE439(A), VAL440(A), GLU441(A), LYS442(A), MET443(A), LEU444(A), GLU445(A), ASP446(A), LEU447(A), GLU448(A), ASP449(A), LEU450(A), GLU455(A), PHE456(A), LYS457(A), LEU458(A), PRO459(A), LYS460(A), GLU461(A), TYR462(A), SER463(A), TRP464(A), PRO465(A), GLU466(A), LYS467(A), LYS468(A), LEU469(A), LYS470(A), VAL471(A), SER472(A), ILE473(A), LEU474(A), PRO475(A), ASP476(A), VAL477(A), VAL478(A), PHE479(A), ASP480(A), SER481(A), PRO20(B), THR21(B), ALA22(B), PRO23(B), PRO24(B), ALA25(B), PHE26(B), ALA27(B), SER28(B), LEU29(B), PHE30(B), PRO31(B), PRO32(B), GLY33(B), LEU34(B), HIS35(B), ALA36(B), ILE37(B), TYR38(B), GLY39(B), GLU40(B), CYS41(B), ARG42(B), ARG43(B), LEU44(B)), as detailed in Supplementary Table S8. Moreover, a reduction in fluctuation was observed throughout the 100 ns molecular simulation, suggesting the overall stability of the complexes.

##### Radius of gyration

The 4KMH + TAM complex, specifically the Cα-backbone, exhibited a consistent Rg range of 30–31 Å, whereas the 4KMH + WA complex displayed fluctuating Rg values, ranging from 30 to 36 Å (Figure 7D, part c). The Rg pattern suggests a highly compact protein orientation in the ligand-bound state.

##### Hydrogen bonds

The count of hydrogen bonds formed between 4KMH and TAM is two, while a single hydrogen bond between WA and 4KMH remained constant throughout the 100 ns simulation (Figure 7D, part d). In protein 4KMH, the ligand TAM exhibits a significantly lower binding score of −12.9431, accompanied by the involvement of two hydrogen bonds, in contrast to WA with a higher binding score of −14.9937 and single hydrogen bond. This suggests that in conjunction with hydrogen bonding, other types of interactions play a substantial role in determining the overall affinity.

##### Mechanics generalized born surface area calculations

The mechanics generalized born surface area (MM-GBSA) calculations indicate the stability of the simulated complexes, was primarily influenced by ΔGbindvdW, ΔGbindLipo and ΔGbindCoulomb, while ΔGbindCovalent and ΔGbindSolvGB contributed to the instability of these complexes. Hydrogen bonding contributes to binding in both complexes, slightly favoring 4KMH + WA (Table 8).

**Table 8:** Binding free energy components for the 4KMH + TAM and 4KMH + WA complexes calculated from MM-GBSA

Energies (kcal/mol)	4KMH + TAM	4KMH + WA
ΔG_bind_	−168.87	−96.37
ΔG_bind_Lipo	−33.51	−33.93
ΔG_bind_vdW	−122.27	−123.22
ΔG_bind_Coulomb	−28.95	29.69
ΔG_bind_H_bond_	−8.72	−11.14
ΔG_bind_SolvGB	22.01	88.43
ΔG_bind_Covalent	4.12	6.48

#### VEGF and VEGF-A (4WPB) with TAM and WA

##### Root mean square deviation

The Cα-backbone RMSD of 4WPB + TAM exhibited initial fluctuations up to 40 ns, followed by improved stability up to 100 ns, maintaining an average value below 2 Å. Conversely, the Cα-backbone RMSD of 4WPB + WA remained consistently stable at 2.2 Å. The consistent RMSD plot during the simulation signified robust convergence and stable conformations of WA with the 4WPB protein (Figure 7E, part a).

##### Root mean square fluctuation

The RMSF plot unveiled substantial spikes of fluctuation in the 4WPB protein bound to TAM and WA, observed at residues 190, 210 and 225 (Figure 7E, part b). These fluctuations could be attributed to the heightened flexibility of these residues. Conversely, the majority of the residues demonstrated diminished fluctuations throughout the entire 100 ns simulation, suggesting a prevailing rigidity in amino acid conformations during this period. Consequently, the RMSF plots propose a rigid protein structure in the presence of TAM, whereas slight flexibility is observed with WA. Despite observing fluctuations, none of the amino acids within the catalytic site (MET94(B), SER95(B), PHE96(B), LEU97(B), GLN98(B), HIS99(B), ASN100(B), LYS101(B), CYS102(B), GLU103(B), CYS104(B), ARG105(B), PRO106(B), GLU8(C), CYS10(C), ASN11(C), ARG13(C), ALA14(C), ILE15(C), GLU16(C), AIB17(C), ALA18(C), LEU19(C), ASP20(C), PRO21(C), ASN22(C)) were identified in the higher RMSF region (Supplementary Table S8).

##### Radius gyration

In the 4WPB + TAM complex, the Cα-backbone, exhibited fluctuating Rg from 23 to 23.4 Å, while the 4WPB + WA complex displayed a consistently stable Rg range from 22.9 to 23 Å. The lowered Rg signifies a highly compact protein orientation in the ligand-bound state (Figure 7E, part c).

##### Hydrogen bonds

The 4WPB complex formed a single hydrogen bond with both TAM and WA, which remained constant throughout the 100 ns simulation (Figure 7E, part d). In the case of protein 4WPB, the ligand WA demonstrates a notably higher binding score of −13.9781 compared to TAM, which has a binding score of −11.1113. This substantial difference in binding scores suggests a stronger affinity of WA for protein 4WPB. The observation of a hydrogen bond in the WA simulation further underscores the significance of hydrogen bonding in facilitating the interaction between WA and protein 4WPB.

##### M‌M-GBSA calculations

The results (Table 9) indicate that the stability of the simulated complexes was primarily influenced by ΔGbindvdW, ΔGbindLipo and ΔGbindSolvGB, while ΔGbindCovalent and ΔGbindCoulomb contributed to the instability of these complexes. Notably, the 4WPB + TAM and 4WPB + WA complexes exhibited significantly higher binding energies.

**Table 9:** Binding free energy components for the 4WPB + TAM and 4WPB + WA calculated from MM-GBSA

Energies (kcal/mol)	4WPB + TAM	4WPB + WA
ΔG_bind_	−106.44	−85.47
ΔG_bind_Lipo	−30.66	−22.11
ΔG_bind_vdW	−76.14	−75.24
ΔG_bind_Coulomb	24.36	29.69
ΔG_bind_H_bond_	−4.70	−4.95
ΔG_bind_SolvGB	−22.20	−14.64
ΔG_bind_Covalent	6.49	2.06

In accordance with simulation reports, low RMSF values in catalytic sites indicate a stable active site, vital for substrate binding and catalysis. This stability, crucial for proper orientation, suggests a specific and possibly limited substrate range. High RMSF values on the active site may reflect conformational changes post-ligand binding, forming stable loops for tight ligand accommodation. Overall, low RMSF implies a rigid active site, influencing substrate specificity and catalytic mechanism. Further, WA consistently demonstrated strong binding affinities comparable to or surpassing those of standard ligands.

### Prediction of ADME and drug-likeness properties of WA

The drug WA complied with standards for drug-like properties and molecular attributes, and its hydrogen bond donors, acceptors, rotatable bonds, heteroatoms and heavy atoms were in acceptable proportions. The optical surface area, which complied with the Veber criteria, was 93.20. Good absorption, high plasma protein binding (85.585%), a sizable volume of distribution (0.741) and blood–brain barrier penetration were all suggested by pharmacokinetics data. Cytochrome 2C19 and 3A4 were used in the biotransformation process, and para-glycoprotein inhibition was noted. With a few exceptions, bioavailability (0.55) complied with drug-likeness requirements and fell within medium range (T½ ≤ 3). Overall drug-likeness properties adhered to established rules, except for certain deviations [51, 55, 56] enumerated in Supplementary Table S6.

### Toxicity screening and acute toxicity prediction

The phyto-constituent WA exhibited non-toxic characteristics in several organs, including the kidney, liver, skin and eyes, while displaying certain indicators of toxicity within the respiratory system. The compound WA does not raise concerns regarding acute toxicity, implying a lack of immediate harm at typical exposure levels. The relatively low score on the non-genotoxic carcinogenicity rule suggests a reduced risk of causing cancer through direct DNA damage, while the low score on the non-biodegradable rule, indicating lower level impacts of environmental persistence concern [15, 52]. Furthermore, toxicity studies were conducted on WA, which revealed a no observable effect level (NOEL) dose of 500 mg. Significantly, this compound exhibited safety even at doses exceeding 2000 mg [56]. The toxicity profile is categorized and listed in Supplementary Table S7.

## CONCLUSION

This computational study extensively explored the potential of WA as an anti-therapeutic agent against breast cancer. Specifically, the exact mechanism of WA was unveiled at the stem-cell level. The research findings obtained from the simulation studies on IHH proteins (3N1O) and SUFU (4KMH) further support stem-cell modulation. These meticulous investigations were supported by enrichment analysis (p 0.025).

Docking and dynamic simulations demonstrated the effectiveness of WA in encaging Hh proteins, curbing VEGF and modulating the PRKC apoptosis WT1 regulator protein, all of which are associated with cancer-suppression mechanistic pathways. An appreciative ADMET profile made WA a valid candidate. The research delves into WA’s leverage on the Hh pathway and stem-cell dynamics, revealing its anti-breast cancer credibility. WA’s feasibility is affirmed by binding affinity, docking stability and simulations. Translation into breast cancer necessitates comprehensive experimental validation and preclinical assessment.

**Key Points**

- Withaferin-A’s (WA’s) breast cancer role identification.
- *In silico* analysis of WA’s intrinsic pathway proteins.
- *In silico* analysis of W-A’s impact on hedgehog pathway proteins.
- Network pharmacology and enrichment analysis.
- Computational analysis using molecular docking and simulation.

## Supplementary Material

Supplementary_Tables_for_journal_bbae032

## Data Availability

For access to any research-related data, kindly reach out to the corresponding author.
