# Supplementary material for: Integrating network pharmacology and in silico analysis deciphers Withaferin-A’s anti-breast cancer potential via hedgehog pathway and target network interplay
Source: Brief Bioinform. 2024 Mar 5;25(2):bbae032. doi: 10.1093/bib/bbae032 (PMC10917074; doi:10.1093/bib/bbae032)
Supplement: Supplementary_Tables_for_journal_bbae032 [file supplementary_tables_for_journal_bbae032.docx]

**Supplementary table S1.** Selected Withaferin targets (Dataset.1)

| **Sr. No** | From | To | Species | David Gene Name |
| --- | --- | --- | --- | --- |
|  | ATF2 | 1386 | Homo sapiens | activating transcription factor 2(ATF2) |
|  | GSK3B | 2932 | Homo sapiens | glycogen synthase kinase 3 beta (GSK3B) |
|  | GSK3A | 2931 | Homo sapiens | glycogen synthase kinase 3 alpha (GSK3A) |
|  | LRRK2 | 120892 | Homo sapiens | leucine rich repeat kinase 2(LRRK2) |
|  | IHH | 3549 | Homo sapiens | Indian hedgehog signaling molecule (IHH) |
|  | ATP2A1 | 487 | Homo sapiens | ATPase sarcoplasmic/endoplasmic reticulum Ca2+ transporting 1(ATP2A1) |
|  | PCSK7 | 9159 | Homo sapiens | proprotein convertase subtilisin/kexin type 7(PCSK7) |
|  | GLI1 | 2735 | Homo sapiens | GLI family zinc finger 1(GLI1) |
|  | NR3C1 | 2908 | Homo sapiens | nuclear receptor subfamily 3 group C member 1(NR3C1) |
|  | GLI3 | 2737 | Homo sapiens | GLI family zinc finger 3(GLI3) |
|  | GLI2 | 2736 | Homo sapiens | GLI family zinc finger 2(GLI2) |
|  | NR3C2 | 4306 | Homo sapiens | nuclear receptor subfamily 3 group C member 2(NR3C2) |
|  | IKBKB | 3551 | Homo sapiens | inhibitor of nuclear factor kappa B kinase subunit beta (IKBKB) |
|  | SHH | 6469 | Homo sapiens | sonic hedgehog signaling molecule (SHH) |
|  | HHAT | 55733 | Homo sapiens | hedgehog acyltransferase (HHAT) |
|  | CHEK2 | 11200 | Homo sapiens | checkpoint kinase 2(CHEK2) |
|  | SUFU | 51684 | Homo sapiens | SUFU negative regulator of hedgehog signaling (SUFU) |
|  | ADORA1 | 134 | Homo sapiens | adenosine A1 receptor (ADORA1) |
|  | UPP2 | 151531 | Homo sapiens | uridine phosphorylase 2(UPP2) |
|  | JAK3 | 3718 | Homo sapiens | Janus kinase 3(JAK3) |
|  | EPHB4 | 2050 | Homo sapiens | EPH receptor B4(EPHB4) |
|  | EIF2A | 83939 | Homo sapiens | eukaryotic translation initiation factor 2A(EIF2A) |
|  | PRKCG | 5582 | Homo sapiens | protein kinase C gamma (PRKCG) |
|  | MAP2K3 | 5606 | Homo sapiens | mitogen-activated protein kinase 3(MAP2K3) |
|  | MAP2K4 | 6416 | Homo sapiens | mitogen-activated protein kinase 4(MAP2K4) |
|  | PRKCH | 5583 | Homo sapiens | protein kinase C eta (PRKCH) |
|  | MMP1 | 4312 | Homo sapiens | matrix metallopeptidase 1(MMP1) |
|  | PRKCB | 5579 | Homo sapiens | protein kinase C beta (PRKCB) |
|  | FBXW11 | 23291 | Homo sapiens | F-box and WD repeat domain containing 11(FBXW11) |
|  | DAPK1 | 1612 | Homo sapiens | death associated protein kinase 1(DAPK1) |
|  | MMP2 | 4313 | Homo sapiens | matrix metallopeptidase 2(MMP2) |
|  | PRKCE | 5581 | Homo sapiens | protein kinase C epsilon (PRKCE) |
|  | PDE4D | 5144 | Homo sapiens | phosphodiesterase 4D(PDE4D) |
|  | PRKCD | 5580 | Homo sapiens | protein kinase C delta (PRKCD) |
|  | DAPK3 | 1613 | Homo sapiens | death associated protein kinase 3(DAPK3) |
|  | PAWR | 5074 | Homo sapiens | pro-apoptotic WT1 regulator (PAWR) |
|  | PRPF4B | 8899 | Homo sapiens | pre-mRNA processing factor 4B(PRPF4B) |
|  | PRKCA | 5578 | Homo sapiens | protein kinase C alpha (PRKCA) |
|  | OXSR1 | 9943 | Homo sapiens | oxidative stress responsive kinase 1(OXSR1) |
|  | MMP9 | 4318 | Homo sapiens | matrix metallopeptidase 9(MMP9) |
|  | ERN1 | 2081 | Homo sapiens | endoplasmic reticulum to nucleus signaling 1(ERN1) |
|  | AR | 367 | Homo sapiens | androgen receptor (AR) |
|  | MMP11 | 4320 | Homo sapiens | matrix metallopeptidase 11(MMP11) |
|  | SMO | 6608 | Homo sapiens | smoothened, frizzled class receptor (SMO) |
|  | PRKD3 | 23683 | Homo sapiens | protein kinase D3(PRKD3) |
|  | PRKCQ | 5588 | Homo sapiens | protein kinase C theta (PRKCQ) |
|  | PGR | 5241 | Homo sapiens | progesterone receptor (PGR) |
|  | TP53 | 7157 | Homo sapiens | tumor protein p53(TP53) |
|  | CSNK1G1 | 53944 | Homo sapiens | casein kinase 1 gamma 1(CSNK1G1) |
|  | PRKAA2 | 5563 | Homo sapiens | protein kinase AMP-activated catalytic subunit alpha 2(PRKAA2) |
|  | PRKDC | 5591 | Homo sapiens | protein kinase, DNA-activated, catalytic subunit (PRKDC) |
|  | SRC | 6714 | Homo sapiens | SRC proto-oncogene, non-receptor tyrosine kinase (SRC) |
|  | DHH | 50846 | Homo sapiens | desert hedgehog signaling molecule (DHH) |
|  | NR1I2 | 8856 | Homo sapiens | nuclear receptor subfamily 1 group I member 2(NR1I2) |
|  | STK39 | 27347 | Homo sapiens | serine/threonine kinase 39(STK39) |
|  | PTGS2 | 5743 | Homo sapiens | prostaglandin-endoperoxide synthase 2(PTGS2) |
|  | CYP19A1 | 1588 | Homo sapiens | cytochrome P450 family 19 subfamily A member 1(CYP19A1) |
|  | MAPK8 | 5599 | Homo sapiens | mitogen-activated protein kinase 8(MAPK8) |
|  | CXCR1 | 3577 | Homo sapiens | C-X-C motif chemokine receptor 1(CXCR1) |
|  | GRK7 | 131890 | Homo sapiens | G protein-coupled receptor kinase 7(GRK7) |
|  | STK38 | 11329 | Homo sapiens | serine/threonine kinase 38(STK38) |
|  | ABL1 | 25 | Homo sapiens | ABL proto-oncogene 1, non-receptor tyrosine kinase (ABL1) |
|  | MAPK1 | 5594 | Homo sapiens | mitogen-activated protein kinase 1(MAPK1) |
|  | MAP2K7 | 5609 | Homo sapiens | mitogen-activated protein kinase 7(MAP2K7) |
|  | MAP2K6 | 5608 | Homo sapiens | mitogen-activated protein kinase 6(MAP2K6) |
|  | BRD4 | 23476 | Homo sapiens | bromodomain containing 4(BRD4) |
|  | HIPK4 | 147746 | Homo sapiens | homeodomain interacting protein kinase 4(HIPK4) |
|  | CREBBP | 1387 | Homo sapiens | CREB binding protein (CREBBP) |
|  | XBP1 | 7494 | Homo sapiens | X-box binding protein 1(XBP1) |
|  | JUND | 3727 | Homo sapiens | JunD proto-oncogene, AP-1 transcription factor subunit (JUND) |
|  | PTCH1 | 5727 | Homo sapiens | patched 1(PTCH1) |
|  | PTCH2 | 8643 | Homo sapiens | patched 2(PTCH2) |
|  | VEGFB | 7423 | Homo sapiens | vascular endothelial growth factor B(VEGFB) |
|  | PTK2 | 5747 | Homo sapiens | protein tyrosine kinase 2(PTK2) |
|  | VEGFA | 7422 | Homo sapiens | vascular endothelial growth factor A(VEGFA) |
|  | P2RX7 | 5027 | Homo sapiens | purinergic receptor P2X 7(P2RX7) |
|  | IL4 | 3565 | Homo sapiens | interleukin 4(IL4) |
|  | MAPK10 | 5602 | Homo sapiens | mitogen-activated protein kinase 10(MAPK10) |
|  | MAP3K15 | 389840 | Homo sapiens | mitogen-activated protein kinase 15(MAP3K15) |
|  | SBK1 | 388228 | Homo sapiens | SH3 domain binding kinase 1(SBK1) |
|  | PDE10A | 10846 | Homo sapiens | phosphodiesterase 10A(PDE10A) |
|  | CDK4 | 1019 | Homo sapiens | cyclin dependent kinase 4(CDK4) |
|  | BCL2 | 596 | Homo sapiens | BCL2 apoptosis regulator (BCL2) |
|  | MDM2 | 4193 | Homo sapiens | MDM2 proto-oncogene (MDM2) |
|  | FOSB | 2354 | Homo sapiens | FosB proto-oncogene, AP-1 transcription factor subunit (FOSB) |
|  | MAP3K13 | 9175 | Homo sapiens | mitogen-activated protein kinase 13(MAP3K13) |
|  | ITM2B | 9445 | Homo sapiens | integral membrane protein 2B(ITM2B) |
|  | BCL2L1 | 598 | Homo sapiens | BCL2 like 1(BCL2L1) |
|  | From | To | Species | David Gene Name |
|  | ATF2 | 1386 | Homo sapiens | activating transcription factor 2(ATF2) |
|  | GSK3B | 2932 | Homo sapiens | glycogen synthase kinase 3 beta (GSK3B) |
|  | GSK3A | 2931 | Homo sapiens | glycogen synthase kinase 3 alpha (GSK3A) |
|  | LRRK2 | 120892 | Homo sapiens | leucine rich repeat kinase 2(LRRK2) |
|  | IHH | 3549 | Homo sapiens | Indian hedgehog signaling molecule (IHH) |
|  | ATP2A1 | 487 | Homo sapiens | ATPase sarcoplasmic/endoplasmic reticulum Ca2+ transporting 1(ATP2A1) |
|  | PCSK7 | 9159 | Homo sapiens | proprotein convertase subtilisin/kexin type 7(PCSK7) |
|  | GLI1 | 2735 | Homo sapiens | GLI family zinc finger 1(GLI1) |
|  | NR3C1 | 2908 | Homo sapiens | nuclear receptor subfamily 3 group C member 1(NR3C1) |
|  | GLI3 | 2737 | Homo sapiens | GLI family zinc finger 3(GLI3) |
|  | GLI2 | 2736 | Homo sapiens | GLI family zinc finger 2(GLI2) |
|  | NR3C2 | 4306 | Homo sapiens | nuclear receptor subfamily 3 group C member 2(NR3C2) |
|  | IKBKB | 3551 | Homo sapiens | inhibitor of nuclear factor kappa B kinase subunit beta (IKBKB) |
|  | SHH | 6469 | Homo sapiens | sonic hedgehog signaling molecule (SHH) |
|  | HHAT | 55733 | Homo sapiens | hedgehog acyltransferase (HHAT) |
|  | CHEK2 | 11200 | Homo sapiens | checkpoint kinase 2(CHEK2) |

**Supplementary table S2.** Selected hedgehog proteins (Dataset.2)

| **Symbol** | **Description** | **Category** | **Uniprot ID** | **Score** |
| --- | --- | --- | --- | --- |
| SHH | Sonic Hedgehog Signaling Molecule | Protein Coding | Q15465 | 93.27 |
| TP53 | Tumor Protein P53 | Protein Coding | P04637 | 76.59 |
| IHH | Indian Hedgehog Signaling Molecule | Protein Coding | Q14623 | 62.26 |
| PTCH1 | Patched 1 | Protein Coding | Q13635 | 56.53 |
| APC | APC Regulator of WNT Signaling Pathway | Protein Coding | P25054 | 52.81 |
| DHH | Desert Hedgehog Signaling Molecule | Protein Coding | O43323 | 49.55 |
| IL6 | Interleukin 6 | Protein Coding | P05231 | 48.24 |
| MAPK1 | Mitogen-Activated Protein Kinase 1 | Protein Coding | P28482 | 48.23 |
| TGFB1 | Transforming Growth Factor Beta 1 | Protein Coding | P01137 | 48.17 |
| GLI1 | GLI Family Zinc Finger 1 | Protein Coding | P08151 | 47.93 |
| EGFR | Epidermal Growth Factor Receptor | Protein Coding | P00533 | 47.39 |
| H19 | H19 Imprinted Maternally Expressed Transcript | RNA Gene |  | 47.09 |
| SUFU | SUFU Negative Regulator of Hedgehog Signaling | Protein Coding | Q9UMX1 | 46.38 |
| PTEN | Phosphatase And Tensin Homolog | Protein Coding | P60484 | 46.28 |
| VEGFA | Vascular Endothelial Growth Factor A | Protein Coding | P15692 | 41.9 |
| HHIP | Hedgehog Interacting Protein | Protein Coding | Q96QV1 | 41.86 |
| SMO | Smoothened, Frizzled Class Receptor | Protein Coding | Q99835 | 41.58 |
| CDKN2A | Cyclin Dependent Kinase Inhibitor 2A | Protein Coding | Q8N726 | 41.54 |
| NFKB1 | Nuclear Factor Kappa B Subunit 1 | Protein Coding | P19838 | 40.71 |
| IL1B | Interleukin 1 Beta | Protein Coding | P01584 | 40.38 |
| ESR1 | Estrogen Receptor 1 | Protein Coding | P03372 | 40.2 |
| CCND1 | Cyclin D1 | Protein Coding | P24385 | 40.12 |
| BCL2 | BCL2 Apoptosis Regulator | Protein Coding | P10415 | 39.86 |
| GLI2 | GLI Family Zinc Finger 2 | Protein Coding | P10070 | 35.9 |
| MAPK8 | Mitogen-Activated Protein Kinase 8 | Protein Coding | P45983 | 34.97 |
| FOS | Fos Proto-Oncogene, AP-1 Transcription Factor Subunit | Protein Coding | P01100 | 34.87 |
| GLI3 | GLI Family Zinc Finger 3 | Protein Coding | P10071 | 33.35 |
| CDKN1A | Cyclin Dependent Kinase Inhibitor 1A | Protein Coding | P38936 | 32.98 |
| HHAT | Hedgehog Acyltransferase | Protein Coding | Q5VTY9 | 30.97 |
| EGF | Epidermal Growth Factor | Protein Coding | P01133 | 30.75 |
| NF1 | Neurofibromin 1 | Protein Coding | P21359 | 30.45 |
| FAS | Fas Cell Surface Death Receptor | Protein Coding | P25445 | 30.37 |
| MDM2 | MDM2 Proto-Oncogene | Protein Coding | Q00987 | 30.18 |
| FGFR2 | Fibroblast Growth Factor Receptor 2 | Protein Coding | P21802 | 30.08 |
| MBL2 | Mannose Binding Lectin 2 | Protein Coding | P11226 | 30.01 |
| GNAS | GNAS Complex Locus | Protein Coding | P84996 | 29.95 |
| NRAS | NRAS Proto-Oncogene, GTPase | Protein Coding | P01111 | 29.93 |
| CREB1 | CAMP Responsive Element Binding Protein 1 | Protein Coding | P16220 | 29.83 |
| STAT3 | Signal Transducer and Activator of Transcription 3 | Protein Coding | P40763 | 45.06 |
| JUN | Jun Proto-Oncogene, AP-1 Transcription Factor Subunit | Protein Coding | P05412 | 44.5 |
| MYC | MYC Proto-Oncogene, BHLH Transcription Factor | Protein Coding | P01106 | 43.87 |
| BRCA1 | BRCA1 DNA Repair Associated | Protein Coding | P38398 | 43.65 |
| BRCA2 | BRCA2 DNA Repair Associated | Protein Coding | P51587 | 43.42 |
| BRAF | B-Raf Proto-Oncogene, Serine/Threonine Kinase | Protein Coding | P15056 | 42.18 |
| HHIP | Hedgehog Interacting Protein | Protein Coding | Q96QV1 | 41.86 |
| ATM | ATM Serine/Threonine Kinase | Protein Coding | Q13315 | 41.58 |
| KRAS | KRAS Proto-Oncogene, GTPase | Protein Coding | P01116 | 41.58 |
| SMO | Smoothened, Frizzled Class Receptor | Protein Coding | Q99835 | 41.58 |
| APOE | Apolipoprotein E | Protein Coding | P02649 | 38.93 |
| JAK2 | Janus Kinase 2 | Protein Coding | O60674 | 32.02 |
| FGFR1 | Fibroblast Growth Factor Receptor 1 | Protein Coding | P11362 | 31.85 |
| ERBB2 | Erb-B2 Receptor Tyrosine Kinase 2 | Protein Coding | P04626 | 38.4 |
| PPARG | Peroxisome Proliferator Activated Receptor Gamma | Protein Coding | P37231 | 37.85 |
| MAP2K1 | Mitogen-Activated Protein Kinase 1 | Protein Coding | Q02750 | 37.84 |
| AR | Androgen Receptor | Protein Coding | P10275 | 37.59 |
| HIF1A | Hypoxia Inducible Factor 1 Subunit Alpha | Protein Coding | Q16665 | 37.37 |
| MAPK14 | Mitogen-Activated Protein Kinase 14 | Protein Coding | Q16539 | 36.78 |
| SMAD4 | SMAD Family Member 4 | Protein Coding | Q13485 | 36.55 |
| IL10 | Interleukin 10 | Protein Coding | P22301 | 36.24 |
| CREBBP | CREB Binding Protein | Protein Coding | Q92793 | 36.21 |
| MAPK3 | Mitogen-Activated Protein Kinase 3 | Protein Coding | P27361 | 34.02 |
| CASP3 | Caspase 3 | Protein Coding | P42574 | 34.02 |
| INS | Insulin | Protein Coding | P01308 | 33.93 |
| GSK3B | Glycogen Synthase Kinase 3 Beta | Protein Coding | P49841 | 33.71 |

**Supplementary table S3.** Common target proteins selected for network pharmacology

| **Sr. No** | **Gene/Protein** | **Description** | **Category** | **Uniprot ID** | **GIFTS** | **GC id** | **Score** |
| --- | --- | --- | --- | --- | --- | --- | --- |
|  | PAWR | Pro-Apoptotic WT1 Regulator | Protein Coding | Q96IZ0 | 48 | GC12M079574 | 45.52 |
|  | NR1I2 | Nuclear Receptor Subfamily 1 Group I Member 2 | Protein Coding | O75469 | 50 | GC03P119780 | 45.77 |
|  | MAPK8 | Mitogen-Activated Protein Kinase 8 | Protein Coding | P45983 | 56 | GC10P048306 | 48.68 |
|  | MAPK10 | Mitogen-Activated Protein Kinase 10 | Protein Coding | P53779 | 56 | GC04M085990 | 48.35 |
|  | FosB | FosB Proto-Oncogene, AP-1 Transcription Factor Subunit | Protein Coding | P53539 | 49 | GC19P045467 | 61.6 |
|  | DAPK1 | Death Associated Protein Kinase 1 | Protein Coding | P53355 | 56 | GC09P087497 | 62.09 |
|  | ITM2B | Integral Membrane Protein 2B | Protein Coding | Q9Y287 | 51 | GC13P048233 | 72.9 |
|  | EIF2A | Eukaryotic Translation Initiation Factor 2A | Protein Coding | Q9BY44 | 48 | GC03P150546 | 42.12 |
|  | CDK6 | Cyclin Dependent Kinase 6 | Protein Coding | Q00534 | 58 | GC07M092604 | 70.58 |
|  | XBP1 | X-Box Binding Protein 1 | Protein Coding | P17861 | 52 | GC22M028794 | 64.22 |
|  | PTGS2 | Prostaglandin-Endoperoxide Synthase 2 | Protein Coding | P35354 | 56 | GC01M186671 | 61.04 |
|  | VEGFA | Vascular Endothelial Growth Factor A | Protein Coding | P15692 | 56 | GC06P043770 | 63.31 |
|  | VEGFB | Vascular Endothelial Growth Factor B | Protein Coding | P49765 | 50 | GC11P064234 | 30.7 |
|  | UPP2 | Uridine Phosphorylase 2 | Protein Coding | O95045 | 42 | GC02P157876 | 30.64 |
|  | BCL2 | BCL2 Apoptosis Regulator | Protein Coding | P10415 | 58 | GC18M063123 | 96.39 |
|  | MMP1 | Matrix Metallopeptidase 1 | Protein Coding | P03956 | 57 | GC11M109978 | 43.49 |
|  | MMP2 | Matrix Metallopeptidase 2 | Protein Coding | P08253 | 60 | GC16P055390 | 60.13 |
|  | MMP9 | Matrix Metallopeptidase 9 | Protein Coding | P14780 | 60 | GC20P046008 | 55.32 |
|  | MMP11 | Matrix Metallopeptidase 11 | Protein Coding | P24347 | 50 | GC22P023768 | 41.65 |
|  | IHH | Indian Hedgehog Signaling Molecule | Protein Coding | Q14623 | 53 | GC02M219054 | 44.33 |
|  | HHAT | Hedgehog Acyltransferase | Protein Coding | Q5VTY9 | 48 | GC01P210328 | 54.64 |
|  | DHH | Desert Hedgehog Signaling Molecule | Protein Coding | O43323 | 49 | GC12M050433 | 45.43 |
|  | SHH | Sonic Hedgehog Signaling Molecule | Protein Coding | Q15465 | 57 | GC07M155799 | 92.82 |
|  | CDK4 | Cyclin Dependent Kinase 4 | Protein Coding | P11802 | 61 | GC12M058711 | 83.95 |
|  | SUFU | SUFU Negative Regulator of Hedgehog Signaling | Protein Coding | Q9UMX1 | 50 | GC10P102503 | 79.82 |
|  | PTCH1 | Patched 1 | Protein Coding | Q13635 | 58 | GC09M095442 | 74.35 |
|  | PTCH2 | Patched 2 | Protein Coding | Q9Y6C5 | 52 | GC01M044819 | 44.55 |
|  | GLI1 | GLI Family Zinc Finger 1 | Protein Coding | P08151 | 56 | GC12P058191 | 73.57 |
|  | GLI2 | GLI Family Zinc Finger 2 | Protein Coding | P10070 | 56 | GC02P120735 | 18.79 |
|  | GLI3 | GLI Family Zinc Finger 3 | Protein Coding | P10071 | 56 | GC07M041960 | 9.98 |

**Supplementary table S4.** Grid and Binding site amino acid Details

| **PDB id of proteins** | **Grid details - inner box (X-Y-Z)** | **Grid details - outer box (X-Y-Z)** | **Central Coordinates (X-Y-Z)** | **Binding site amino acid details obtained from PDBsum webtool and Sitemap (Schrodinger suite 2023-2)** |
| --- | --- | --- | --- | --- |
| 7QIU | 16  10  10 | 32.714  26.714  26.714 | 14.489  23.986  107.4761 | LEU18(A), HIS19(A), LYS21(A), ARG24(A), PHE30(A), LEU31(A), GLU33(A), THR81(A), GLU82(A), THR83(A), GLU84(A), THR85(A), ARG24(A), ASP141(A), PHE143(A), ASN144(A), GLY145(A), LYS146(A), LEU148(A), ARG149(A) |
| 4KMH | 17  9  8 | 33.9036  25.9036  24.9036 | 11.705  4.769  44.516 | PHE149(A), ASN102(A), ARG103(A), VAL104(A), HIS105(A), TYR60(A), TRP61(A), LEU62(A), GLY63(A), GLU448(A) HIE394(B), THR396(B), LYS398(B), VAL269(B), GLY268(B), SER267(B) |
| 5OMO | 26  10  12 | 51.9533  35.9533  37.9533 | -45.904  10.073  -86.819 | **RL:SO_4 ¯_**  ARG 196 (A), ILE 192 (A), GLU 193 (A), PRO 191(A), LEU526(A), VAL528(A), |
| 4BLD | 17  10  11 | 33.786  26.786  27.786 | 5.878  -16.246  -83.804 | **RL: SER-TYR-GLY-HIS-LEU-SER-ALA-SER**  LEU480(A), HIS 500 (A), SER 607 (A), GLY 608 (A), SER 610 (A), SER 502 (A), GLU 641 (A), TYR 487 (A), ASP 499 (A), VAL 501 (A), LYS 663 (A), PHE 495 (A) |
| 3N1O | 13  12  10 | 28.1374  27.1374  25.1374 | 8.201  -33.275  42.545 | MET734 (A), TYR735 (A), ILE736 (A), ALA714 (A), GLU68 (A), ILE71 (A), ARG77 (A), PHE78 (A), LYS79 (A), GLU80 (A), LEU81 (A), THR82 (A), PRO83 (A), TYR85 (A), ASN86 (A), PRO87 (A), ILE89 (A), ILE90 (A), PHE91 (A), ARG101 (A), LEU102 (A), THR104 (A), GLN105 (A), ARG106 (A), LYS108 (A), ASP109 (A), ARG110 (A), ASN112 (A), THR130 (A), GLU131 (A), HIS139 (A), SER140 (A), HIS149 (A), GLU147 (A), ARG149 (A), ASP152 (A), TRP177 (A), TYR179 (A), GLU181 (A), HIS185 (A), LYS191 (A), SER192 (A), GLU193 (A), TYR85 (B), PRO87 (B), ASP88 (B), ILE89 (B), ILE90 (B), LYS43 (C), LEU44 (C), VAL45 (C), SER53 (C), PRO54 (C), VAL56 (C), LEU61 (C), GLY62 (C), GLN105 (C), ARG106 (C), ASP109 (C), ARG110 (C), SER113 (C), SER117 (C), ASN120 (C), GLN121 (C), ARG168 (C), LEU169 (C), VAL171 (C), GLU172 (C), ALA173 (C), GLY174 (C), PHE175 (C), ASP176 (C), VAL178 (C), LYS191 (C), SER192 (C), GLU193 (C) |
| 3N1G | 16  10  11 | 32.6709  26.6709  27.6709 | 10.884  -11.062  40.092 | PRO716(A), TYR717(A), MET734(A), TYR735(A), ILE736(A), ALA714(A), GLY715(A), PRO716(A), TYR717(A), ILE718(A), GLU800(A), PHE801(A), ASN803(A), SER802(A) |
| 3N1M | 16  11  6 | 32.3019  27.3019  22.3019 | 20.590  -23.262  6.332 | HIS138(B), HIE139(B), ZN194(B), SER140(B), SER143(B), HIS145(B), GLU181(B), HIS187(B), HIS185(B), ASP152(B), LYS183(B), TYR179(B), GLU783(C), GLU808(C), THR809(C), LYS810(C), LYS810(C), ALA811(C) |
| 4WPB | 9  6  10 | 20.6003  17.6003  21.6003 | 25.993  -21.970  34.273 | THR31(A), ILE29V, ARG56(A), CYS57(A), GLY58(A), GLY59(A), CYS60(A), CYS61(A), ASN62(A), ASP63(A), LEU66(A), GLU67(A), CYS68(A), CYS57(B), GLY58(B), GLY59(B)  MET94(B), SER95(B),  PHE96(B), LEU97(B),  GLN98(B), HIS99(B),  ASN100(B), LYS101(B),  CYS102(B), GLU103(B),  CYS104(B), ARG105(B),  PRO106(B), GLU8(C),  CYS10(C), ASN11(C),  ARG13(C), ALA14(C),  ILE15(C), GLU16(C),  AIB17(C), ALA18(C),  LEU19(C), ASP20(C),  PRO21(C), ASN22(C) |
| 2VWE | 7  11  15 | 23.0754  27.0754  31.0754 | 23.326  -7.229  30.407 | VAL31(A), HIS98(A), ARG56(A), CYS57(A), GLY58(A), GLY59(A), CYS60(A), CYS61(A), PRO62(A), ASP63(A), ASP64(A), LEU66(A), LYS106(A), GLU67(A), CYS68(A), VAL69(A), |
| 4TTH | 13  15  10 | 30.0749  32.0749  27.0749 | -32.843  24.966  -14.419 | **RL:24V**  HID100(A), ASP102(A), ASP104(A), THR106(A), PHE 98 (B), ALA41(B), VAL27(B), VAL77(B), GLU99(B), HIS100 (B), VAL101(B), LEU152(B), ALA162(B), ASP102(B), GLA103(B), GLN149(B), ASP104(B), THR107(B) |
| 3SHI | 16  10  12 | 31.8322  25.8322  27.8322 | 2.586  -29.362  2.634 | **RL:CA305(A)**  ASP194 (A), ASP 158(A), GLY 190(A), GLY192(A), GLU209(A), GLU219(A), TYR237(A), TYR240(A) |
| 1RTG | 10  13  10 | 25.1309  28.1309  25.1309 | 34.773  5.312  12.359 | **RL:CA5(A)**  ASP476(A), GLU484(A), ASP521(A), ASP569(A), LEU617(A), ASP618(A) |
| 5CUH | 10  10  10 | 25.6626  25.6626  25.6626 | 13.520  20.828  -3.038 | **RL: LTQ**  GLU 227(A), HIS230(A), ALA189 (A), LEU187(A), LEU188(A), TYR 248(A), HIS226(A), GLU227(A), VAL223(A), TYR245(A), MET247(A), LEU222(A), LEU 243(A), HIS236(A), GLU227(A), ZN301(A) |
| 1CVU | 13  10  11 | 27.3401  24.3401  25.3401 | 27.963  24.327  47.359 | **RL: ARACHIDONIC ACID**  GLY45(A), TYR385(A), MET522 (A), GLY 526 (A), TRP 387 (A), PHE 518 (A), ALA 527 (A), VAL 349 (A), ILE 345 (A), LEU 534 (A), TYR 348 (A), TYR 385 (A), PHE 205 (A), PHE 381 (A), GLN2327(B) |
| 2G01 | 10  10  11 | 22.0248  22.0248  23.0248 | 24.884  93.791  28.396 | **RL: 73Q**  ALA 113 (A), ASN 114 (A), VAL 158 (A), LEU 110 (A), LEU 168 (A), ALA 53 (A), MET 108 (A), MET 111 (A), ILE 32 (A) |
| 2JK9 | 19  11  17 | 37.9258  29.9258  35.9258 | 2.945  41.176  39.922 | RL- MG1144 (A)  ASP13(A), THR35(A), ARG36(A), LEU37(A) ASP38(A), HID54(A), ASP55(A), ARG57(A), LYS90(A), VAL91(A), GLY92(A), TYR93(A), THR94(A), ARG95(A), GLY96(A), LEU97(A), ALA180(A), ASP182(A), ASP185(A), TYR196(A), |
| 1G5M | 11  6  8 | 25.5743  20.5743  22.5743 | -2.133  7.780  11.327 | **VARIANT- GLY203SER**  TRY9(A), ARG12(A), PRO204(A), GLY203(A), ASN182(A), ASP196(A), VAL199(A), TYR202(A), ALA96(A), LEU 201(A) |
| 5VPB | 12  10  10 | 25.9684  23.9684  23.9684 | 275.604  -17.159  60.436 | GLU191(A), LEU296(A), ASP188(A), THR187(A), GLN184(A), ALA169(C), CYS172(C), ARG173(C), ARG175(C), CYS285(D), ARG288(D), LYS289(D), THR180(C), ARG292(D), ARG295(D) |
| 4S0S | 10  14  10 | 24.2596  28.2596  24.2596 | -39.967  40.647  -26.544 | LEU209(A), LEU411(A), ILE414(A), LEU240(A), PRO241(A), PHE420(A), MET243(A), ALA244(A), MET425(A), SER247(A), PHE429(A), PHE281(A), PHE251(A), HIS407(A), MET323(A), HID327(A), CYS284(A), GLN285(A), PHE288(A), TRP299(A), TYR306(A) |
| 4Y5H | 13  13  10 | 30.5781  30.5781  27.5781 | 1.157  -29.631  -30.541 | **RL- 519**  MET 149 (A), GLN 155 (A), ILE 70 (A), ASP 150 (A), LEU 206 (A), ALA 91 (A), ALA 151 (A), ASN 152 (A), GLU 147 (A), ILE 124 (A), VAL 78 (A), LEU 148 (A), MET 146 (A) |
| 5AUT | 10  11  10 | 22.8177  23.8177  22.8177 | -22.661  1.816  -10.741 | **RL-2AN**  GLU100(A), ASP 161 (A), LYS 42 (A), LEU 93 (A), VAL96 (A), GLU143(A), ILE 160 (A), GLU 94 (A), VAL 27 (A), ALA 25 (A), ALA 40 (A), MET 146 (A), LEU 19 (A), GLY 20 (A), GLY 22 (A), SER 21 (A) |

**Supplementary table S5.** 2D and 3D Docking images of Hedgehog proteins and WA network proteins

| **Hedgehog pathway related genes and proteins - 2D and 3D pictures** | | |
| --- | --- | --- |
| **LIGAND** | 3D Picture | 2D Picture |
| **1. 7QIU –Hedgehog acyltransferase (HHAT) (Chain A)** | | |
| TAM | 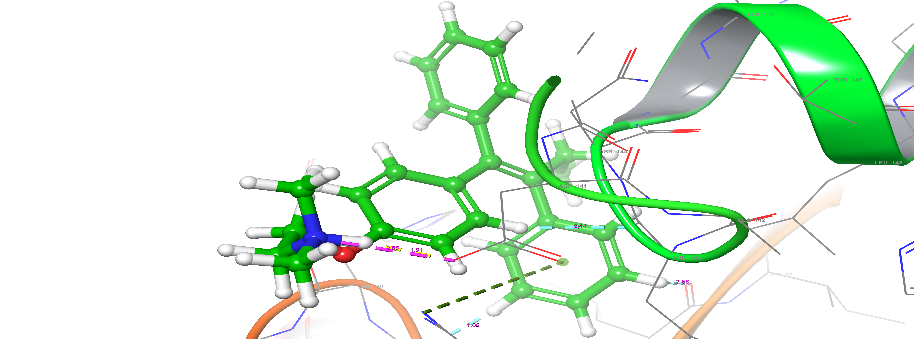 | 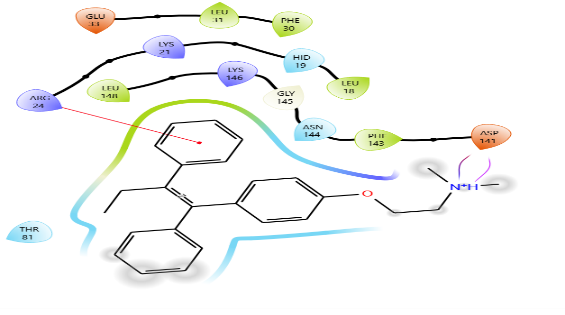 |
| WA | 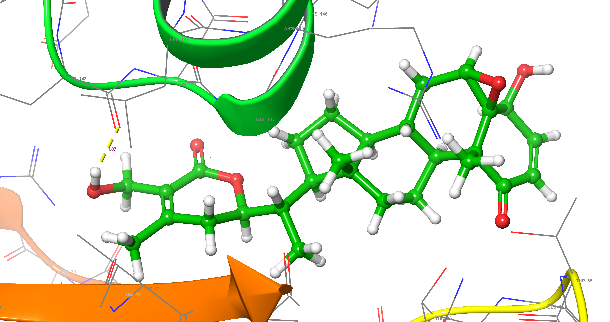 | 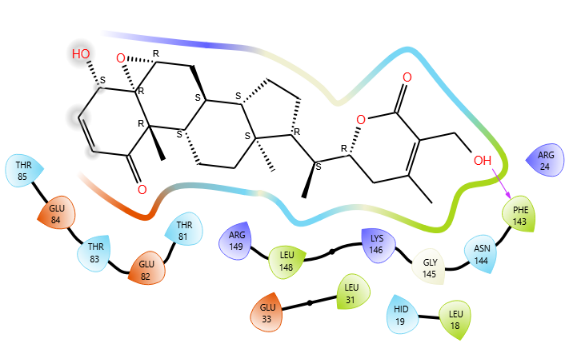 |
| **2. 4KMH - Suppressor of fused (SUFU) (Chain A and B)** | | |
| TAM | 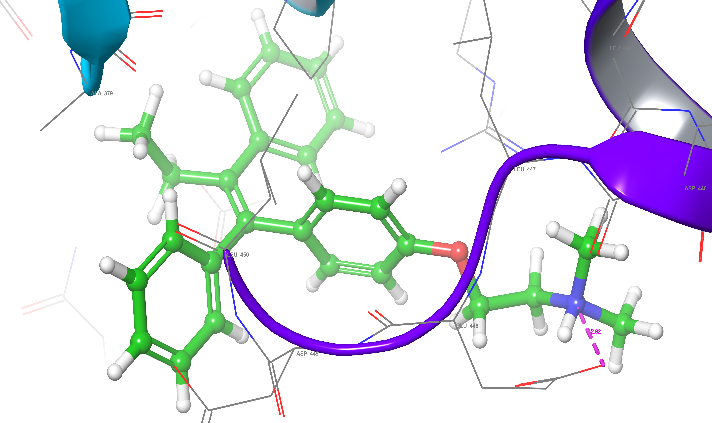 | 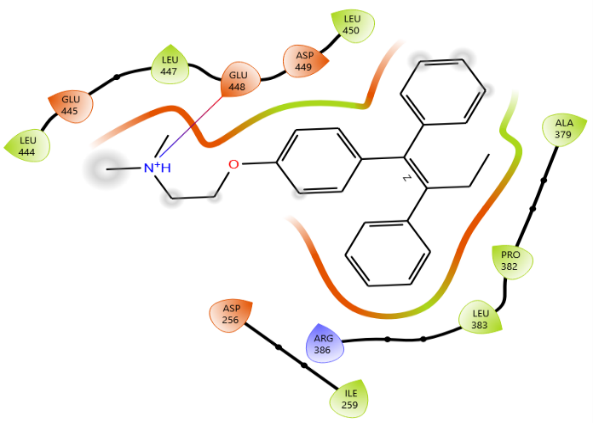 |
| WA | 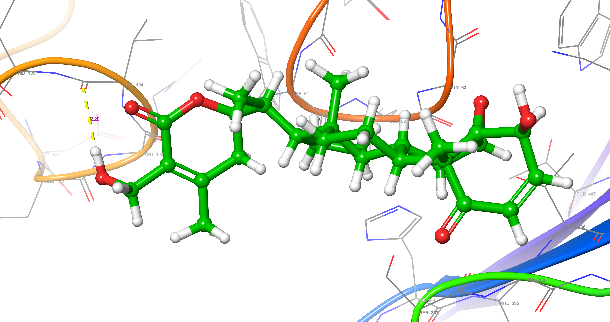 | 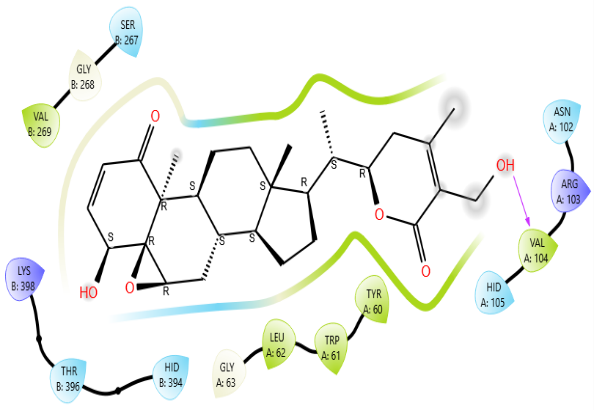 |
| **3. 5OMO – Glioma associated protein1 (GLI1) (Chain A and B)** | | |
| TAM | 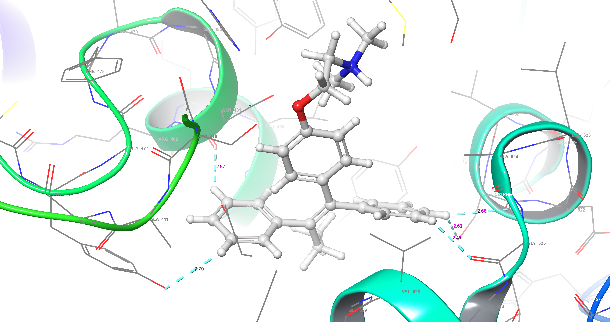 | 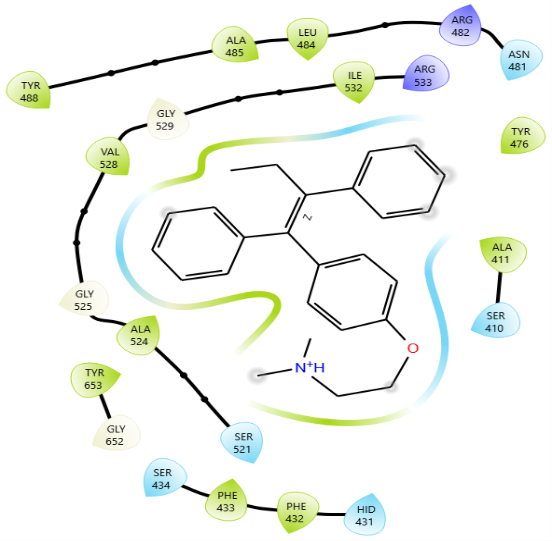 |
| WA | 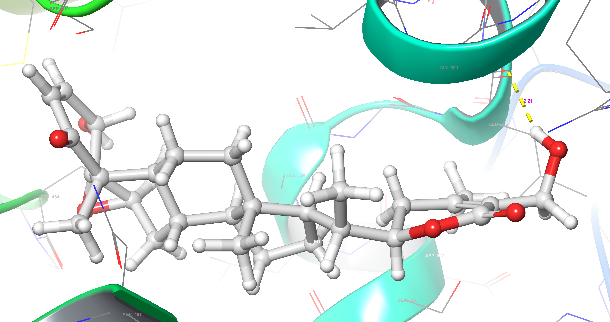 | 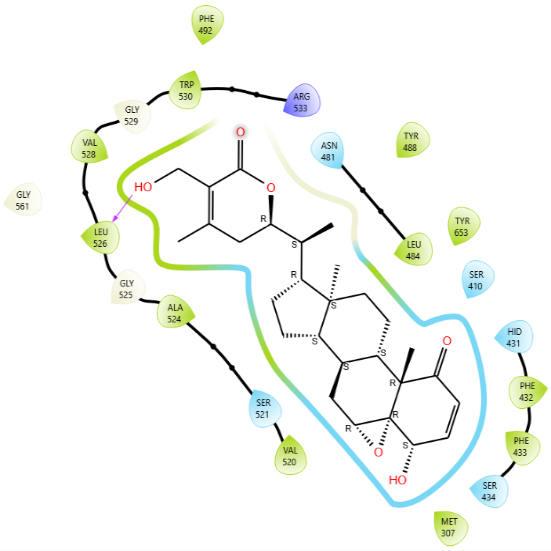 |
| **4. 4BLD – Glioma associated protein3 (GLI3) (Chain E, F, G, H)** | | |
| TAM | 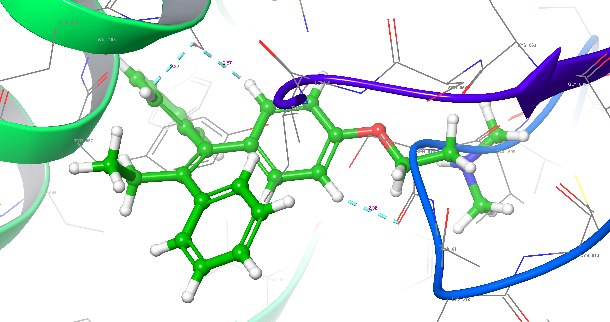 | 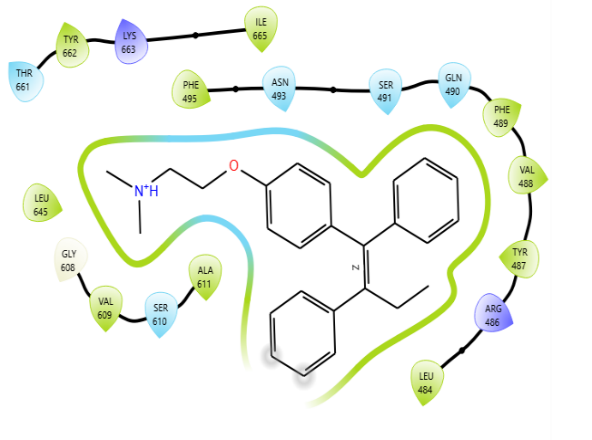 |
| WA | 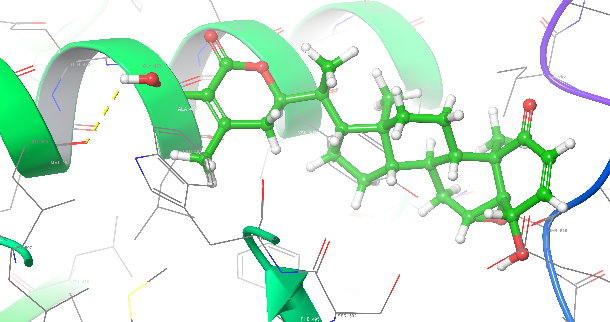 | 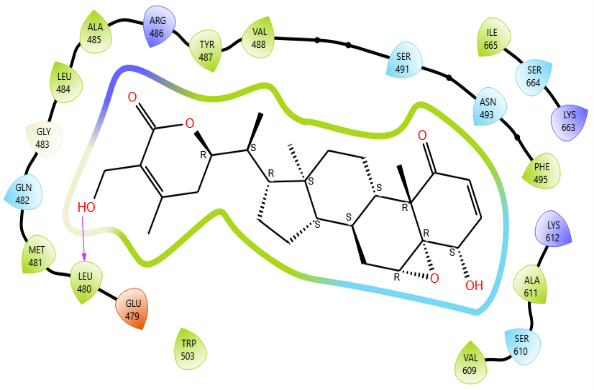 |
| **5. 3N1O – Indian Hedgehog (IHH) (Chain A, B, C)** | | |
| TAM | 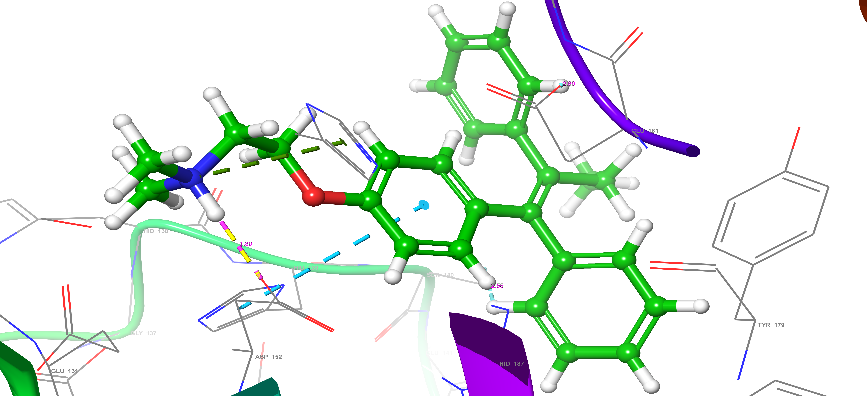 | 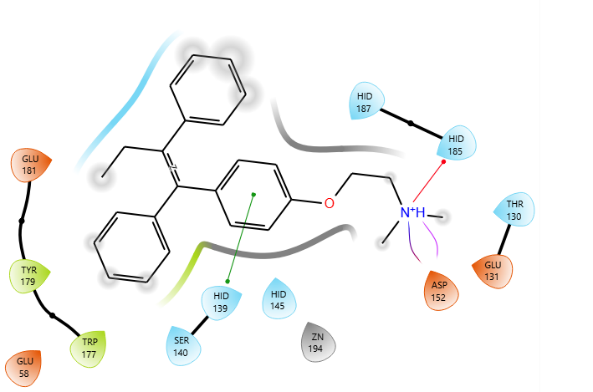 |
| WA | 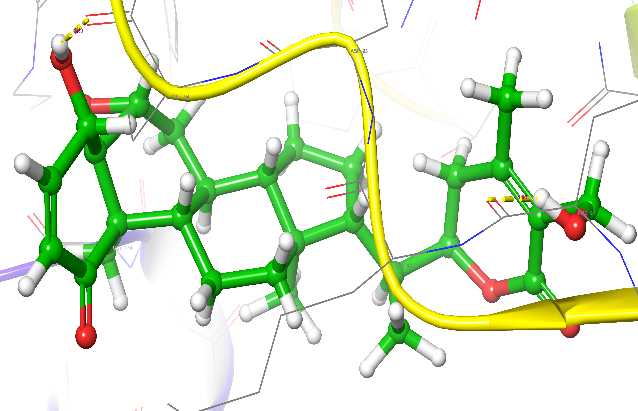 | 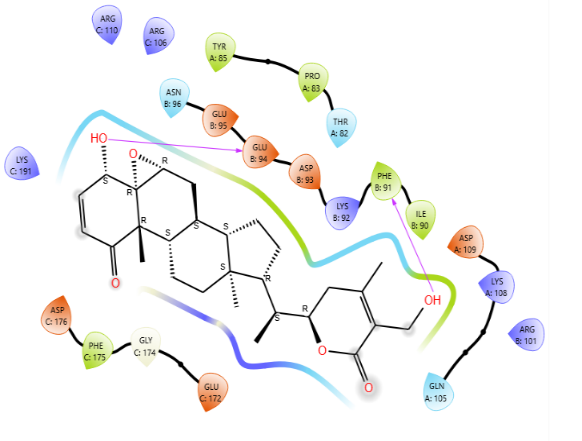 |
| **6. 3N1G – Desert Hedgehog/ Mammalian hedgehog (DHH) (Chain C and A)** | | |
| TAM | 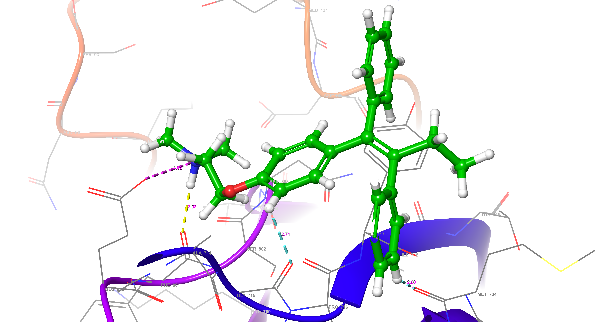 | 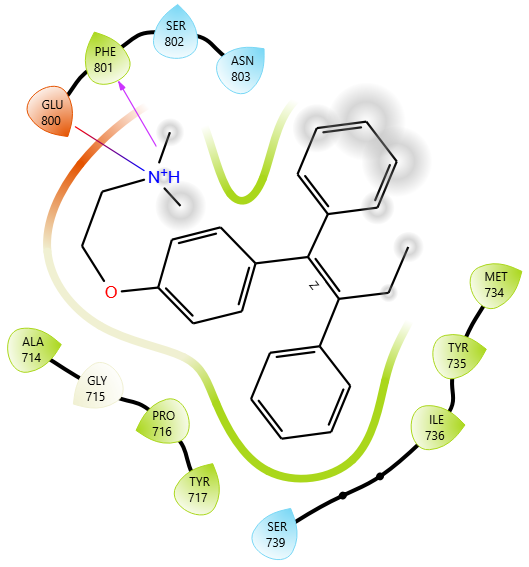 |
| WA | 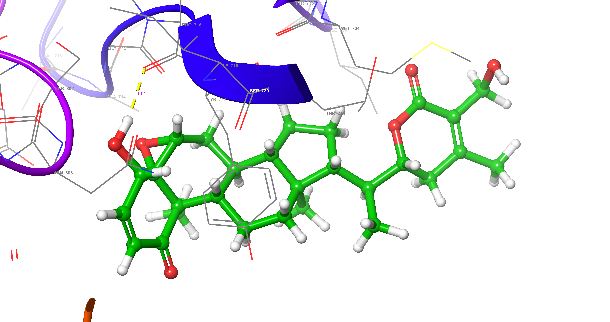 | 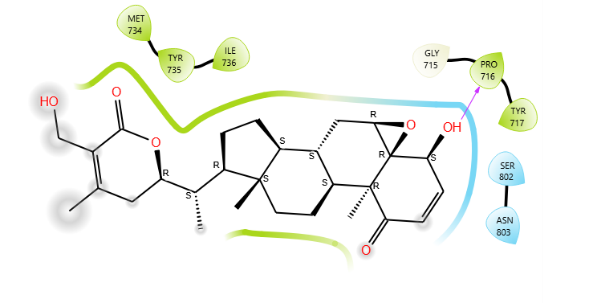 |
| **7. 3N1M – Indian Hedgehog (Chain A and B)** | | |
| TAM | 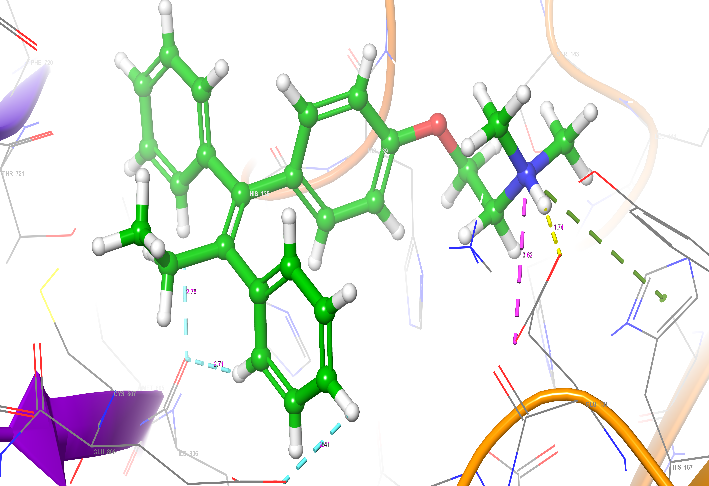 | 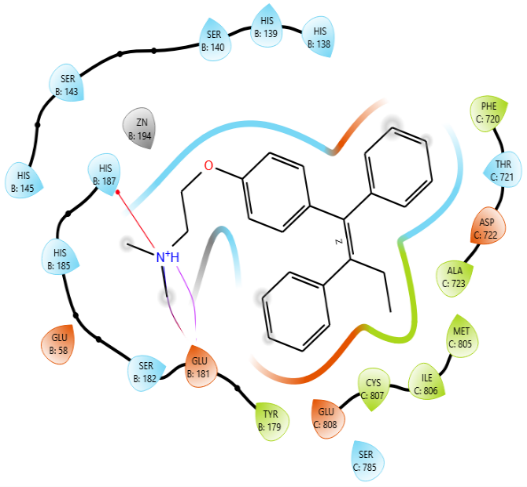 |
| WA | 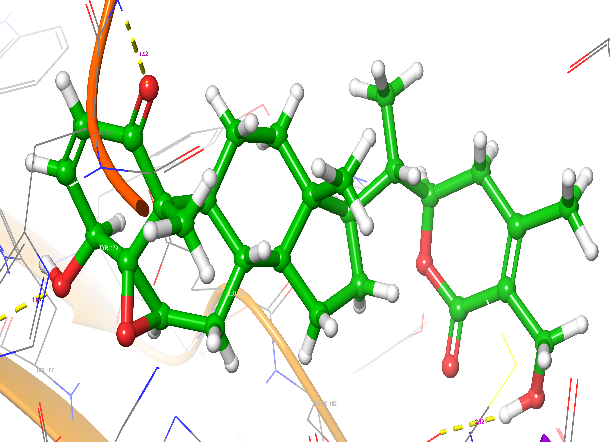 | 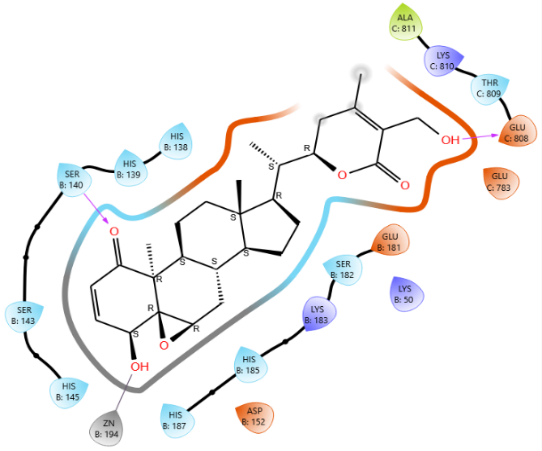 |
| **8. 4WPB - VEGF and VEGF-A (Chain A and B)** | | |
| TAM | 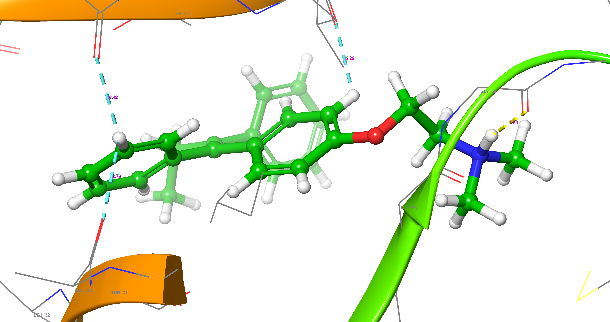 | 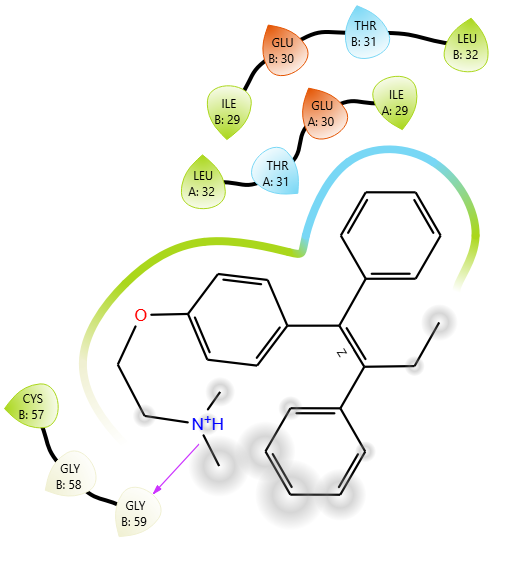 |
| WA | 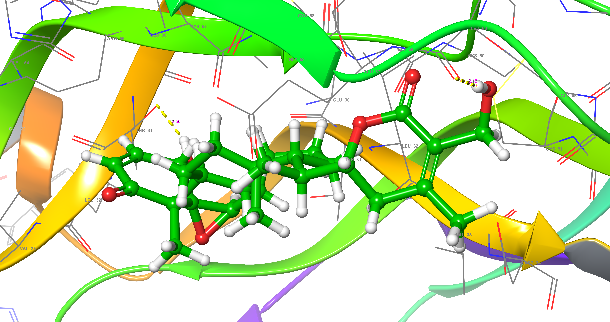 | 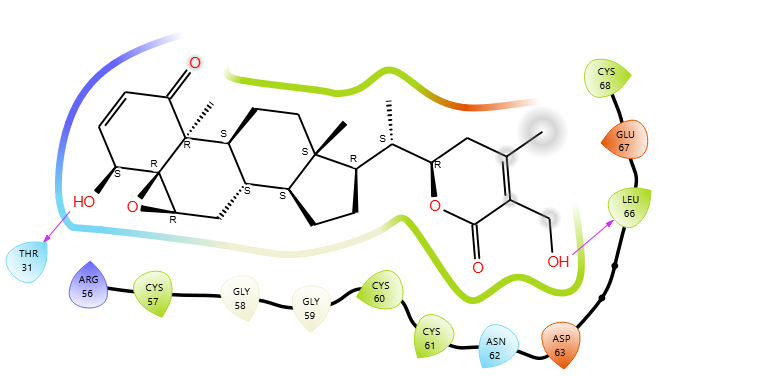 |
| **9. 2VWE - Vascular Endothelial Growth Factor-B (Chain A and B)** | | |
| TAM | 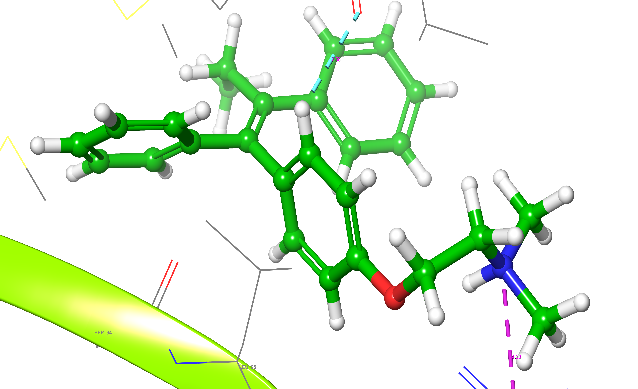 | 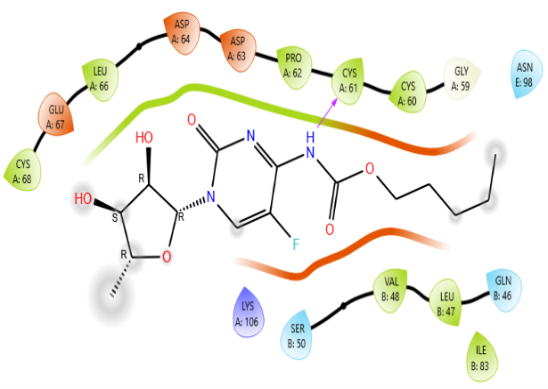 |
| WA | 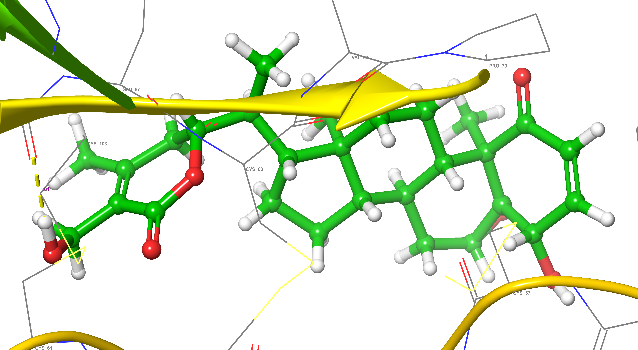 | 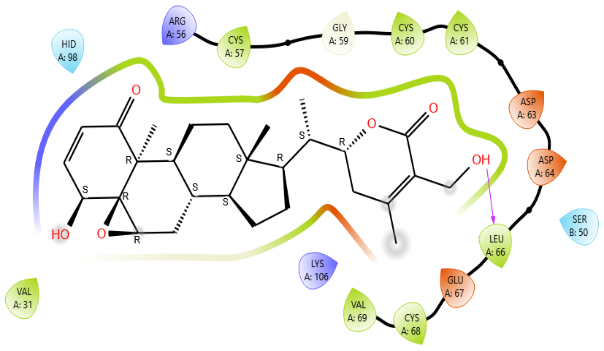 |
| **10. 4TTH – Crystal structure of a Cyclin dependant kinase6 (CDK6) (Chain B)** | | |
| TAM | 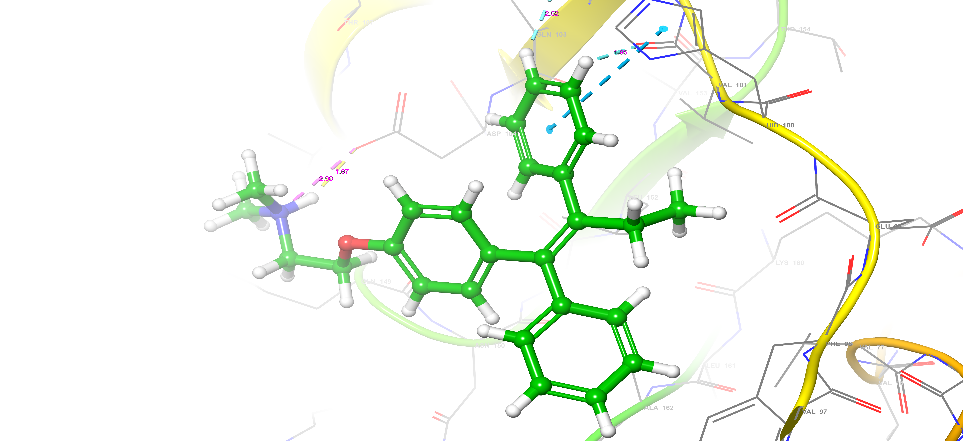 | 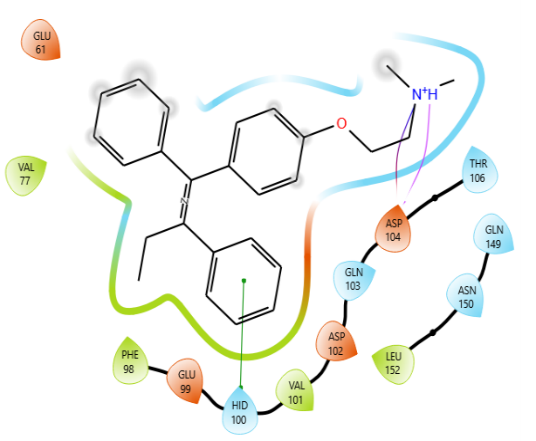 |
| WA | 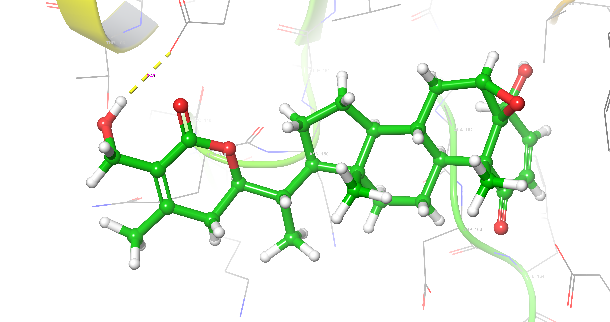 | 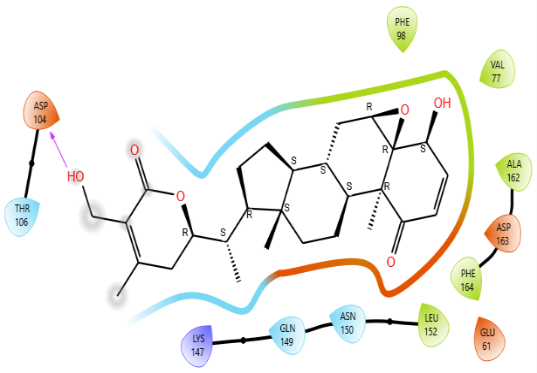 |
| **11. 3SHI – Human Matrix Metalloproteinase-1/MMP-1 (Chain A, B, C, D)** | | |
| TAM | 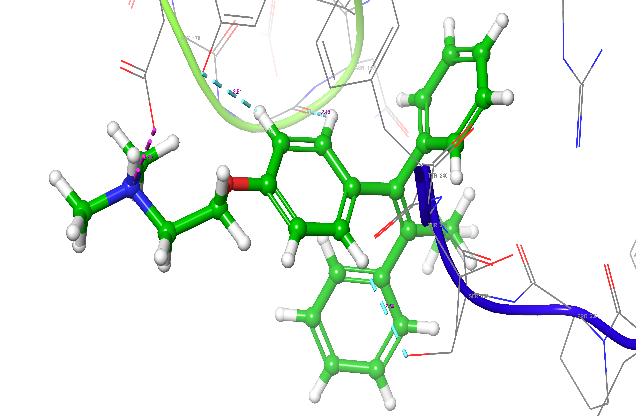 | 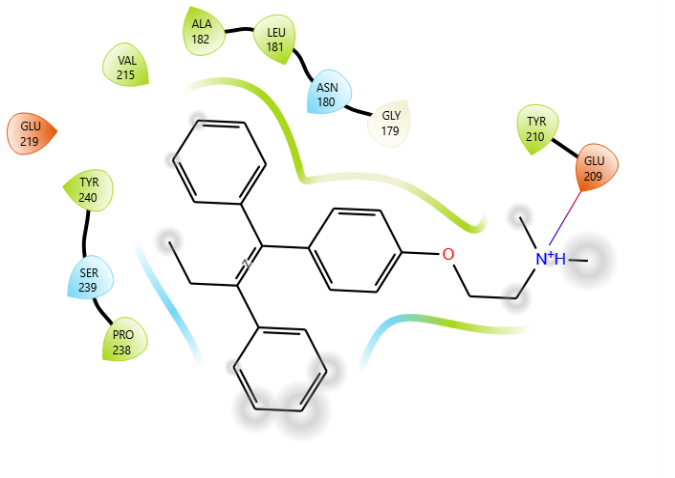 |
| WA | 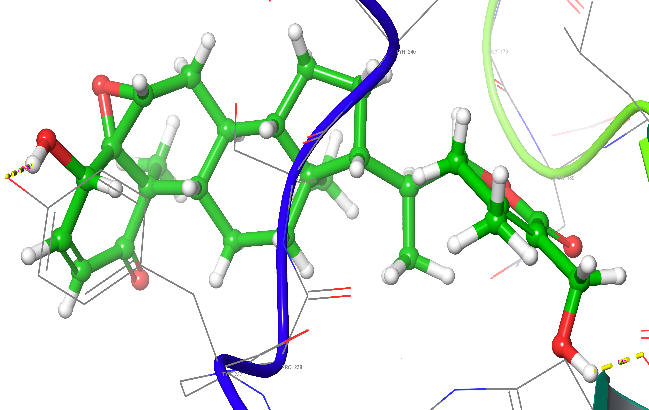 | 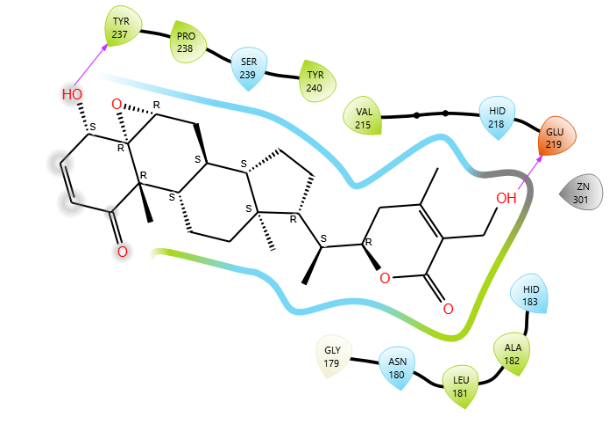 |
| **12. 1RTG – Human Matrix Metalloproteinase-2/MMP-2 (Chain A)** | | |
| TAM | 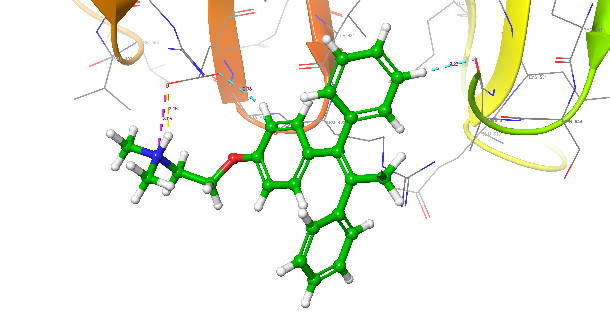 | 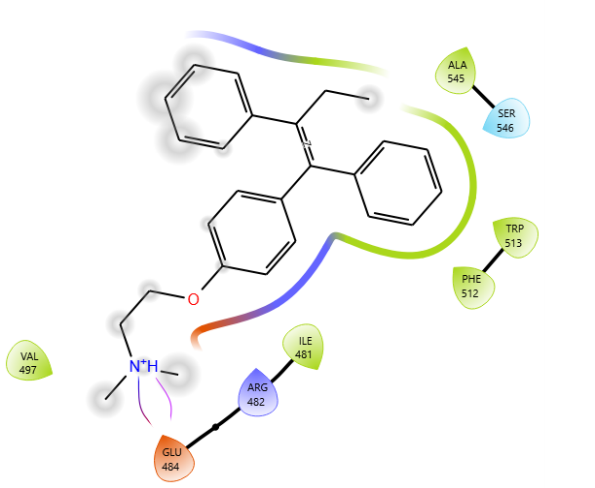 |
| WA | 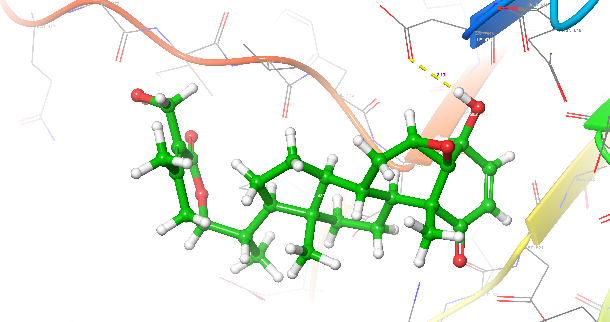 | 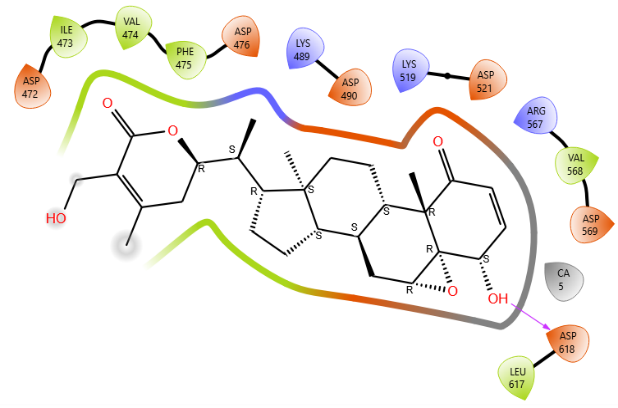 |
| **13. 5CUH – Human Matrix Metalloproteinase-9/MMP-9 (Chain A, B)** | | |
| TAM | 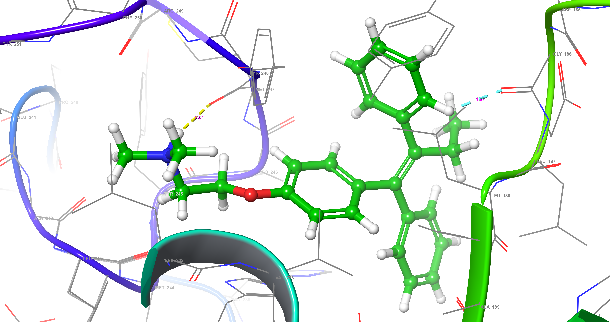 | 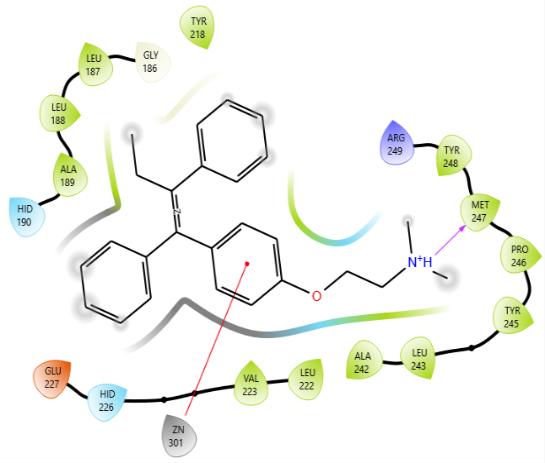 |
| WA | 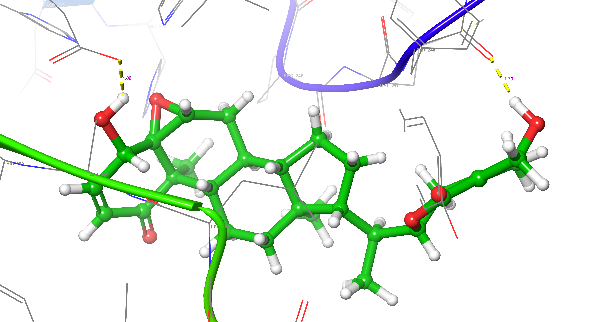 | 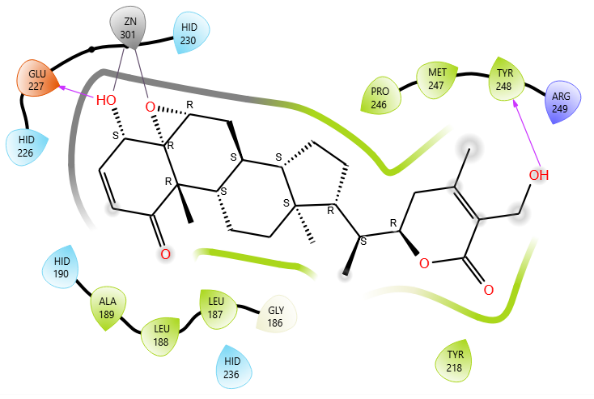 |
| **Genes from WA network – 2D and 3D pictures** | | |
| **1. 1CVU - Prostaglandin synthase 2 (PTGS2 or COX2) (Chain A and B)** | | |
| CAP | 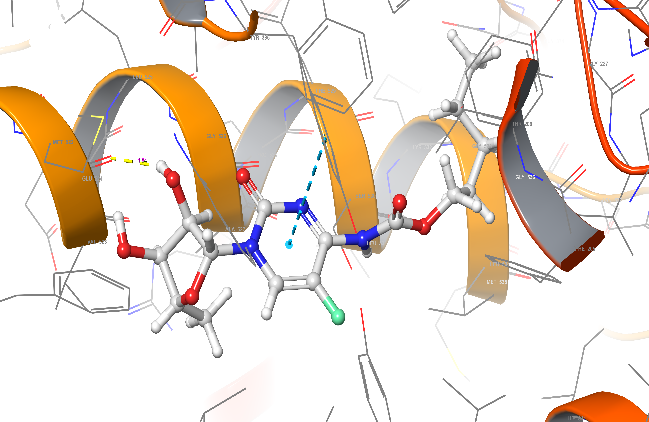 | 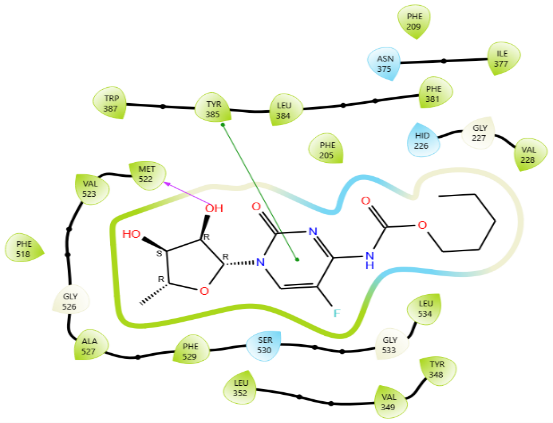 |
| WA | 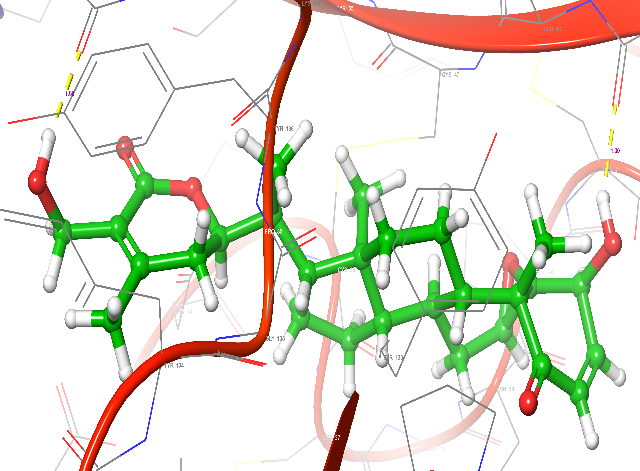 | 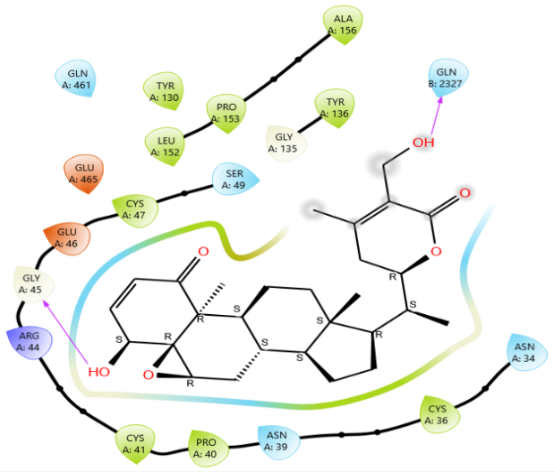 |
| **2. 2G01 – Mitogen activated kinase-8 (MAPK8 or JNK1) (Chain A, C (auth B)** | | |
| CAP | 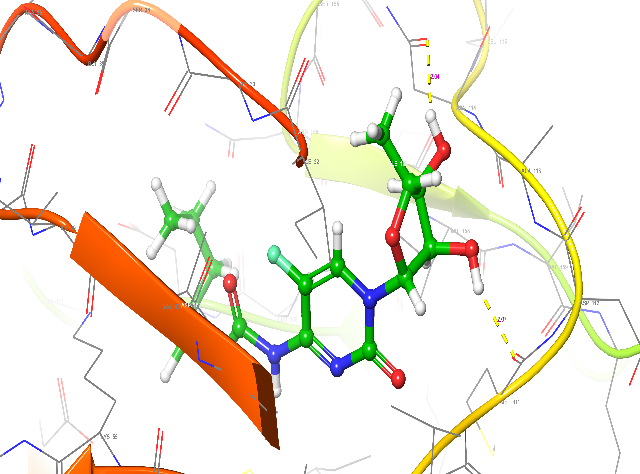 | 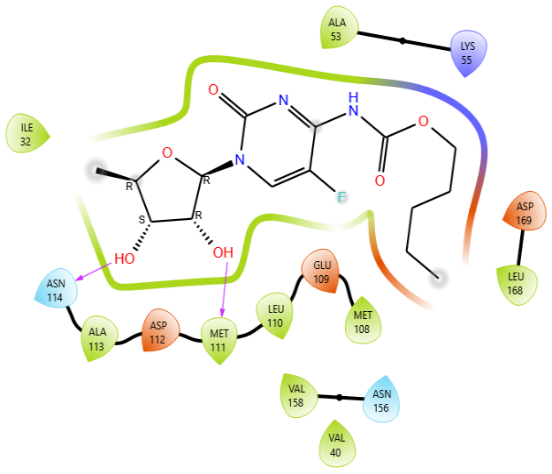 |
| WA | 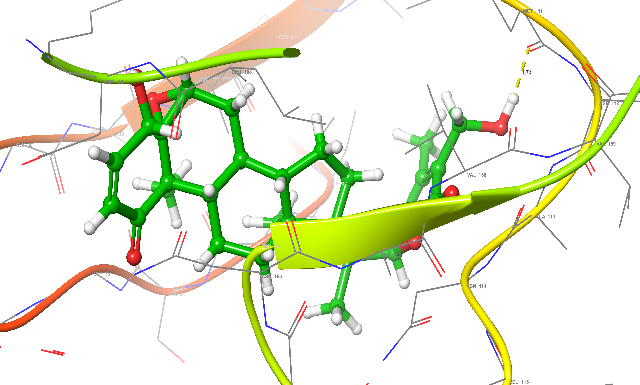 | 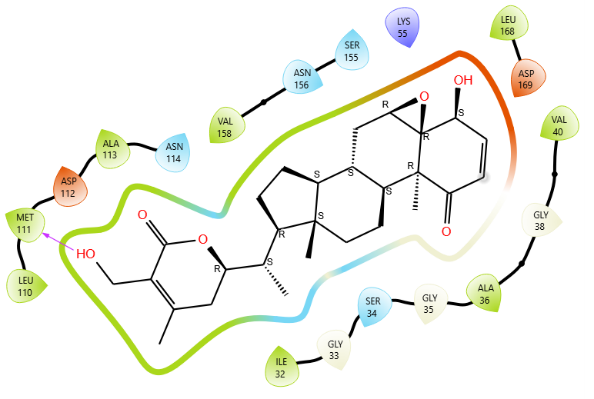 |
| **3. 2JK9 - PRKC apoptosis WT1 regulator protein/ PAWR (Chain B)** | | |
| CAP | 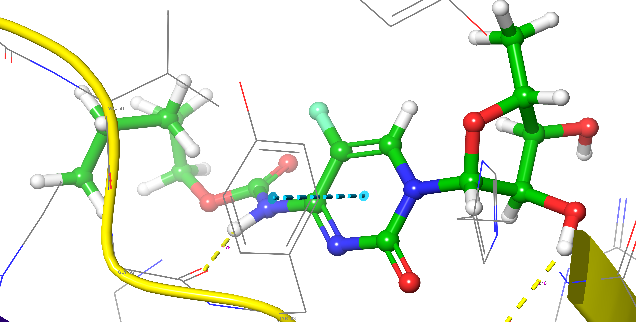 | 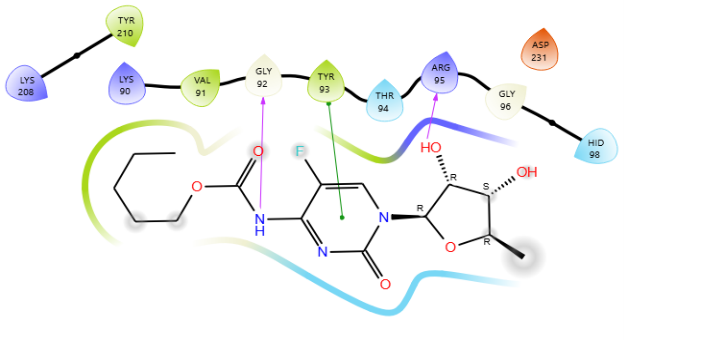 |
| WA | 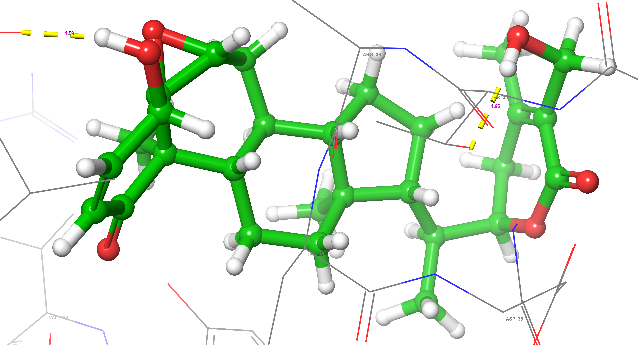 | 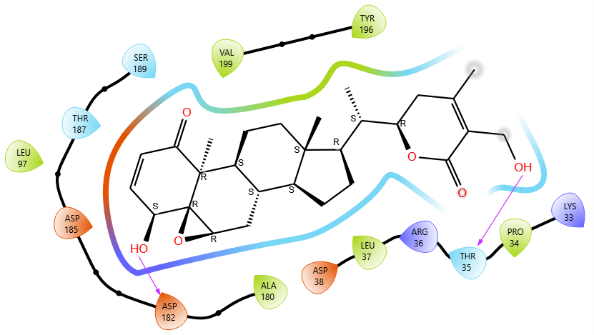 |
| **4. 1G5M – BCL2 (Chain A)** | | |
| CAP | 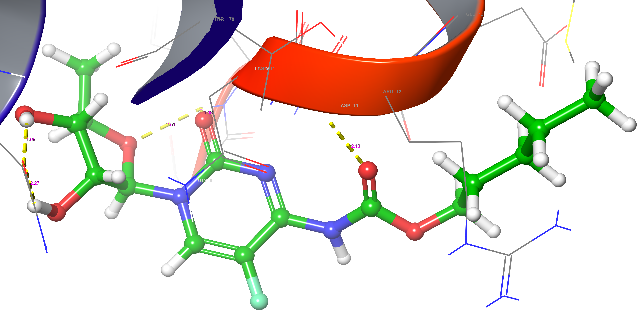 | 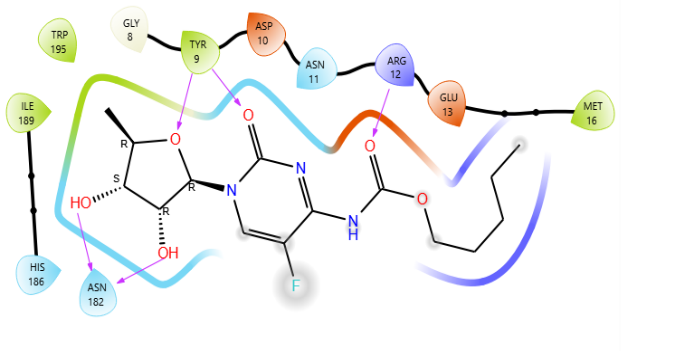 |
| WA | 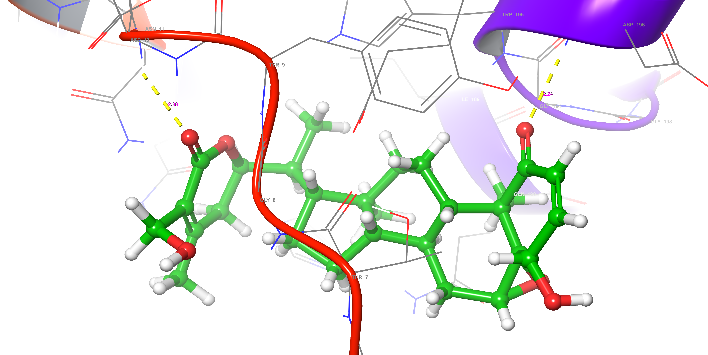 | 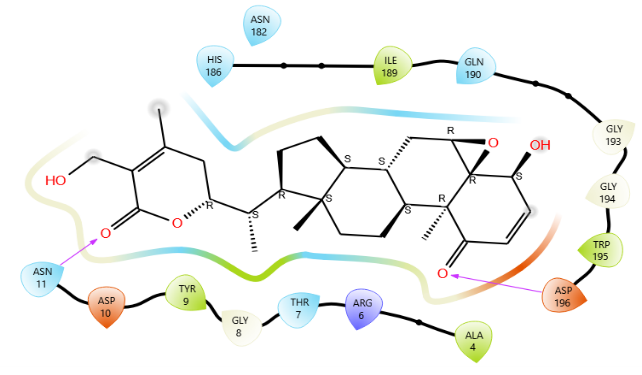 |
| **5. 5VPB – Transcription factor FosB/JunD (Chain B and D)** | | |
| CAP | 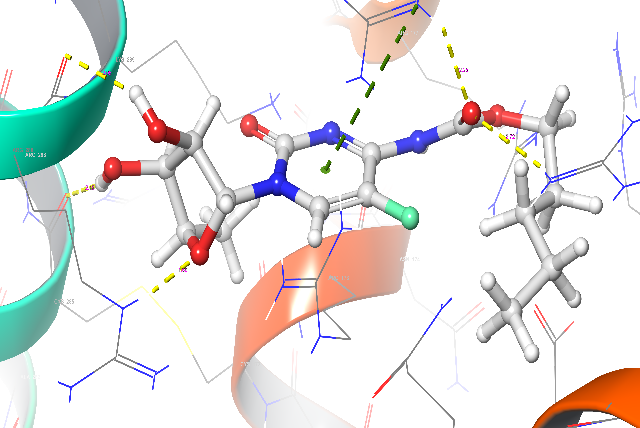 | 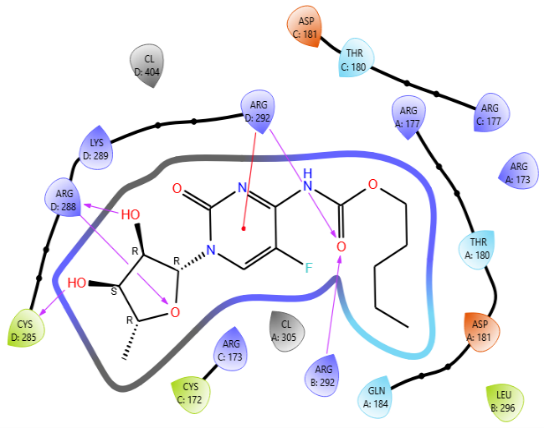 |
| WA | 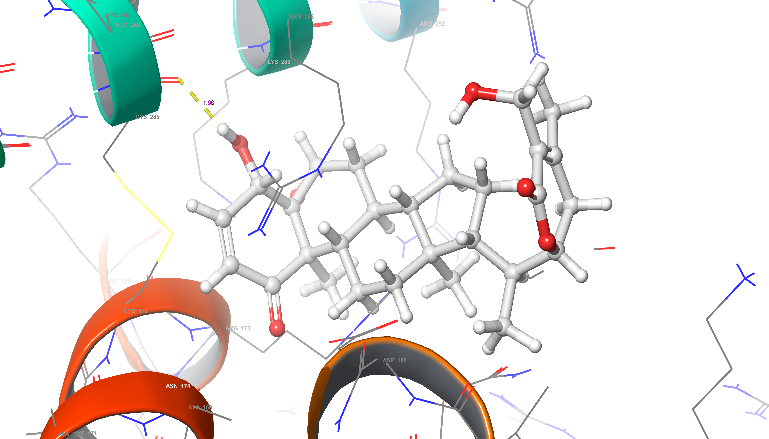 | 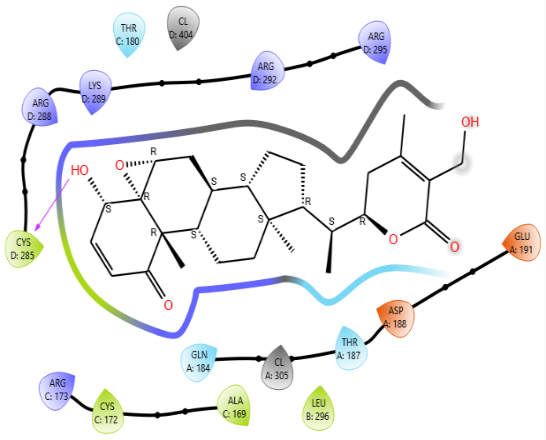 |
| **6. 4S0S – Human Pregnane X Receptor (Chain A and B)** | | |
| CAP | 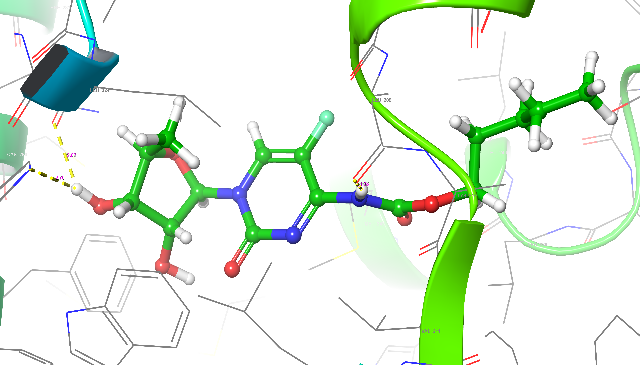 | 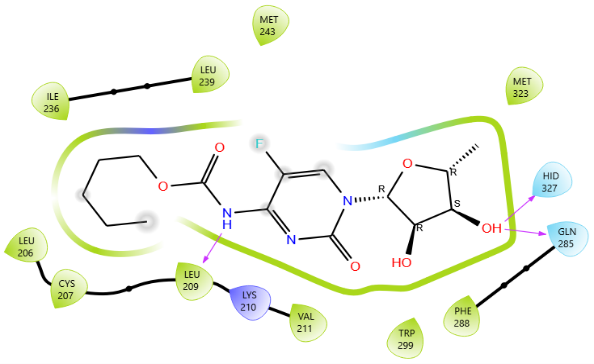 |
| WA | 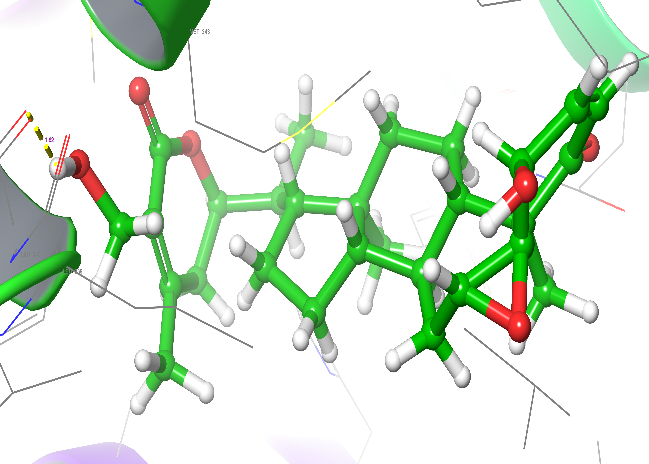 | 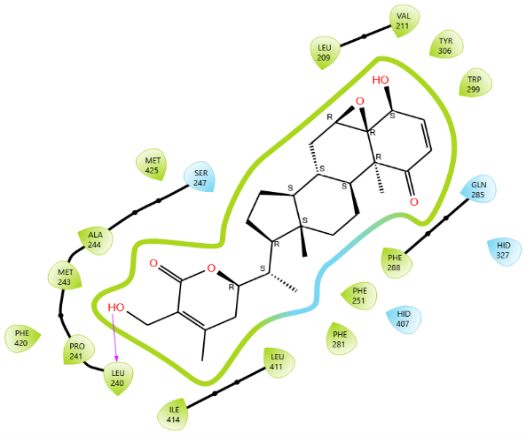 |
| **7. 4Y5H – Mitogen activated kinase 10 (MAPK10), JNK3, JNK3A (Chain A)** | | |
| CAP | 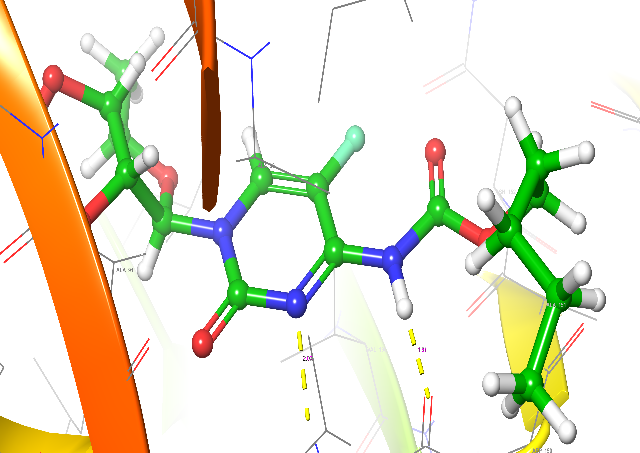 | 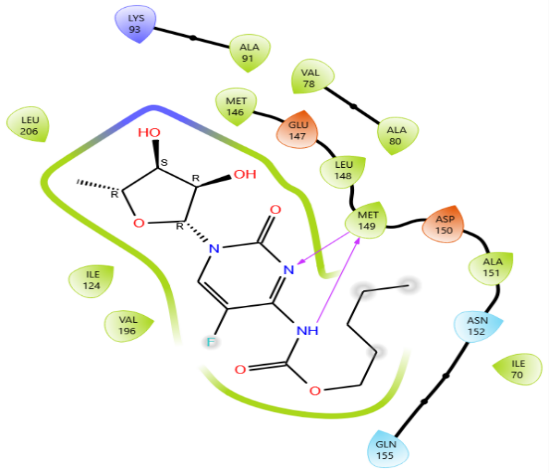 |
| WA | 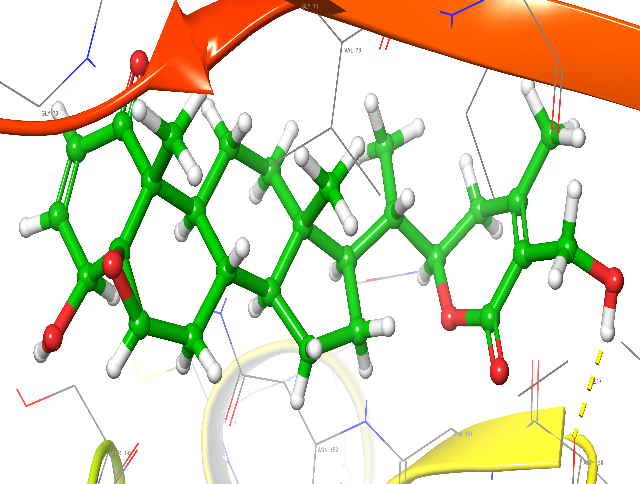 | 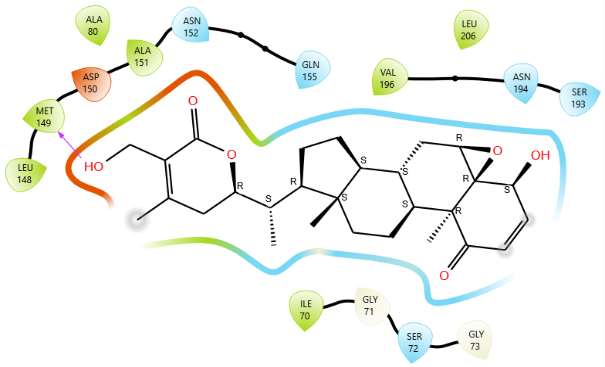 |
| **8. 5AUT – Death associated protein kinase (DAPK1, DAPK) (Chain A)** | | |
| CAP | 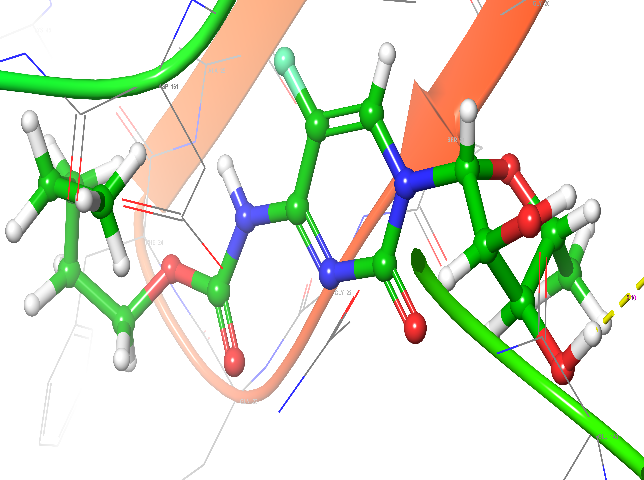 | 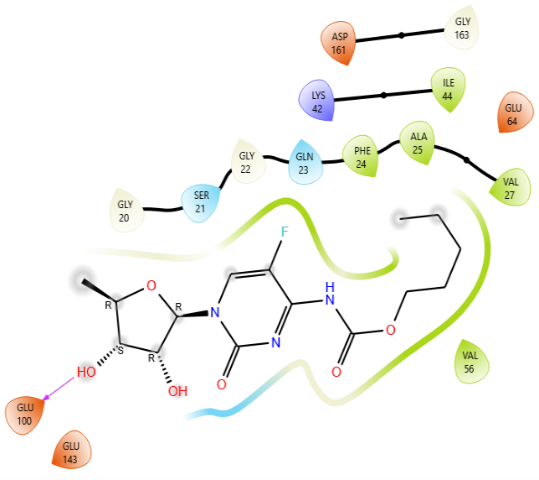 |
| WA | 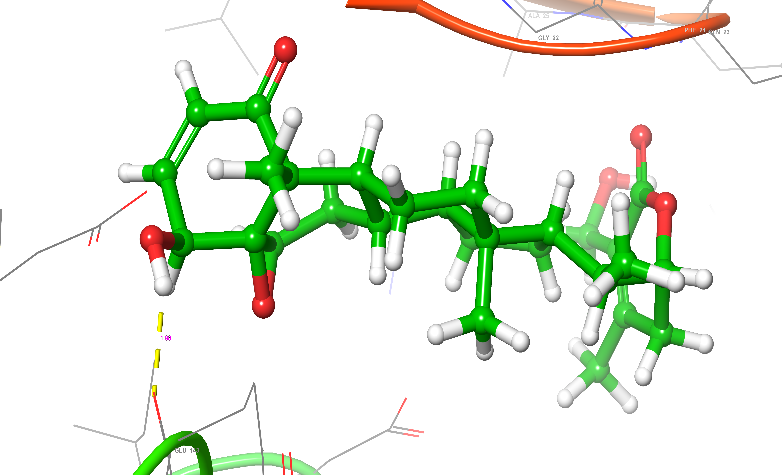 | 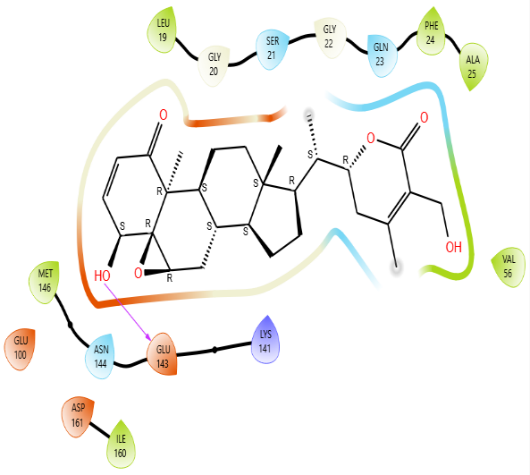 |

**Supplementary table S6.** Absorption, Distribution, Metabolism, Elimination and drug-likeness properties profile of Withaferin-A

| **Sr. No** | **Description** | **Values** |
| --- | --- | --- |
| Physicochemical properties | | |
|  | Molecular formula | C28H38O6 |
|  | Molecular weight (g/mol) | 470.270 |
|  | Hydrogen bond acceptors count | 6 |
|  | Hydrogen bond donors count | 1 |
|  | Rotatable bonds count | 3 |
|  | Topological polar surface area | 93.20 |
| Pharmacokinetic parameters | | |
| Absorption | | |
|  | GI absorption | High |
|  | Caco-2 permeability | -4.981 |
|  | MDCK permeability | 2.5e- |
|  | F30% | +++ |
| Distribution | | |
|  | PPB | 85.585% |
|  | VD | 0.741 |
|  | BBB | +++ |
|  | FU | 6.773% |
| Metabolism | | |
|  | CYP2C19 substrate | +++ |
|  | CYP2D6 substrate | + |
|  | CYP3A4 inhibitor | - |
|  | CYP3A4 substrate | ++ |
|  | P-gp inhibitor | +++ |
|  | P-gp substrate | --- |
| Excretion | | |
|  | CL | 20.058 |
|  | T½ | 0.343 |
| Bioavailability | | |
|  | Bioavailability score | 0.55 |
| Drug-likeness Properties | | |
|  | NP score (Natural product likeness) | 3.196 |
|  | Lipinski rule | Accepted |
|  | Pfizer rule | Accepted |
|  | GSK rule | Rejected |
|  | Golden triangle rule | Accepted |
|  | PAINS | 0 alert(s) |
|  | Ghose | 1 violation(s) |
|  | Veber | 0 violation (s) |
|  | Eegan | 0 violation(s) |
|  | Muegge | 0 violation(s) |
|  | Brenk alerts | 1 Violation(S) |
|  | Lead likeness | 2 Violation(S) |

Here ++ is considered poor and +++ is acclaimed as the worst case.

**Supplementary table S7.** Organ level toxicity profile of Withaferin-A

| **Sr. No** | **Toxicity parameter** | **Inference** |
| --- | --- | --- |
|  | hERG blockers | --- |
|  | H-HT (hepatotoxicity) | -- |
|  | DILI (Drug induced liver injury) | - |
|  | Skin sensitization | --- |
|  | Carcinogenicity | - |
|  | Eye –corrosion/irritation | --- |
|  | Respiratory toxicity | + |
| Toxicity pathways | | |
|  | NR-AR | ++ |
|  | NR-AR-LBD | + |
|  | NR-Aromatase | ++ |
|  | NR-ER | -- |
|  | NR-ER-LBD | ++ |
|  | NR-PPAR-gamma | -- |
|  | SR-MMP | ++ |
|  | SR-p53 | ++ |
| Toxicophore rules | | |
|  | Acute toxicity rule | 0 |
|  | Genotoxic-carcinogenicity rule | 7 |
|  | Non genotoxic carcinogenicity rule | 1 |
|  | Skin sensitization rule | 7 |
|  | Non-biodegradable rule | 3 |
|  | SURE-CHEMBL rule | 2 |
|  | FAF-drugs4 rule | 2 |

Here ++ is considered poor and +++ is acclaimed as the worst case.

**Supplementary table S8.** Correlation between catalytic site amino acids and RMSF values in protein dynamics

| **Protein PDB ID** | **Amino acids present in the catalytic site** | **Amino acids with the highest RMSF values during 100 ns simulation** |
| --- | --- | --- |
| **2JK9** | A:ASP_13, A:THR_35, A:ARG_36, A:LEU_37, A:ASP_38, A:LEU_41, A:LEU_42, A:HID_54, A:ASP_55, A:ASP_57, A:LYS_90, A:VAL_91, A:GLY_92, A: THY_93, A:THR_94, A:ARG_95, A:GLY_96, A:LEU_97, A:ALA_180, A:ASP_182, A:ASP_185, A:THR_187, A:SER_189, A:ILE_191, A:GLY_194, A:TYR_196, A:VAL_199, | A:ASN_155, A:GLN_156, A:PRO_157, A:SER_158, A:LYS_159, A:THR_160, A:TYR_161, A:PRO_162, A:ALA_163, A:PHE_164, A:LEU_165, A:GLU_166, A:PRO_167, A:ASP_168, A:GLU_169, A:THR_170, A:PHE_171, A:ILE_172, A:VAL_173, A:PRO_174, A:ASP_175, A:SER_176, A:PHE_177, A:LEU_178, A:VAL_179 |
| **2VWE** | A:GLN_11, A:ARG_12, A:LYS_13, A:VAL_14, A:VAL_15, A:SER_16, A:ILE_18, A:ASP_19, A:VAL_20, A:THR_22, A:ARG_23, A:THR_25, A:CYS_26, A:GLN_27, A:PRO_28, A:ARG_29, A:GLU_30, A:VAL_31, A:VAL_32, A:VAL_52, A:THR_53, A:VAL_54, A:GLN_55, A:ARG_56, A:CYS_57, A:GLY_58, A:GLY_59, A:CYS_60, A:CYS_61, A:ANS_62, A:ASP_63, A:LEU_66, A:GLU_67, A:CYS_68, A:HIS_74, A:GLN_75, A:VAL_76, A:ARG_77, A:MET_78, A:SER_94, A:GLU_97, A:HIS_98, A:SER_99, A:GLN_100, A:CYS_101, B:GLN_11, B:ARG_12, B:LYS_13, B:VAL_14, B:VAL_15, B:SER_16, B:ILE_18, B:ASP_19, B:VAL_20, B:THR_22, B:ARG_23, B:THR_25, B:GLN_27, B:PRO_28, B:ARG_29, B:GLU_30, B:VAL_52, B:THR_53, B:VAL_54, B:GLN_55, B:CYS_57, B:GLY_58, B:GLY_59, B:HIS_74, B:GLN_75, B:VAL_76, B:ARG_77, B:MET_78, B:GLU_97, B:HIS_98, B:SER_99, C:GLN_37, C:GLN_38, C:LYS_39, C:PRO_40, C:ASP_41, C:GLY_42, C:THR_43, C:VAL_44, C:LYS_45, C:LEU_46, C:LEU_47, C:HIS_55, C:SER_56, C:GLY_57, C:VAL_58, C:PRO_59, C:ARG_61, C:PHE_62, C:GLN_80, C:GLU_81, C:ASP_82, C:ILE_83, C:ALA_84, C:THR_85, C:PHE_87, C:GLY_101, C:LYS_103, C:LEU_104, C:GLU_105, C:ASN_137, C:LYS_141, C:ASP_160, C:SER_161, C:TRP_162, C:THR_163, C:GLU_164, C:GLN_165, C:ASP_166, C:SER_167, C:LYS_168, C:ASP_169, E:GLN_1, E:VAL_2, E:GLN_3, E:GLN_5, E:GLN_6, E:PRO_7, E:GLY_8, E:THR_9, E:GLU_10, E:LEU_11, E:LYS_23, E:ALA_24, E:SER_25, E:GLY_26, E:TYR_27, E:THR_28, E:PHE_29, E:PHE_32, E:GLN_39, E:ARG_40, E:PRO_41, E:GLY_42, E:GLN_43, E:GLY_44, E:SER_76, E:SER_87, E:ALA_88, E:VAL_89, E:TYR_91, E:ARG_94, E:ASP_101, E:TYR_102, E:TRP_103, E:GLY_104, E:GLN_105, E:GLY_106, E:THR_107, E:SER_108, E:VAL_109, E:THR_110, E:SER_112, E:TYR_145, E:PHE_146, E:PRO_147, E:GLU_148, E:PRO_149, E:VAL_150, E:THR_151, E:LEU_152, E:THR_153, E:TRP_154, E:ASN_155, E:SER_156, E:GLY_157, E:SER_158, E:LEU_159, E:SER_160, E:SER_161, E:GLY_162, E:VAL_163, E:HIS_164, E:THR_165, E:PHE_166, E:PRO_167, E:ALA_168, E:LEU_169, E:LEU_170, E:TYR_175, E:LEU_177, E:VAL_183, E:THR_187, E:GLN_191, E:ILE_193, E:ASN_196, E:VAL_197, E:ALA_198, E:LYS_205, E:ASP_207, J:GLN_37, J:GLN_38, J:LYS_39, J:PRO_40, J:ASP_41, J:GLY_42, J:THR_43, J:VAL_44, J:LYS_45, J:LEU_46, J:LEU_47, J:LEU_54, J:HIS_55, J:SER_56, J:GLY_57, J:VAL_58, J:PRO_59, J:SER_60, J:ARG_61, J:PHE_62, J:ASN_77, J:GLU_79, J:GLN_80, J:GLU_81, J:ASP_82, J:ILE_83, J:ALA_84, J:THR_85, J:PHE_87, J:GLY_101, J:LYS_103, J:GLU_105, J:ASN_137, J:LYS_141, J:ASP_160, J:SER_161, J:TRP_162, J:THR_163, J:GLU_164, J:GLN_165, J:ASP_166, J:SER_167, J:LYS_168, J:ASP_169, J:THR_171, J:TYR_172, J:SER_173, L:GLN_1, L:VAL_2, L:GLN_3, L:LEU_4, L:GLN_6, L:GLY_8, L:THR_9, L:GLU_10, L:GLY_26, L:TYR_27, L:THR_28, L:PHE_32, L:GLN_39, L:ARG_40, L:PRO_41, L:GLY_42, L:GLN_43, L:GLY_44, L:SER_87, L:ALA_88, L:VAL_89, L:TYR_91, L:ARG_94, L:ASP_101, L:TYR_102, L:TRP_103, L:GLY_104, L:GLN_105, L:GLY_106, L:SER_108, L:VAL_109, L:THR_110, L:VAL_111, L:SER_112, L:TYR_145, L:PHE_146, L:PRO_147, L:GLU_148, L:PRO_149, L:VAL_150, L:THR_151, L:LEU_152, L:THR_153, L:TRP_154, L:ASN_155, L:SER_156, L:GLY_157, L:SER_158, L:LEU_159, L:SER_160, L:SER_161, L:GLY_162, L:VAL_163, L:HIS_164, L:THR_165, L:PHE_166, L:PRO_167, L:ALA_168, L:LEU_169, L:LEU_170, L:TYR_175, L:LEU_177, L:VAL_183, L:THR_187, L:GLN_191, L:ILE_193, L:PRO_200 | C:ILE_106, C:LYS_106, C:ARG_107, C:ALA_108, C:ASP_109, C:ALA_110, C:ALA_111, C:PRO_112, C:THR_113, C:VAL_114, C:SER_115, C:ILE_116, C:PHE_117, C:PRO_118, C:PRO_119, C:SER_120, C:SER_121, C:GLU_122, C:GLN_123, C:LEU_124, C:THR_125, C:SER_126, C:GLY_127, C:GLY_128, C:ALA_129, C:SER_130, C:VAL_131, C:VAL_132, C:CYS_133, C:PHE_134, C:LEU_135, C:ASN_136, C:PHE_138, C:TYR_139, C:PRO_140, C:GLU_142, C:ILE_143, C:ASN_144, C:VAL_145, C:LYS_146, C:TRP_147, C:LYS_148, C:ILE_149, C:ASP_150, C:GLY_151, C:SER_152, C:GLU_153, C:ARG_154, C:GLN_155, C:ASN_156, C:GLY_157, C:VAL_158, C:SER_170, C:THR_171, C:TYR_172, C:SER_173, C:MET_174, C:SER_175, C:SER_176, C:THR_177, C:LEU_178, C:THR_179, C:LEU_180, C:THR_181, C:LYS_182, C:ASP_183, C:GLU_184, C:TYR_185, C:GLU_186, C:ARG_187, C:HIS_188, C:ASN_189, C:SER_190, C:TYR_191, C:THR_192, C:CYS_193, C:GLU_194, C:ALA_195, C:THR_196, C:HIS_197, C:LYS_198, C:THR_199, C:SER_200, C:THR_201, C:SER_202, C:PRO_203, C:ILE_204, C:VAL_205, C:LYS_206, C:SER_207, C:PHE_208, C:ASN_209, C:ARG_210, C:ASN_211, C:GLU_212, C:CYS_213 |
| **3N1O** | A:GLU_68, A:ILE_71, A:ARG_77, A:PHE_78, A:LYS_79, A:GLU_80, A:LEU_81, A:THR_82, A:PRO_83, A:TYR_85, A:ASN_86, A:PRO_87, A:ILE_89, A:ILE_90, A:PHE_91, A:ARG_101, A:LEU_102, A:THR_104, A:GLN_105, A:ARG_106, A:LYS_108, A:ASP_109, A:ARG_110, A:ASN_112, A: THR_130, A:GLU_131, A:HID_ 139, A:SER_140, A:HIS_149, A:GLU_147, A:ARG_149, A:ASP_152, A:TRP_177, A:TYR_179, A:GLU_181, A:HIS_185, A:HID_185, A:LYS_191, A:SER_192, A:GLU_193, B:TYR_85, B:PRO_87, B:ASP_88, B:ILE_89, B:ILE_90, B:PHE_91, B:GLU_94, C:LYS_43, C:LEU_44, C:VAL_45, C:SER_53, C:PRO_54, C:VAL_56, C:LEU_61, C:GLY_62, C:GLN_105, C:ARG_106, C:ASP_109, C:ARG_110, C:SER_113, C:SER_117, C:ASN_120, C:GLN_121, C:ARG_168, C:LEU_169, C:VAL_171, C:GLU_172, C:ALA_173, C:GLY_174, C:PHE_175, C:ASP_176, C:VAL_178, C:LYS_191, C:SER_192, C:GLU_193 | B:GLU_95, B:ASN_96, B:THR_97, B:GLY_98, B:ALA_99, B:ASP_100, B:ARG_101, B:LEU_102, B:MET_103,B:THR_104, B:GLN_105, B:ARG_106, B:CYS_107, B:LYS_108, B:ASP_109, B:ARG_110, B:LEU_111, B:ASN_112, B:SER_113, B:LEU_114, B:ALA_115, B:ILE_116, B:SER_117, B:VAL_118, B:MET_119, B:ASN_120, B:GLN_121, B:TRP_122, B:PRO_123, B:GLY_124, B:VAL_125, B:LYS_126, B:LEU_127, B:ARG_128, B:VAL_129, B:THR_130, B:GLU_131, B:GLY_132, B:TRP_133, B:ASP_134, B:GLU_135, B:ASP_136, B:GLY_137, B:HIS_138, B:HIS_139, B:SER_140, B:GLU_141, B:GLU_142, B:SER_143, B:LEU_144, B:HIS_145, B:TYR_146, B:GLU_147, B:GLY_148, B:ARG_149, B:ALA_150, B:VAL_151, B:ASP_152, B:ILE_153, B:THR_154, B:THR_155, B:SER_156, B:ASP_157, B:ARG_158, B:ASP_159, B:ARG_160, B:ASN_161, B:LYS_162, B:TYR_163, B:GLY_164, B:LEU_165, B:LEU_166, B:ALA_167, B:ARG_168, B:LEU_169, B:ALA_170, B:VAL_171, B:GLU_172, B:ALA_173, B:GLY_174, B:PHE_175, B:ASP_176, B:TRP_177, B:VAL_178, B:TYR_179, B:TYR_180, B:GLU_181, B:SER_182, B:LYS_183, B:ALA_184, B:HIS_185, B:VAL_186, B:HIS_187, B:CYS_188, B:SER_189, B:VAL_190, B:LYS_191, B:SER_192 |
| **4KMH** | A:VAL_104, A:ASP_403, A:MET_404, A:ALA_405, A:ILE_406, A:THR_407, A:PHE_408, A:VAL_409, A:SER_410, A:THR_411, A:GLY_412, A:VAL_413, A:GLU_414, A:GLY_415, A:ALA_416, A:PHE_417, A:ALA_418, A:THR_419, A:GLU_420, A:GLU_421, A:HIS_422, A:PRO_423, A:TYR_424, A:ALA_425, A:ALA_426, A:HIS_427, A:GLY_428, A:PRO_429, A:TRP_430, A:LEU_431, A:GLN_432, A:ILE_433, A:LEU_434, A:LEU_435, A:THR_436, A:GLU_437, A:GLU_438, A:PHE_439, A:VAL_440, A:GLU_441, A:LYS_442, A:MET_443, A:LEU_444, A:GLU_445, A:ASP_446, A:LEU_447, A:GLU_448, A:ASP_449, A:LEU_450, A:GLU_455, A:PHE_456, A:LYS_457, A:LEU_458, A:PRO_459, A:LYS_460, A:GLU_461, A:TYR_462, A:SER_463, A:TRP_464, A:PRO_465, A:GLU_466, A:LYS_467, A:LYS_468, A:LEU_469, A:LYS_470, A:VAL_471, A:SER_472, A:ILE_473, A:LEU_474, A:PRO_475, A:ASP_476, A:VAL_477, A:VAL_478, A:PHE_479, A:ASP_480, A:SER_481, B:PRO_20, B:THR_21, B:ALA_22, B:PRO_23, B:PRO_24, B:ALA_25, B:PHE_26, B:ALA_27, B:SER_28, B:LEU_29, B:PHE_30, B:PRO_31, B:PRO_32, B:GLY_33, B:LEU_34, B:HIS_35, B:ALA_36, B:ILE_37, B:TYR_38, B:GLY_39, B:GLU_40, B:CYS_41, B:ARG_42, B:ARG_43, B:LEU_44 | A:TYR_60, A:TRP_61, A:LEU_62, A:GLY_63, A:ASP_97, A:ASN_102, A:ARG_103, A:HIS_105, A:GLU_106, A:THR_108, A:ASP_111, A:GLY_112, A:PRO_113, A:GLY_115, A:PHE_116, A:TYR_147, A:PHE_149, A:GLN_150, A:GLU_152, A:ASN_153, A:THR_154, A:PHE_155, A:CYS_156, A:GLY_158, A:ASP_159, A:HIS_160, A:VAL_161, A:SER_162, A:HIS_164, A:LEU_178, A:PHE_192, A:GLU_206, A:LEU_208, A:HIS_209, A:SER_210, A:ALA_211, A:GLN_212, A:GLN_213, A:TRP_214, A:GLY_216, A:THR_243, A:PHE_245, A:GLU_246, A:PRO_249,A:GLN_252, A:GLU_253, A:VAL_255, A:ASP_256, A:ILE_259, A:GLU_260, A:SER_264, A:ASN_265, A:LEU_266, A:SER_267, A:GLY_268, A:VAL_269, A:SER_270, A:GLN_375, A:GLU_376, A:SER_377, A:GLY_378, A:ALA_379, A:LEU_380, A:PRO_382, A:LEU_383,A:ARG_386, A:ARG_388, A:GLY_392, A:ARG_393, A:HIS_394, A:THR_396, A:LYS_398, B:TYR_60, B:TRP_61, B:LEU_62, B:GLY_63, B:GLY_64, B:PRO_65, B:PRO_67, B:ASN_102, B:ARG_103, B:VAL_104, B:HIS_105, B:GLU_106, B:THR_108, B:ASP_111, B:GLY_112, B:PRO_113, B:GLY_115, B:PHE_116, B:ARG_146, B:TYR_147, B:PHE_149, B:GLN_150, B:SER_151, B:GLU_152, B:ASN_153, B:THR_154, B:PHE_155, B:CYS_156, B:SER_157, B:GLY_158, B:ASP_159, B:HIS_160, B:VAL_161, B:SER_162, B:ALA_211, B:GLN_212, B:ASN_215, B:GLY_216, B:GLN_217, B:SER_264, B:ASN_265, B:LEU_266, B:SER_267, B:GLY_268, B:ARG_388, B:GLY_392, B:ARG_393, B:HIS_394, B:THR_396, B:LYS_398, B:VAL_413, B:GLU_414 |
| **4WPB** | A:THR_31, A:PHE_36, A:ILE_46, A:PHE_47, A:LYS_48, A:SER_50, A:LEU_66, A:MET_81, A:ILE_83, A:PRO_85, A:GLN_89, B:PHE_17, B:TYR_21, B:TYR_25, B:GLY_59, B:CYS_61, B:ASN_62, B:ASP_63, B:GLU_64, D:ALA_18, D:ASP_20, D:PRO_21, D:LEU_23, D:ASN_24, D:ASP_25, D:PHE_28, D:HIS_29 | B:MET_94, B:SER_95, B:PHE_96, B:LEU_97, B:GLN_98, B:HIS_99, B:ASN_100, B:LYS_101, B:CYS_102, B:GLU_103, B:CYS_104, B:ARG_105, B:PRO_106, C:GLU_8, C:CYS_10, C:ASN_11, C:ARG_13, C:ALA_14, C:ILE_15, C:GLU_16, C:AIB_17, C:ALA_18, C:LEU_19, C:ASP_20, C:PRO_21, C:ASN_22 |
